# Plasticity of an Ultrafast Interaction between Nucleoporins and Nuclear Transport Receptors

**DOI:** 10.1016/j.cell.2015.09.047

**Published:** 2015-10-22

**Authors:** Sigrid Milles, Davide Mercadante, Iker Valle Aramburu, Malene Ringkjøbing Jensen, Niccolò Banterle, Christine Koehler, Swati Tyagi, Jane Clarke, Sarah L. Shammas, Martin Blackledge, Frauke Gräter, Edward A. Lemke

**Affiliations:** 1 Structural and Computational Biology Unit, Cell Biology and Biophysics Unit, European Molecular Biology Laboratory (EMBL), Meyerhofstrasse 1, 69117 Heidelberg, Germany; 2 Molecular Biomechanics group, HITS gGmbH, Schloß-Wolfsbrunnenweg 35, 69118 Heidelberg, Germany; 3 IWR – Interdisciplinary Center for Scientific Computing, Im Neuenheimer Feld 368, 69120, Heidelberg, Germany; 4 University Grenoble Alpes, IBS, F-38044 Grenoble, France; 5 CNRS, IBS, F-38044 Grenoble, France; 6 CEA, IBS, F-38044 Grenoble, France; 7 Department of Chemistry, University of Cambridge, Cambridge CB2 1EW, UK

## Abstract

The mechanisms by which intrinsically disordered proteins engage in rapid and highly selective binding is a subject of considerable interest and represents a central paradigm to nuclear pore complex (NPC) function, where nuclear transport receptors (NTRs) move through the NPC by binding disordered phenylalanine-glycine-rich nucleoporins (FG-Nups). Combining single-molecule fluorescence, molecular simulations, and nuclear magnetic resonance, we show that a rapidly fluctuating FG-Nup populates an ensemble of conformations that are prone to bind NTRs with near diffusion-limited on rates, as shown by stopped-flow kinetic measurements. This is achieved using multiple, minimalistic, low-affinity binding motifs that are in rapid exchange when engaging with the NTR, allowing the FG-Nup to maintain an unexpectedly high plasticity in its bound state. We propose that these exceptional physical characteristics enable a rapid and specific transport mechanism in the physiological context, a notion supported by single molecule in-cell assays on intact NPCs.

## Highlights

• Integrative structural biology reveals the basis of rapid nuclear transport
• Transient binding of disordered nucleoporins leaves their plasticity unaffected
• Multiple minimalistic low-affinity binding motifs create a polyvalent complex
• A highly reactive and dynamic surface permits an ultrafast binding mechanism

## Graphical Abstract

**Figure:**
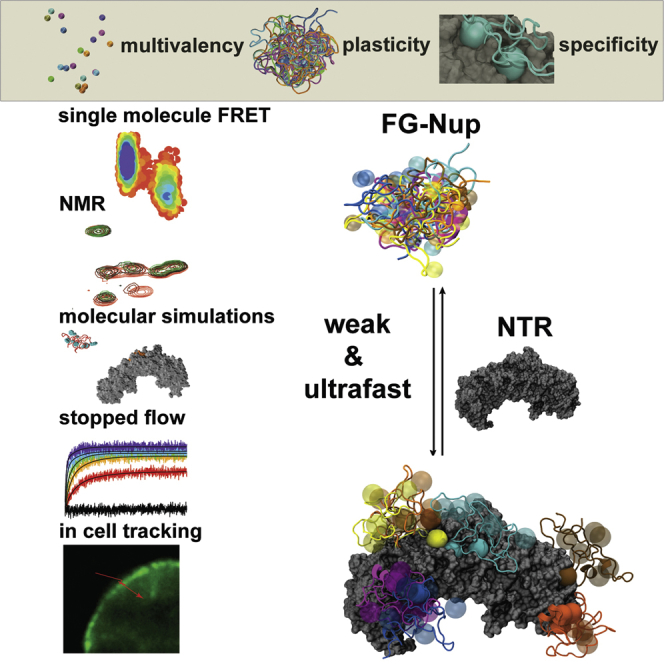

## Teaser

Intrinsically disordered nucleoporins (Nups) engage rapidly with nuclear transport receptors through many minimalistic, weakly binding motifs. These Nups form polyvalent complexes while retaining conformational plasticity thus ensuring both rapid and specific transport.

## Introduction

The plasticity of intrinsically disordered proteins (IDPs) is thought to be key to their highly diverse roles in the eukaryotic interactome and a variety of vital processes such as transcription, epigenetic regulation mechanisms, and transport through nuclear pore complexes (NPCs) (Dyson and Wright, 2005; Tompa and Fuxreiter, 2008). The central channel of the NPC is filled with phenylalanine-glycine-rich proteins, called FG-nucleoporins (FG-Nups) that are intrinsically disordered (Denning et al., 2003). FG-Nups build up an approximately 30-nm-thick permeability barrier through which large molecules (>40 kDa) can only be shuttled when bound to a nuclear transport receptor (NTR) with passage times as fast as 5 ms (Hoelz et al., 2011; Kubitscheck et al., 2005; Tu et al., 2013; Wälde and Kehlenbach, 2010). Due to the intrinsic dynamics of the FG-Nups, even state-of-the-art electron tomographic studies are not able to visualize them within the central NPC channel, despite their millimolar concentrations (Bui et al., 2013). Consequently, the molecular structure of the permeability barrier and its general mode of action are widely debated (for a review see Adams and Wente, 2013).

The key to understanding the observed nucleocytoplasmic transport phenomena resides in a description of the binding mode between FG-Nups and NTRs, for which a molecular analysis of the FG-Nup⋅NTR interaction is a prerequisite. Our current understanding of the molecular basis of FG-Nup⋅NTR interactions is in large part derived from X-ray crystallographic structures or molecular dynamics (MD) simulations of NTRs in the presence of short FG-peptides (up to ∼13 amino acids in length) (Bayliss et al., 2000; Isgro and Schulten, 2005), as well as binding measurements with different NTRs or mutated NTR binding pockets (Bednenko et al., 2003; Milles and Lemke, 2014; Otsuka et al., 2008). Even for FG-Nups alone, only overall chain dimensions or long-range interactions within the Nups have so far been analyzed in solution (Milles and Lemke, 2011; Yamada et al., 2010). Notably, even such fundamental binding characteristics as the equilibrium dissociation constant (K_d_) between Nups and NTRs are still matter of discussion - estimates range from a few nM to several mM (Bednenko et al., 2003; Ben-Efraim and Gerace, 2001; Tetenbaum-Novatt et al., 2012; Tu et al., 2013). However, high K_d_ (low affinity, ∼mM) values are not easily compatible with high specificity of the transport process, while low K_d_ values (∼nM range) cannot easily explain high transport rates, since these might be expected to correlate with long residence times whereas NTRs must encounter many FG-Nups while crossing the thick barrier.

Fast protein binding also typically requires proper orientation of the protein binding partners as well as conformational adaption of the IDP to bind to a folded protein. Those can occur prior to or during binding, as described by either of the two prevalent models for protein binding namely conformational selection and induced fit (Csermely et al., 2010; Wright and Dyson, 2009). While such a conformational shift or fit can present the rate-limiting step of binding, fast binding is warranted in many biological processes. Several binding rate enhancing effects have been suggested or observed experimentally, such as maintenance of a degree of disorder (termed “fuzziness”; Tompa and Fuxreiter, 2008) by conformational funneling (Schneider et al., 2015), a large capture radius of the flexible IDPs (Shoemaker et al., 2000), and the involvement of long-range electrostatic interactions to steer (attract) proteins together (Ganguly et al., 2013).

In this work, we characterize the conformational plasticity of Nups from human and yeast in the presence of structurally and functionally diverse NTRs. A focus was a PxFG-rich domain of the Nup153 (Nup153FG^PxFG^) as its size permitted a combination of nuclear magnetic resonance (NMR), single molecule Förster resonance energy transfer (smFRET), and molecular dynamics (MD) simulations to characterize local, residue specific, as well as long-range implications of Importinβ binding to Nup153FG^PxFG^ conformation and dynamics. Additional Brownian dynamics (BD), fluorescence stopped-flow and single molecule transport experiments with functional NPCs in permeabilized cells, revealed the detailed kinetics of the complex formation between Nup and NTR. Using this molecular, integrative structural biology approach we propose a mechanism whereby Nups contribute low-affinity minimalistic binding motifs that act in concert to create a polyvalent complex. The global Nup structure and dynamics are largely unaffected by the interaction, thereby ensuring ultrafast binding and unbinding of individual motifs—a result that explains how nuclear transport can be fast yet specific, and that may have general implications for the mechanism of action of other IDPs that exhibit a multiplicity of binding motifs.

## Results

### Nup153FG^PxFG^ Populates a Disordered Ensemble in Solution

**Figure 1:**
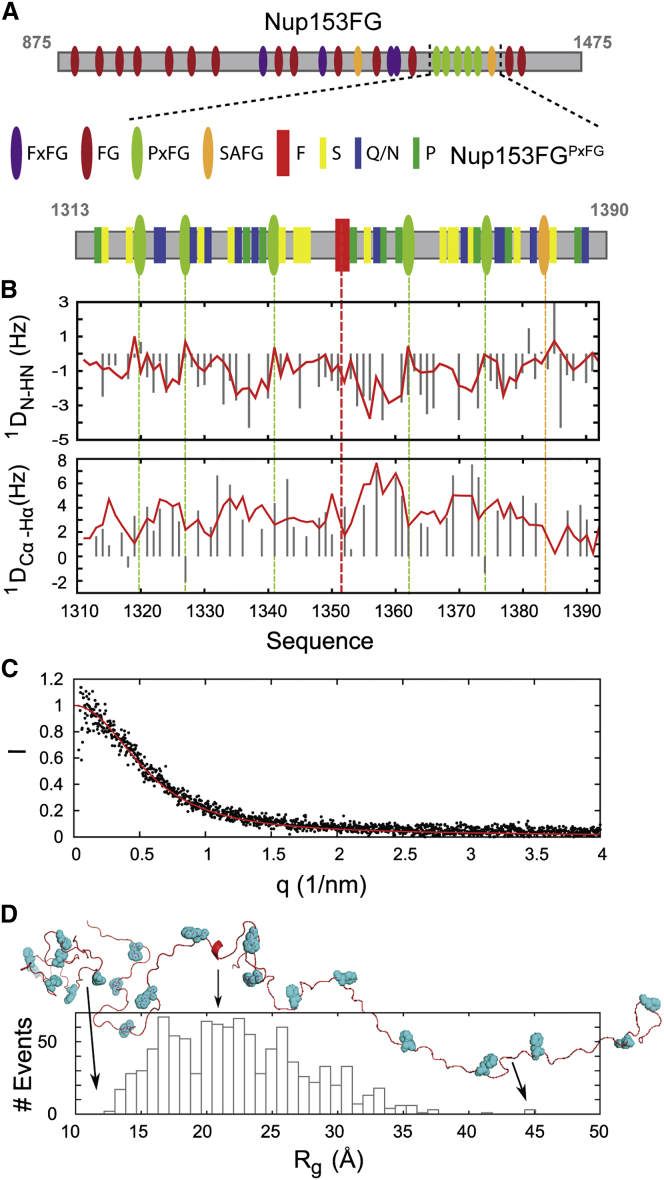
Conformation of Nup153FG^PxFG^ (A) Scheme of Nup153FG constructs. (B) Residual dipolar couplings (RDCs) of Nup153FG^PxFG^ aligned in phages. Experimentally obtained RDCs (gray bars) were compared with RDCs calculated from the ASTEROIDS ensemble obtained on the basis of experimental chemical shifts (red line). Dashed lines represent positions of FG-repeats and F1374. Color code as in (A). (C) The same conformational ensemble was used to calculate a small angle X-ray scattering (SAXS) curve using CRYSOL (red line). The back calculated scattering curve is in good agreement with measured SAXS data under similar experimental conditions (black dots) (Mercadante et al., 2015). (D) Distribution of the radius of gyration (R_G_) from five equivalent ASTEROIDS selections. The three conformations displayed on top represent the most compact, the least compact, and one of the most prevalent conformations in the ensemble.

**Figure S1:**
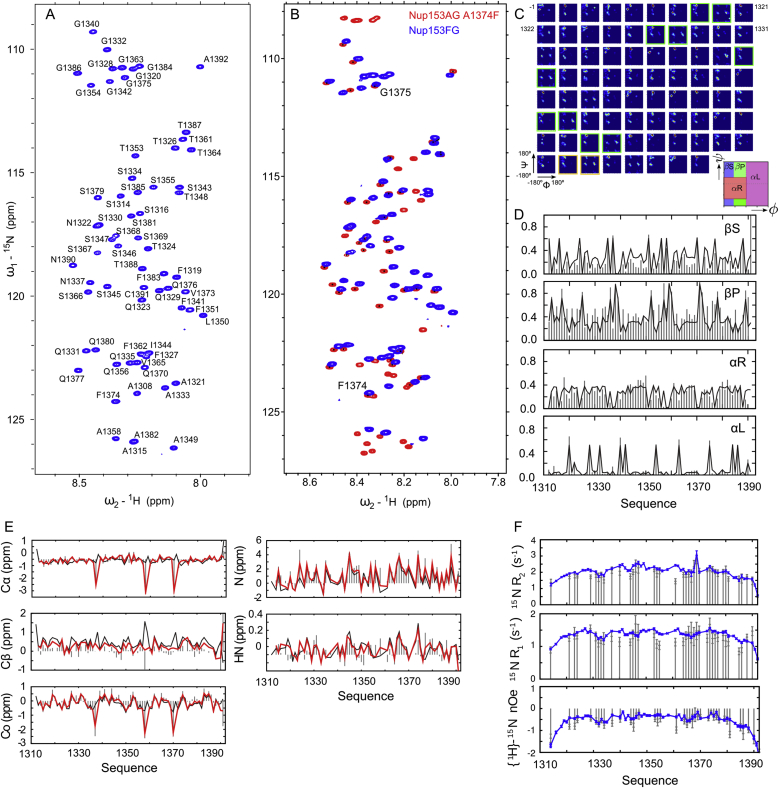
Conformational Behavior of Nup153FG^PxFG^/ Nup153FG^PxFG,F1374^, Related to Figure 1 (A) Assignment of the ^1^H-^15^N HSQC spectrum of Nup153FG^PxFG^. Amino acid numbers refer to the sequence as in Uniprot P49790 (see sequences in Supplemental Experimental Procedures). Note that residues 1391 and 1392 refer to Cys and Ala for cloning and fluorescence labeling purposes rather than serines. (B) Characterization of Nup153AG^PxAG, F1374^. Nup153AG^PxAG, F1374 1^H-^15^N HSQC spectrum (red) overlaid with a spectrum of Nup153FG^PxFG^ (blue). The only F and neighboring G in the remaining FG-motif, as well as all amino acids with sufficient distances to mutated residues display very similar chemical shifts in the AG compared to the FG variant of the protein. (C–E) Atomic resolution ensemble description of Nup153FG^PxFG^ based on experimental NMR data. (C) Ramachandran maps for all Nup153FG^PxFG^ residues as obtained from the ASTEROIDS selection. FG-repeats are color-coded as in Figure 1 (A). (D) Local conformational sampling of Nup153FG^PxFG^ showing the population of four regions of Ramachandran space (βS: β sheet; βP: Polyproline; αR and αL: right and left handed alpha helices). Gray bars result from the ASTEROIDS ensemble selected using chemical shifts. Black lines illustrate the conformational sampling of the initial statistical coil ensemble. (E) Nup153FG^PxFG^ secondary chemical shifts obtained from experiments (gray bars) and ASTEROIDS selection from a Flexible Meccano ensemble (red), based on the experimental chemical shifts. Black lines represent the initial ensemble, prior to selection. (F) Local dynamic behavior of Nup153FG^PxFG^ and Nup153AG^PxAG, F1374^. ^15^N R_2_, ^15^N R_1_, and {^1^H}-^15^N steady-state heteronuclear Overhauser effects of Nup153AG^PxAG, F1374^ (gray bars) at a ^1^H frequency of 600 MHz and 25°C compared to Nup153FG^PxFG^ (blue lines), showing very similar fast (ps-ns) timescale dynamics, characteristic of intrinsically disordered proteins.

We initially characterized the structure and dynamics of Nup153FG^PxFG^ using high resolution NMR (Figure 1A, sequences given in Supplemental Experimental Procedure). Complete assignment of the backbone resonances (Figure S1) allowed us to develop a multi-conformational model of the protein in solution using a combination of Flexible-Meccano (Ozenne et al., 2012) and the genetic algorithm ASTEROIDS (Jensen et al., 2010). Representative ensembles comprising 200 conformers were selected on the basis of the experimental chemical shifts and were in excellent agreement with ^1^D_N-NH_ and ^1^D_Cα-Hα_ residual dipolar couplings and small angle X-ray scattering (SAXS) curves (Mercadante et al., 2015) that were not used in the selection process (Figures 1B–1D). The amino acid specific backbone dihedral angle distributions determined from the ensemble selections (Figure S1) show that negligible secondary structure is present.

### Global Structure and Dynamics of the Nup153FG^PxFG^ Are Retained upon Interaction with Importinβ as Measured by smFRET

We labeled Nup153FG^PxFG^ with a donor (Alexa488) and acceptor dye (Alexa594) for FRET at its C- and N terminus, respectively. This allowed us to measure average distance between the dyes as well as the dynamic properties of the protein using histograms relating FRET efficiency (E_FRET_) and donor lifetimes (τ) of single molecules (sm), a method widely used to detect even minute changes in structure and dynamics, for example when IDPs bind, fold or expand (Kalinin et al., 2010; Milles and Lemke, 2011; Schuler and Eaton, 2008).

**Figure 2:**
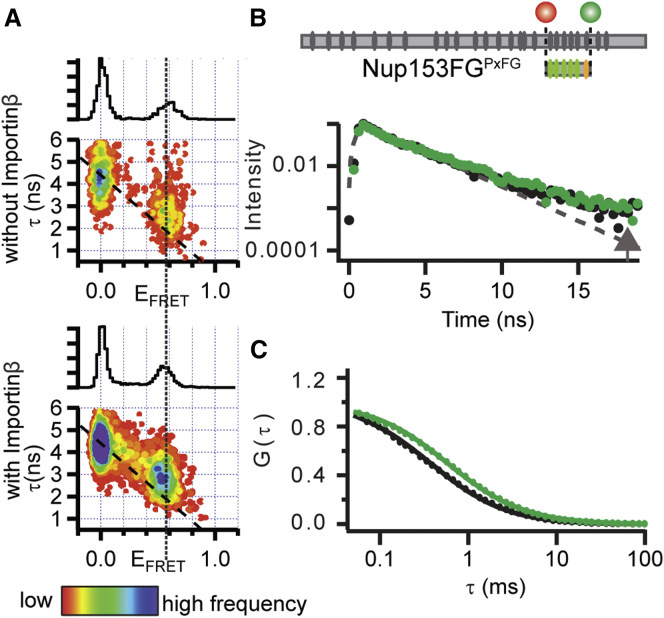
Nup153FG^PxFG^⋅Importinβ Interaction Analyzed by smFRET (A) FRET efficiency (E_FRET_) versus fluorescence lifetime (τ) histograms of Nup153FG^PxFG^ in the presence and absence of Importinβ. The dotted line visualizes the center position of the FRET peak. The dashed (diagonal) lines show the static E_FRET_ relationship, on which a distribution would lie in the absence of fast dynamics. (B) Fluorescence lifetimes (τ) of the double labeled population accumulated from single molecule data in the absence (black) and presence (green) of Importinβ. Offset from a single exponential lifetime (dashed gray curve and arrow) is a strong indicator of protein dynamics. (C) Fluorescence correlation spectroscopy (FCS) traces retrieved from measurements of Nup153FG^PxFG^ (black dots) reflect a slower translational motion in the presence of Importinβ (green dots).

**Figure S2:**
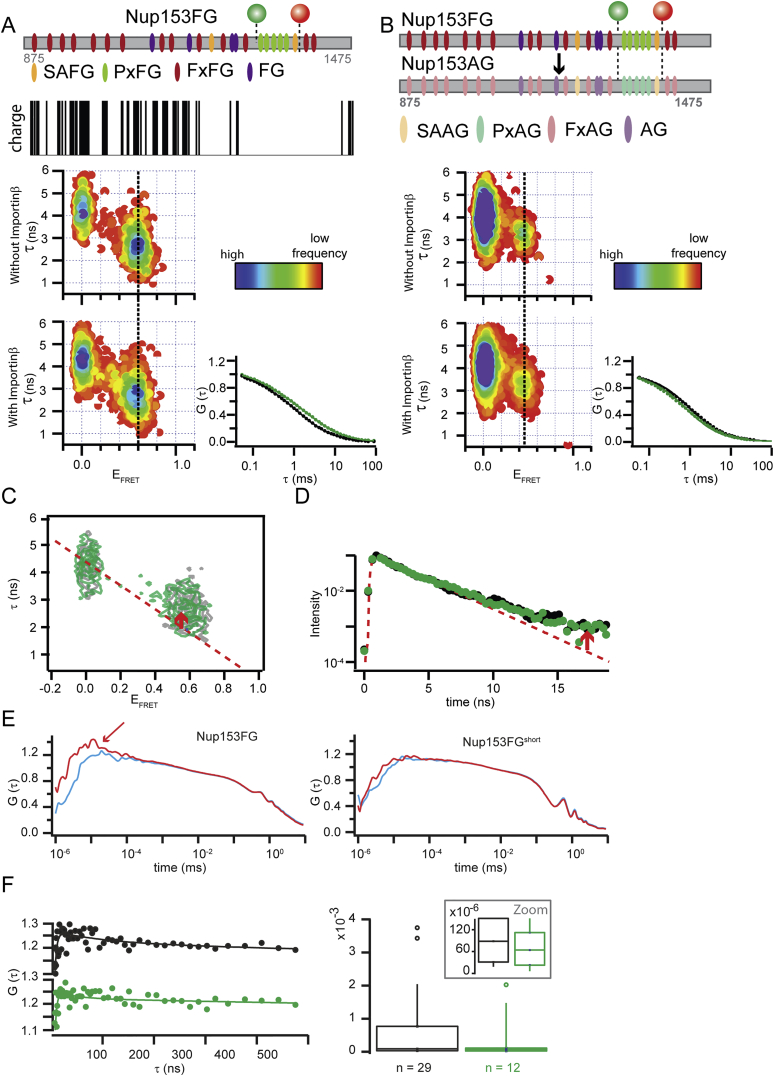
Structure and Dynamics of Nup153FG, PxFG-rich region, in Presence and Absence of Importinβ and Controls, Related to Figure 2 (A) Schemes of (601 aa full FG domain) Nup153FG A1391TAG S1312C FRET mutant (probing the PxFG-rich region) and Nup153FG^PxFG^. Numbers indicate the selected amino acid range with respect to the full-length Nup153FG (UniProt: P49790). Donor (green, Alexa488) and acceptor (red, Alexa594) were attached on the genetically encoded unnatural amino acid acetyl-phenylalanine (AcF) and a cysteine, respectively. The 2D histograms show burst integrated τ versus E_FRET_ histograms of a double-labeled Nup153FG. The plots are color-coded for frequency of occurrence. Top and right histograms are projections along the τ and E_FRET_ axis respectively. Changes in ratio between the so called donor-only peak (arising from molecules that contain no active acceptor) and the FRET peak typically originate from photophysical effects and different labeling efficiencies between the different sample preparation of full-length Nup153FG and Nup153FG^PxFG^ (see also Figure S3 for additional effects contributing to differences in observed number of events). Also shown are fluorescence correlation spectroscopy (FCS, G(τ)) traces retrieved from the same measurements (black curves). Those show slower translational motion in the presence of Importinβ (green curves). (B) Analog to (A), but data shown for an all F to A mutant termed Nup153AG, which binds Importinβ less well than the WT. (C) Burst integrated fluorescence lifetime (τ) versus FRET efficiency (E_FRET_) histograms of a double-labeled Nup153FG in the presence (green) and absence (gray) of Importinβ are overlaid for comparison. Dashed lines represent the static FRET lines and red arrows indicate deviations due to donor-acceptor dynamics. (D) Fluorescence lifetimes (τ) of the double-labeled population accumulated from single molecule data in the absence (black) and presence (green) of Importinβ. The single exponential lifetime fit (dashed curve) does not adequately fit the fluorescence lifetime decay (red arrow), which is a strong indicator for protein dynamics. (E and F) Measurements of donor-acceptor dynamics timescales are non-trivial and have in the past been extracted from a combination of nanosecond resolved FCS (nsFCS) and smFRET for a few small IDPs (Soranno et al., 2012). We measured donor-acceptor dynamics of FRET labeled Nup153FG and Nup153FG^PxFG^ by splitting polarized light of the donor light channel onto three detectors. Parallel polarized light (||) was detected on two detectors (each recording 50% of the total intensity in the || channel), perpendicular light (⊥) on one detector. These experiments were performed with a higher protein concentration of 5 nM. || light was correlated with || light (red curves) and with ⊥ light (blue curves). The marked difference of red and blue curves in the case of Nup153FG (left plot) indicated that—due to the large size of Nup153FG compared to most previously studied proteins—fluorescence anisotropy significantly hampers analysis of the correlation curves. Small contributions from donor-acceptor fluctuations can therefore not easily be extracted. A similar observation was previously made for bigger protein complexes in accordance with our interpretation (Hillger et al., 2008). Nup153FG^PxFG^ (E, right plot), however, a much smaller protein, does not pose these difficulties and correlation times can therefore be analyzed: The curves in which || light was correlated with ⊥ light were first fit with a simple diffusion model (down to ∼0.3 μs) in which the triplet component of the donor was extracted. The correlation curves were then normalized and plotted with a linear scale up to 500 ns (F, dots in left panel). These curves were then fit (solid lines) with the following model: *G(t) = (1-α∙exp(t/τα))^∗^(1+β∙exp(t/ τ*_*β*_*))(1+γ∙exp(t/τ*_*γ*_*)),* with component α the antibunching, component β the FRET dynamics and γ the triplet component, where *τ*_*γ*_ was fixed as extracted from diffusion fits. The right panel in (F) displays a statistical analysis (median 89 ns and 65 ns; 25^th^ quantiles 31 ns and 23 ns, 75^th^ quantile 768 ns and 112 ns without (black) and with (green) Importinβ, respectively) of the obtained fit values (note that amplitudes are so small that the fitting error is rather large). Correlations were performed with SymPhoTime (Picoquant, Berlin, Germany).

We added unlabeled Importinβ to the FRET labeled Nup153FG^PxFG^ and followed the smFRET response. While the diffusion of Nup153FG^PxFG^ in the absence and presence of Importinβ confirmed the binding of Importinβ under single molecule conditions (Figures 2 and S2), we detected neither substantial changes in E_FRET_ nor in the width of the histograms indicating absence of significant changes in the distance distribution (Figure S2 shows an all F to all A negative control). Indeed, the E_FRET_ populations of the unbound and bound Nup153FG^PxFG^ also overlay very closely with respect to τ, which indicates similarly fast dynamics of both forms (Figures 2 and S2 for detailed analysis of structure and dynamics) (Kalinin et al., 2010).

As smFRET is compatible with large proteins, we were able to repeat the same experiments for the same PxFG region within the full-length Nup153FG (601 amino acids), finding similar characteristics, and suggesting that our truncated Nup153FG^PxFG^ largely retains the conformational sampling from within the whole Nup153FG (Figure S2).

**Figure S3:**
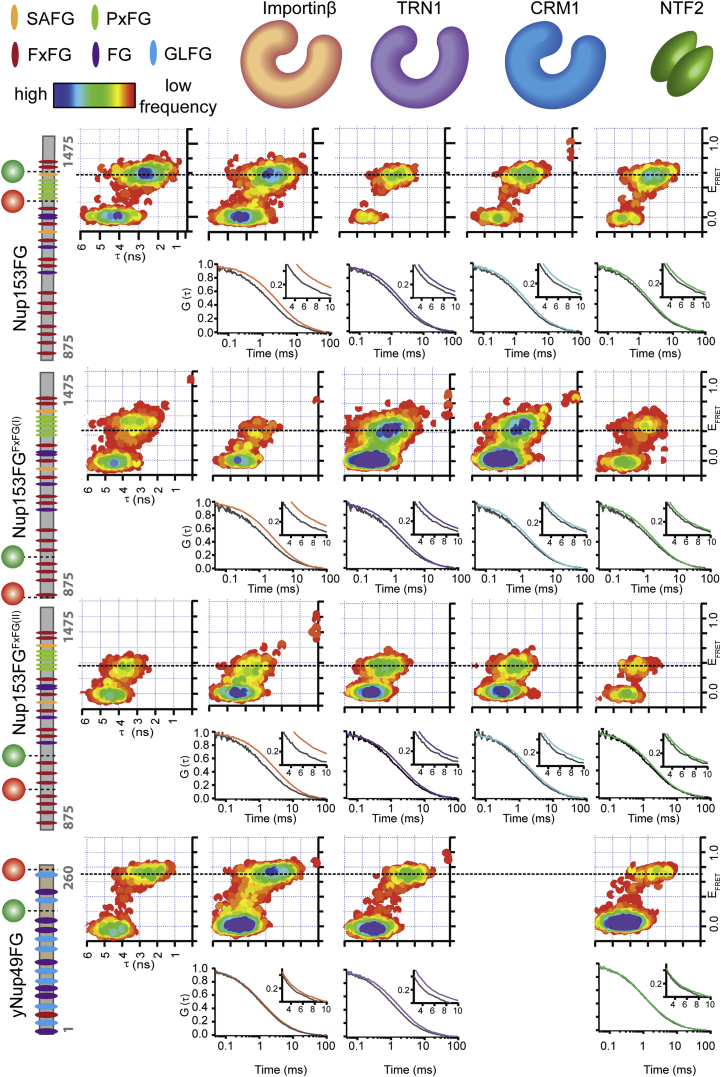
Conformation of Different Nucleoporin Regions in the Presence and Absence of Different NTRs Probed by smFRET, Related to Figure 2 This figure shows a cartoon of the labeling site in the respective Nup (Nup153FG or yNup49FG) on the left, as well as the resulting 2D histograms (τ versus E_FRET_) in the presence of the respective NTR (depicted on top: Importinβ [orange], TRN1 [purple], CRM1 [blue] and NTF2 [green]). In the smFRET experiments, the Nup was used at pM concentration, and the NTR at 1 μM. Also shown are FCS experiments, were the Nups was used at 10 nM concentration. In summary, FCS detects that overall Nups are binding competent to all tested NTRs, while in the smFRET measurements no substantial conformational changes were observed in agreement with the main conclusion of this work. Note that only in the yNup49FG experiments, NTR concentration was increased to 10 μM, due to an overall lower binding affinity. However, high background in the CRM1 sample preparation did not permit high resolution single molecule FRET measurements anymore at such high concentration, and was thus excluded from this one analysis. Note also that the absolute values for FRET, depend among other factors mainly on the amino spacing of the dyes, which were different for the different constructs. Furthermore, a burst search algorithm was used to select signals from noise with a total photon count >100. In such an analysis it is inherent that the absolute values (intensities, i.e., number of events) for smFRET peaks differ depending on e.g., diffusion time (different for different sized complexes) different background (due to slight contamination from different preparations), sample sticking issues, etc.

In order to determine the general nature of this binding mode, we repeated the experiments with two different FxFG-rich regions of Nup153FG, as well as the GLFG-rich yeast Nup49 and several different NTRs: i) transportin 1 (TRN1), a transport receptor involved in the import of proteins containing an M9 recognition sequence, ii) nuclear transport factor 2 (NTF2), the import receptor of RanGDP and iii) chromosomal region maintenance 1 (CRM1), a major exportin. While TRN1 and CRM1 have a similar molecular weight and superhelical structure as Importinβ, NTF2 is a much smaller, β sheet-rich dimer (Cook et al., 2007; Morrison et al., 2003). As detailed in Figure S3, despite the very distinct functionalities of the different NTRs, the smFRET and FCS measurements of the different Nups and NTRs indicate similar binding characteristics as for the Nup153FG⋅Importinβ complex.

### Interaction with Importinβ Influences Nup153FG^PxFG^ Only Locally and Transiently

**Figure 3:**
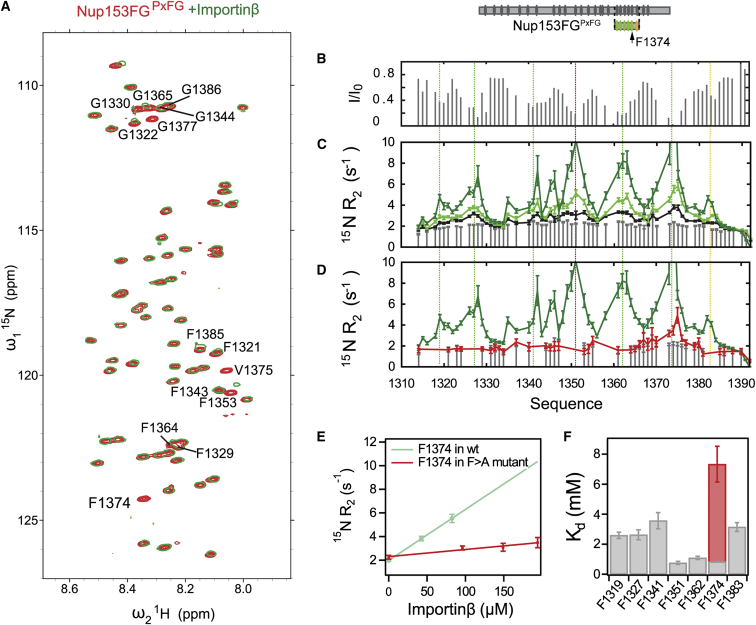
Nup153FG^PxFG^⋅Importinβ Interaction by NMR Spectroscopy (A) ^1^H-^15^N HSQC spectrum of Nup153FG^PxFG^ (red) overlayed with a spectrum of Nup153FG^PxFG^ in the presence of Importinβ (green, Nup to NTR molar ratio of 1.14, at a Nup concentration of 240 μM). (B) The intensity ratio of the bound and unbound form of Nup153FG^PxFG^ was plotted under the same conditions as in (A). (C) ^15^N R_2_ relaxation rates at 25°C and a ^1^H frequency of 600 MHz were measured at different concentrations of Importinβ (gray bars are without Importinβ; black, light green and dark green at Importinβ/Nup153FG^PxFG^ molar ratios of 0.17, 0.33, and 0.72 at the constant Nup153FG^PxFG^ concentration of 250 μM). (D) ^15^N R_2_ of Nup153AG^PxAG, F1374^ in the absence (gray) and in the presence of Importinβ (red) overlayed with the rates for Nup153FG^PxFG^ in the presence of Importinβ under the same conditions (green). (E) For all F in the Nup153FG^PxFG^ sequence, ^15^N R_2_ values were plotted against Importinβ concentration and fitted with a linear slope. The same analysis was performed for F1374 in Nup153AG^PxAG, F1374^ and compared to the same F in Nup153FG^PxFG^ (compare red to green slope). R2 with errors greater than 20% were excluded from the analysis. (F) Local K_d_ values were calculated from the slopes obtained in Figure S4. Gray bars correspond to K_d_ values obtained from Nup153FG^PxFG^, the red bar shows the local K_d_ of Nup153AG^PxAG, F1374^ binding to Importinβ. Error bars show SD.

**Figure S4:**
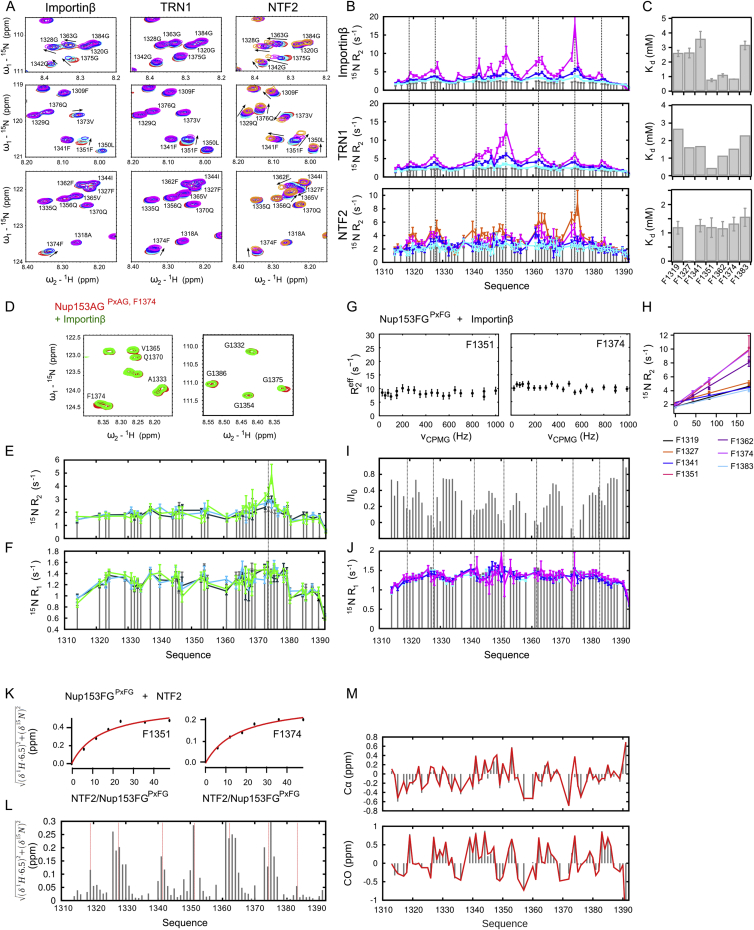
Interaction of Nup153FG^PxFG^ with NTRs Related to Figure 3 (A) Titration of Nup153FG^PxFG^ with different transport receptors. ^1^H-^15^N HSQC spectra of Nup153FG^PxFG^ in the presence of different concentrations of Importinβ (left row), TRN1 (middle row) and NTF2 (right row) were overlayed. Importinβ was added at a molar excess of 0.17 (light blue), 0.33 (dark blue) and 0.72 (magenta), TRN1 at a molar excess of 0.17 (light blue), 0.33 (dark blue), and 0.61 (magenta), and NTF2 at a molar excess of 0.33 (light blue), 1 (dark blue), 2 (magenta), and 4 (orange). Nup153FG^PxFG^ was kept at a concentration of about 250 μM throughout the titration. The spectrum of the unbound Nup153FG^PxFG^ is displayed in red. Chemical shift changes are indicated by black arrows. Spectral differences are clearly localized to the same amino acids in all cases. For NTF2 it was experimentally possible to access much higher molar ratios, and the molecular weight of the partner is much smaller than Importinβ and TRN1, explaining why larger shifts are observed. (B) Relaxation of Nup153FG^PxFG^ at different NTR mixtures. ^15^N R_2_ of Nup153FG^PxFG^ in the presence of different concentrations of NTRs (concentrations as described in A; colored in the order of increasing NTR concentration as light blue, dark blue, magenta, and orange). ^15^N R_2_ of Nup153FG^PxFG^ alone are displayed as gray bars. (C) Residue specific K_d_ values. Residue specific K_d_ values were extracted from the R_2_ rates as described in the Supplemental Experimental Procedures. The rotational correlation time of Nup153FG^PxFG^ bound to TRN1 was assumed to be the same as when bound to Importinβ, an assumption that is justified by the similar architecture of the two transport receptors and their similar molecular weight. Since we do not have an estimate for the rotational correlation time of Nup153FG^PxFG^ bound to NTF2, we extracted the residue specific K_d_ values from the absolute chemical shift changes from a titration of Nup153FG^PxFG^ with NTF2 to near saturation. (D–F) Binding of Nup153AG^PxAG, F1374^ to Importinβ. (D) Illustration of chemical shift changes upon interaction of ^15^N labeled Nup153AG^PxAG, F1374^ with Importinβ. ^1^H-^15^N HSQC of Nup153AG^PxAG, F1374^ (red) overlayed with a spectrum of Nup153AG^PxAG, F1374^ in the presence of Importinβ (green, Nup at 250 μM, Importinβ at 190 μM). (E) ^15^N R_2_ relaxation rates of Nup153FG^PxAG^ were measured at different concentrations of Importinβ (gray bars are without Importinβ; dark green, blue, and light green at Importinβ/Nup153AG^PxAG, F1374^ molar ratios of 0.38, 0.6, and 0.76 at a constant Nup153AG^PxAG, F1374^ concentration of 250 μM). Dashed line indicates the position of F1374. (F) Associated ^15^N R_1_ relaxation rates (see E). (G) Relaxation dispersion of Nup153FG^PxFG^ when interacting with Importinβ. CPMG relaxation dispersion measurements were performed with Nup153FG^PxFG^ bound to Importinβ at a stoichiometry ratio of 0.72 (Importinβ/nucleoporin; Nup concentration was 250 μM). The effective R_2_ (R_2_^eff^) at different refocusing frequencies (υ_cpmg_) is plotted for two representative Fs in the Nup153FG^PxFG^ sequence. (H) Estimation of local dissociation constants. ^15^N R_2_ values plotted against Importinβ concentration (molar ratios of 0.17, 0.133, and 0.72 at the constant Nup153FG^PxFG^ concentration of 250 μM) and fitted with a slope from which FG specific K_d_ values were extracted under the assumption that the local rotation time (τ_c_) of Nup153FG^PxFG^ bound to Importinβ equals τ_c_ of Importinβ itself. (I) Intensity ratio of the bound and unbound form of Nup153FG^PxFG^ at a Nup concentration of 250 μM and Importinβ at 180 μM as obtained from two ^1^H-^15^N HSQC spectra. (J) Characterization of the structure and dynamics of Nup153FG^PxFG^ in complex with Importinβ. ^15^N R_1_ of Nup153FG^PxFG^ at different concentrations of Importinβ (gray bars - without Importinβ; light blue, dark blue, magenta at Importinβ/Nup153FG^PxFG^ molar ratios of 0.17, 0.133, and 0.72 at the constant Nup153FG^PxFG^ concentration of 250 μM, as in B). (K) Chemical shift titration of Nup153FG^PxFG^ with NTF2. Residue specific K_d_ values derived from the absolute chemical shift changes from a titration of Nup153FG^PxFG^ with NTF2. The data were fit with a simple binding model under the assumption of excess NTF2 (see Supplemental Experimental Procedures). Shown are titration curves for two representative Fs. (L) Chemical shift titration of Nup153FG^PxFG^ with NTF2. Absolute chemical shift changes mapped for Nup153FG^PxFG^ in the presence of 4-fold excess of NTF2. (M) Cα and CO secondary chemical shifts obtained from Nup153FG^PxFG^ alone (gray bars) and in the presence of 24-fold excess of NTF2 at a Nup153FG^PxFG^ concentration of 80 μM (red curve), demonstrating that local conformational sampling is conserved upon interaction. Note that prolines are excluded from this plot. Error bars show SD.

To characterize the effects of Importinβ binding on Nup153FG^PxFG^ at atomic resolution, we titrated Importinβ into a solution of ^15^N labeled Nup153FG^PxFG^ and measured ^1^H-^15^N HSQC spectra at different molar ratios. Peak intensities, as well as ^1^H^N^ and ^15^N chemical shifts of Nup153FG^PxFG^, were analyzed for each titration step (Figures 3 and S4). Resonance line broadening, associated with small changes in both ^1^H^N^ and ^15^N chemical shifts, was observed around all F’s in the Nup sequence (Figure 3A). Binding was clearly highly localized, and limited to F’s, with only F and the immediately adjacent amino acids being affected by the interaction. Interestingly, one single F, which is not associated with a G, is also involved in binding to Importinβ, showing the largest chemical shift changes in the ^1^H-^15^N HSQC spectrum during titration with Importinβ (Figure 3A and S4). ^15^N relaxation rates measured as a function of molar ratio of Importinβ suggest that, overall, the molecule remains flexible in the complex with the transverse relaxation (R_2_) increasing significantly upon Importinβ titration only around the interaction sites (Figures 3C and S4), in agreement with the above smFRET-based observations that global disorder and flexibility are not affected by Importinβ binding. Carr-Purcell-Meiboom-Gill (CPMG) relaxation dispersion experiments (Figure S4) suggested that fast exchange (< 10 μs) between the bound and unbound form of Nup153FG^PxFG^ gives rise to the increased R_2_ rates around the interaction sites, which makes it possible to estimate a residue-specific K_d,individual_ for each position in Nup153FG^PxFG^ with Importinβ (Figures 3E, 3F and S4) from the population weighted R_2_ measurements. Interestingly, the FG-specific affinities to Importinβ are not identical across the Nup153FG^PxFG^ sequence, implying a contribution of inter-FG residues to binding, although all FG-specific K_d,individual_ values lie in the millimolar range.

Strikingly, when studying the binding to different NTRs like TRN1 and NTF2 (Figure S4), despite exhibiting different binding preferences for FG-Nups (Cook et al., 2007; Milles and Lemke, 2014), their binding modes are remarkably similar to that of the Importinβ complex. The same regions in Nup153FG^PxFG^ are affected by the interaction, again with very low residue specific affinities, with the Nup remaining overall flexible when bound while interacting only locally as seen from both chemical shift changes, in the case of NTF2, and remarkably similar locally elevated transverse relaxation rates in TRN1 (Figure S4). Comparison of ^13^C backbone chemical shifts measured in the free and NTF2-bound forms of Nup153FG^PxFG^ demonstrates that the protein backbone remains flexible upon interaction, sampling effectively the same conformational equilibrium in the free and bound state (Figure S4).

We note that during the publication process of this work, localized interaction was also reported for the yeast Nsp1 with Kap95 (the yeast homolog of Importinβ) using NMR (Hough et al., 2015), suggesting that a similar interaction mechanism may also be conserved across species.

### Co-operativity of FG-Nup⋅Importinβ Binding

To further quantify the action of multiple FG-repeats, we designed a Nup construct, in which all F of Nup153FG^PxFG^ except F1374, the strongest interaction site for Importinβ, were replaced by A (Figure S1). Titration of Importinβ into this Nup153AG^PxAG,F1374^ mutant resulted in strongly reduced peak broadening and negligible chemical shift changes compared to Nup153FG^PxFG^ (Figure S4). As in the case of Nup153FG^PxFG^, ^15^N R_2_ relaxation rates of Nup153AG^PxAG,F1374^ at the interaction site exhibited a linear dependence on Importinβ concentration (Figure 3E). However the effective K_d,individual_ from F1374 within Nup153AG^PxAG,F1374^ reveals significantly weaker binding for this interaction site than for F1374 when situated within the wild-type (WT) protein (K_d,individual_ = 7.3 mM compared to 0.8 mM, Figure 3). This result clearly shows that presenting multiple equivalent binding sites to the binding partner has a measurably positive effect on the effective affinity of the individual interaction site.

### Monitoring the Nup153FG^PxFG^⋅Importinβ Binding Using All-Atom MD

**Figure 4:**
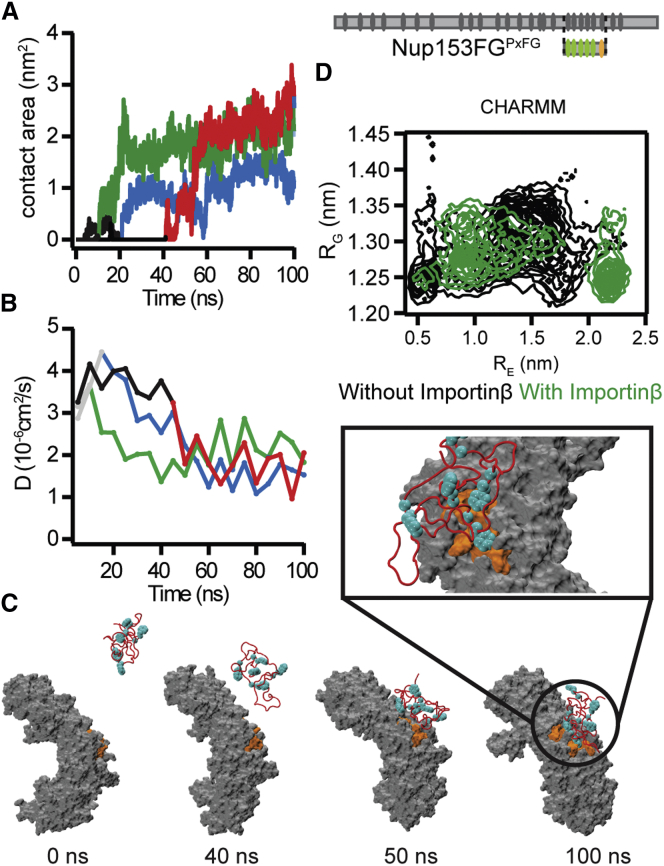
Binding of Nup153FG^PxFG^ to Importinβ^N^ (A–C) Contact area between (A) Nup153FG^PxFG^ and Importinβ^N^ and (B) diffusion coefficients D as a function of time for the 4 binding events out of 10 simulations (gray/black: prior to binding; different colors: after binding; black/red curves refer to the cartoon in (C) sampled using CHARMM22^∗^ force field. (C) Snapshots collected along one of the recorded MD trajectories showing the binding between Nup153FG^PxFG^ (red cartoon) and Importinβ^N^ (gray surface). The binding sites on Importinβ^N^ and Nup153FG^PxFG^ FG-repeats are colored in orange and cyan respectively. (D) Nup153FG^PxFG^ radius of gyration (R_G_) as a function of end-to-end distance (R_E_) for the unbound (black) and bound (green) ensembles of Nup153FG^PxFG^ obtained from the simulations performed using CHARMM22^∗^. See Figure S5 for data using the AMBER force field.

**Figure S5:**
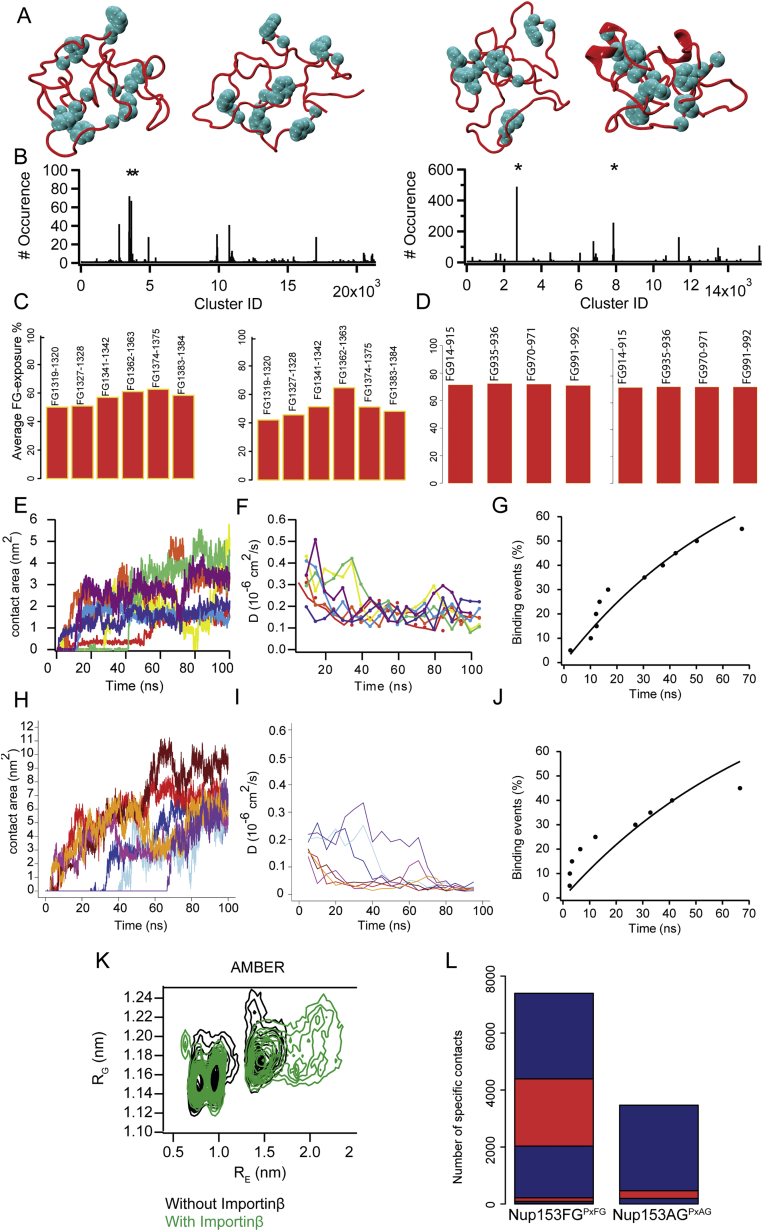
Computational Analysis of Nup153FG^PxFG^ and Nup153FG^FxFG^ Dynamics and of Their Binding to Importinβ^N^, Related to Figure 4 (A) Nup153FG^PxFG^ conformations (means of the cluster) that have been selected from MD simulations in the absence of Importinβ^N^ using AMBER99-sb^∗^-ILDN (left) and CHARMM22^∗^ (right). FG-repeats are shown by their van der Walls (vdW) radius. (B) Count for each cluster of the simulated conformations of Nup153FG^PxFG^ as simulated with AMBER99-sb^∗^-ILDN (left) CHARMM22^∗^ (right). Stars indicate the clusters corresponding to the conformations represented in (A). (C and D) Average solvent exposure of Nup153FG^PxFG^ (C) and Nup153FG^FxFG^ (D) FG-repeats estimated from molecular dynamics trajectories collected from molecular dynamics simulations performed using AMBER99-sb^∗^-ILDN (C - left panel) and CHARMM22^∗^ (D, left) force fields. The average degree of exposure has been calculated as the ratio between the mean SASA of the FG-repeat within the equilibrated Nup153FG^PxFG/FxFG^ ensembles and of an FG-repeat not forming any tertiary contacts. (E and H) Contact areas between Nup153FG^PxFG^ (E) or Nup153FG^FxFG^ (H) and Importinβ^N^ as a function of time for the binding events observed in the MD simulations performed using AMBER99-sb^∗^-ILDN force field. (F and I) Diffusion coefficients D, of Nup153FG^PxFG^ (F) and Nup153FG^FxFG^ (I) as a function of time are reported for the same replicas shown in (E) and (H). The drop in the diffusion coefficient is coincident with the increase in contact area upon the binding of the Nup153FG^PxFG^ and Nup153FG^FxFG^ to Importinβ^N^. (G and J) Percentage of binding as a function of the binding time observed in the MD runs for the simulations reporting the binding of Nup153FG^PxFG^ (G) or Nup153FG^FxFG^ (J) to Importinβ^N^. The fit of (solid line for Nup153FG^PxFG^ - Importinβ^N^ and for Nup153FG^FxFG^ - Importinβ^N^ association) such a cumulative distribution of binding events gives a rough estimate of the k_on, MD_ of approximately 10^10^ M^−1^s^−1^. We note that due to the low sampling and known lower friction of water and other force field issues, (Mercadante et al., 2015) the results form MD rather define an upper limit. (K) Nup153FG^PxFG^ radius of gyration (R_G_) as a function of end-to-end distance (R_E_) for the unbound (black) and bound (green) ensembles of Nup153FG^PxFG^ obtained from the simulations performed using the AMBER99-sb^∗^-ILDN force field. (L) Count of specific contacts has been performed for the simulations of Nup153FG^PxFG^ and Nup153AG^PxAG^ (in which all F residues were mutated to A) binding to Importinβ^N^. Blue and red bars indicate the five different simulations performed in each case using the AMBER99-sb^∗^-ILDN force field. In the case of the Nup153AG^PxAG^, two out of five simulations did not yield any specific contact between the partners. Specific contacts have been considered as any contact occurring within a cutoff distance of 0.6 nm between the FG/AG-repeats of Nup153FG/AG^PxFG/PxAG^ and any binding site on Importinβ^N^.

**Figure S6:**
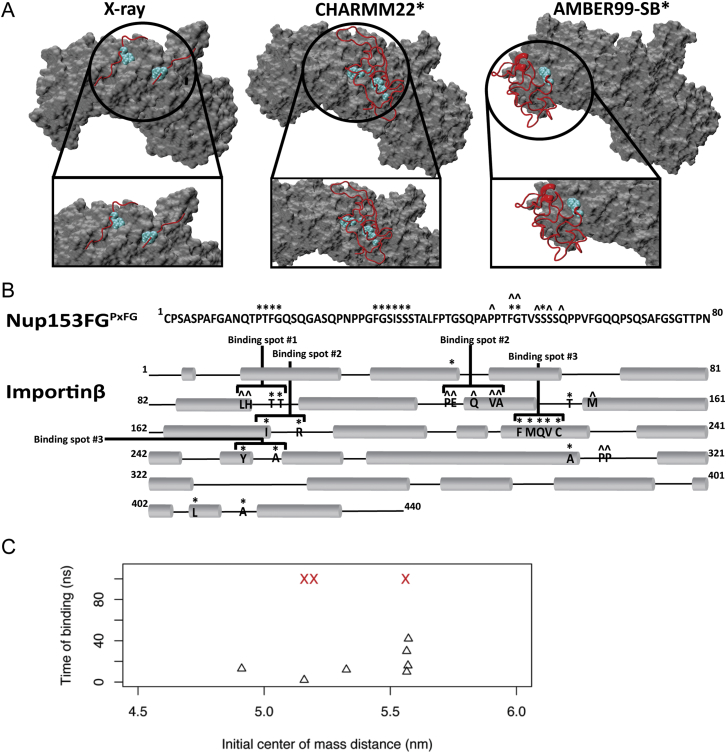
MD Analysis of the Nup153FG^PxFG^⋅Importinβ^N^ Complex, Related to Figure 4 (A) Comparison of the Nup153FG^PxFG^-Importinβ^N^ complexes observed in X-ray crystallographic studies (PDB: 1F59) (left), MD simulations performed using the CHARMM22^∗^ (center panel) and the AMBER99-sb^∗^-ILDN (right) force fields. The surface of Importinβ^N^ is shown in gray whereas Nup153FG^PxFG^ is shown in red. FG-repeats are shown in cyan. (B) Interaction sites of Nup153FG^PXFG^ and Importinβ^N^. “^∗^” and “ˆ” symbols account for the contacts observed in the CHARMM22^∗^ and AMBER99-sb^∗^-ILDN simulations respectively. Grey cylinders represent the α helices along the structure of Importinβ^N^. (C) Distance between the center of mass (COM) of Nup153FG^PxFG^ and Importinβ^N^ as a function of the observed time of binding. The upper triangles show simulations reporting successful binding events whereas red crosses show simulations in which binding between the partners has not been observed during the simulated time.

We employed MD simulations to investigate the experimental observations of Nup153FG^PxFG^⋅Importinβ association from NMR and smFRET. From a broad ensemble of Nup153FG^PxFG^ obtained from unbiased MD simulations in explicit solvent (Movie S1), we incubated different conformers with the N-terminal portion of Importinβ (from here named Importinβ^N^ (Bayliss et al., 2000)) and monitored their binding for a total simulation time of 2 μs (Figures S5 and S6, and Table S1). The association of Nup153FG^PxFG^ to Importinβ^N^ was repeatedly observed within the simulated timescale and occurred in a specific manner (Figures 4 and S5, and Movie S2). FG-repeats docked into previously identified binding pockets on the surface of Importinβ^N^ and even formed contacts similar to those previously observed crystallographically upon interaction between Importinβ and Nsp1-derived peptides (Figures 4C and S6) (Bayliss et al., 2000). Binding was reduced and less specific for Nup153FG^PxAG^ (Figure S5), in agreement with NMR and smFRET (Figures S1, S2, and S4).

We suggest that the high solvent exposure of Fs in the unbound state (typically contained within the hydrophobic interior of folded proteins) (Figure S5) renders them readily available for Nup153FG^PxFG^⋅Importinβ^N^ association, without requiring any global structural transitions in either partner (Figures 4D, S6, Movie S2).

The ability to monitor spontaneous Nup153FG^PxFG^⋅Importinβ^N^ association on the sub-microsecond timescale suggests an ultrafast association (Figure S5). Underlining the generality of our observation, we were also able to monitor such a spontaneous binding event when repeating simulations for an FxFG-rich region of Nup153 binding to Importinβ^N^ (Figure S5, Movie S3, sequences given in Supplemental Experimental Procedure). However, force field inaccuracies and limited sampling prohibit the reliable extraction of an association rate, and we therefore studied the interaction further through fluorescence stopped-flow experiments (FSF) and Brownian dynamics (BD) simulations.

### FSF Experiments and BD Simulations Reveal Ultrafast Binding between Nup and Importinβ

**Figure 5:**
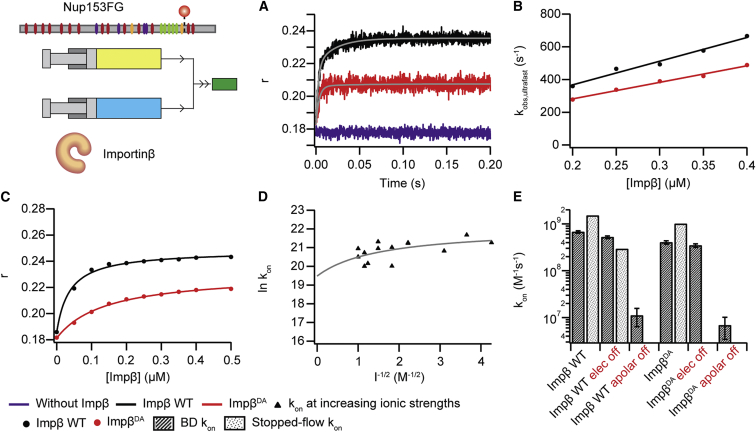
Association Kinetics for Nup153FG with Importinβ (A) Stopped-flow fluorescence anisotropy was used to monitor the binding of Importinβ (Impβ) at different concentrations to Nup153FG-Cy3B. A selection of anisotropy (r) traces against time is shown for Nup153FG alone (purple) and for the binding of Importinβ WT (black) and Importinβ^DA^ (red). (B) The observed rates (k_obs,ultrafast_) from association experiments were plotted against the different Importinβ concentrations, the data were linearly fitted to obtain the association rate constants (k_on,ultrafast_). (C) Apparent K_d,app_ values under the different experimental conditions. (D) k_on_ obtained from association experiments of Nup153FG and Importinβ at different ionic strengths fitted with a Debye-Hückel-like approximation to calculate the basal rate constant at infinite ionic strength. (E) Summary of the k_on_ values obtained from BD (dark bars) and FSF measurements (light bars) (Table S2D). Error bars show SD.

**Figure S7:**
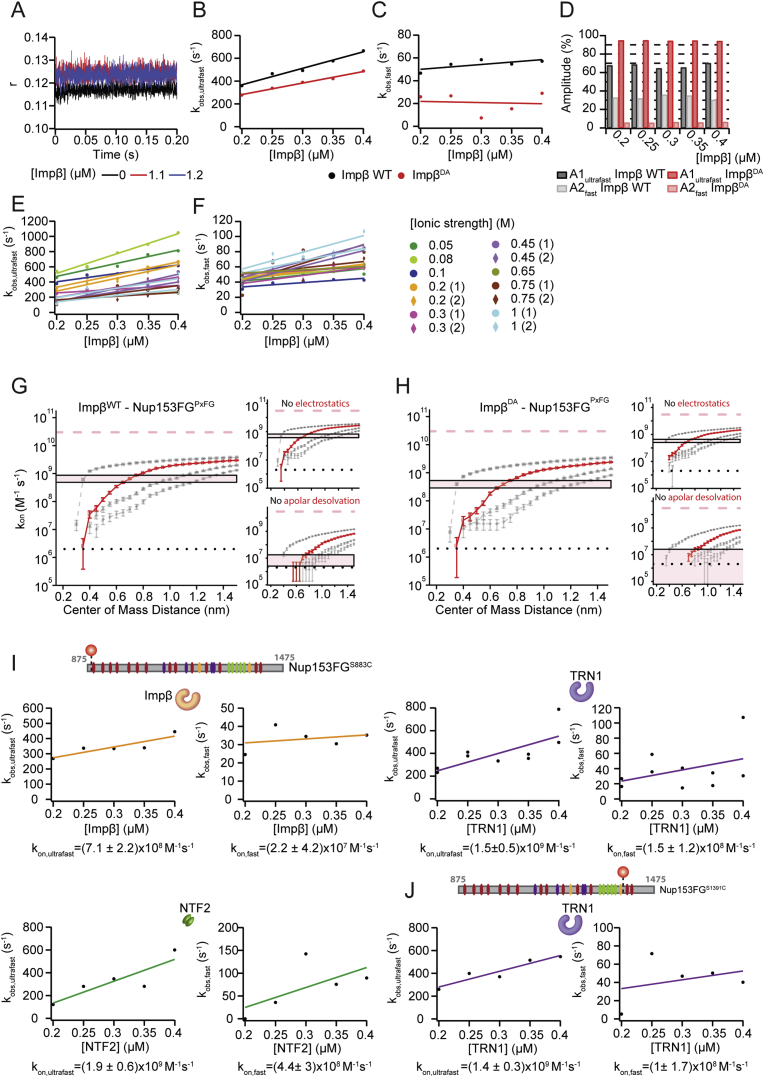
Association Stopped-Flow Experiments of Nup153FG and Nup153FG^PxFG^ to NTRs, Related to Figure 5 (A) Association experiment of Nup153FG^PxFG^ with Importinβ WT, a selection of anisotropy traces is shown. At the tested conditions no kinetic changes were detected upon binding. (B–F) The observed rates from association experiments of Nup153FG to Importinβ WT (black) and double mutant (red) from the major phase (k_obs,ultrafast_) (B) and the minor phase (k_obs,fast_) (C) are plotted against the different Importinβ concentrations. The amplitudes A1_ultrafast_ and A2_fast_ correspond to the amplitudes from the fit associated to k_obs,ultrafast_ and k_obs,fast_ respectively (D). Bars indicating A1_ultrafast_ and A2_fast_ are shown for each measurement at the different concentrations of Importinβ WT and Importinβ^DA^ mutant. Nup153FG and Importinβ WT were also tested at different ionic strength conditions, the observed rates of the ultrafast component (k_obs,ultrafast_) (E) and of the fast component (k_obs,fast_) (F) obtained from different ionic strengths: 0.05 M (dark green), 0.08 M (light green) 0.1 M (blue), 0.2 M (orange),0.3 M (pink), 0.45 M (purple), 0.65 (green khaki), 0.75 M (brown) and 1 M (cyan) are plotted against Importinβ concentration, numbers in brackets correspond to independent replicate measurements. As discussed in detail in (Milles and Lemke, 2014) anisotropy changes in such a disordered system depend on a variety of parameters, including degree of segmental motion and multivalency. As we have several degrees of multivalency (several FG repeats in the Nup, several binding sites in the NTR, and the ability to form higher order complexes than 1:1) the exact origin of the slower component cannot be assessed. In line with related studies a likely origin could be additional binding of NTRs (Wagner et al., 2015). As such FSF measurements report on overall formation of Nup153FG⋅Importinβ complex i.e., one or more F binding. The fastest k_on_ measured defines even a lower limit for the first F binding. However, we note that both observed reaction rates (major and minor phase) are remarkably fast, and all our conclusions are also valid if one considers only the minor phase. (G and H) Association rates obtained from Brownian Dynamics simulations. The curves report the estimated k_on_ as a function of the contact distance between the binding partners when a different number of independent contacts (between 1 to 4) are formed between Importinβ^N^ or Importinβ^N,DA^ and Nup153^PxFG^. The red curves report the cases in which two reaction criteria are satisfied. The condition in which two reaction criteria are satisfied at a distance of 0.7 nm has been used to extrapolate the value of estimated association rate. Pink shaded rectangles show the range of k_on_ when two contacts between 0.65 and 0.75 nm are formed. The simulations were performed considering Nup153FG^PxFG^ interacting with Importinβ^N^ (G) or Importinβ^N,DA^ (H) and considering all interactions contributing to the binding or in the absence of electrostatic interactions or apolar desolvation. (I and J) Association kinetics for Nup153FG with different NTRs probed by stopped-flow. (I) Here we show the observed rates (k_obs,ultrafast_ and k_obs,fast_ from association experiments with Importinβ (orange), TRN1 (purple) and NTF2 (dark green) to Nup153FG Cy3B labeled at the positions 883C, probing an FxFG enriched region. The obtained association constants (k_on,ultrafast_, k_on,fast_) from the slope of the linear fit are indicated on the plots. (J) Analog to Figure 5, we shown here in addition the observed rates (k_obs,ultrafast_ and k_obs,fast_ from stopped-flow association experiments with TRN1 (purple) to Nup153FG Cy3B labeled at the positions 1391C (probing a PxFG enriched region). The obtained association constants (k_on_) from the linear fit are indicated on the plots and summarized in Table S2**C**. We note that the observed rates are at the limit of what is detectable with advanced stopped-flow instrumentation. The signal to noise in the observed anisotropy *r* depends on a variety of parameters, the labeling site, the multivalency, the binding strength, the degree of segmental motion etc. as detailed in (Milles and Lemke, 2014). In line with the observed lower affinity of the used transport receptors for yNup49 (data not shown), signal to noise was not sufficient to perform high resolution stopped-flow measurements of this Nup. Perfectly in line with our results reported in Figure 5, for all those different NTRs and different FG regions we observed remarkably fast rates consistently with a k_on,ultrafast_ > 5·10^8^ M^−1^s^−1^. All data are summarized in Table S2C.

Stopped-flow kinetics monitoring fluorescence anisotropy (*r*) can be used to study binding mechanisms and measure the association rate (k_on_) between proteins (Shammas et al., 2013). The binding of Importinβ to Nup153FG site-specifically labeled with Cy3B elicits detectable changes in *r*, due to slowed rotational motion (Milles and Lemke, 2014). Since Nup153FG^PxFG^ has only a very small overall binding affinity toward Importinβ, we could not detect a sufficiently strong signal change in the anisotropy measurements in the tested and experimentally feasible concentration range (Figure S7). Thus, for FSF, we used fluorescently labeled full-length Nup153FG. We performed rapid mixing experiments under pseudo-first order conditions in “physiological” transport buffer. A monoexponential function does not describe well the observed anisotropy changes in Figure 5 (Figure S7 and Table S2). This is likely a result of having multiple different binding motifs and/or the ability of multiple Importinβ to engage into binding a single Nup, which adds another level of complexity (multivalency) (Milles and Lemke, 2014; Schoch et al., 2012; Wagner et al., 2015). A biexponential equation is able to describe the kinetics, resulting in two k_obs_ per Importinβ concentration (Figures 5A, 5B, and S7). The fluorescence anisotropy at the end of the reaction was used to calculate the apparent K_d,app_ (Figure 5C). Remarkably, by performing experiments at multiple NTR concentrations we extracted an ultrafast k_on,ultrafast_ = 1.5·10^9^ M^−1^s^−1^ (Figure 5B) for the major component (average amplitude of 70%), while the second component was still very fast, with a k_on,fast_ = 6.1·10^7^ M^−1^s^−1^ at room temperature. These FSF measurements report on overall formation of the Nup153FG⋅ Importinβ complex i.e., one or more F binding. While we provide all results and further analysis details in Figure S7 and Table S2, for later discussion we focus on the fastest measured k_on,ultrafast_.

We next estimated association rates from BD simulations, which compared to MD permit larger statistical sampling, at the cost of freezing the internal dynamics of the binding partners. Upon successful complex formation, starting from the conformations obtained from MD, the association rate was estimated (Figure S7) to be around 10^9^ M^−1^ s^−1^ (Figure 5E), in agreement with stopped-flow measurements.

BD simulations carried out without the contribution of apolar desolvation generated a drastic decrease of the estimated k_on,BD_ by around two orders of magnitude, while the absence of electrostatic interactions had a negligible effect (Figures 5E and S7, and Table S2D and S2E). These observations complement our evidence for an association mainly favored by the energetic gain of sequestrating F residues from the solvent and burying them into the Importinβ^N^ binding pockets.

While desolvation effects cannot easily be tested experimentally, high ionic strength buffers can be used to shield long-range electrostatic interactions. We thus performed a salt titration ranging from 0.05 to 1 M ionic strength (using NaCl), permitting an estimate of k_on_ under infinite electrostatic shielding by extrapolation using a Debye-Hückel-like approximation (Figures 5D and S7 and Table S2B) (Shammas et al., 2014). In line with the BD simulations, we obtained a k_on,elect off_ of 2.9·10^8^ M^−1^s^−1^, i.e., binding remains very fast even under electrostatic shielding.

Additional stopped-flow measurements probing different Nup153FG regions (FxFG-, PxFG-rich) with diverse NTRs (NTF2, TRN1, Importinβ) are shown in Figure S7 and Table S2C. In all cases, we observed similar remarkably fast kinetics yielding consistent results for k_on_ > 5·10^8^ M^−1^s^−1^.

Previously, solid phase binding assays indicated that the Importinβ double mutant (I178D/Y255A, termed Importinβ^DA^) has a more than 60-fold lower K_d_ for binding to full-length Nup153FG as compared to Importinβ WT (Bednenko et al., 2003). k_on,BD_ dropped by only 40% compared to Importinβ WT, which we confirmed by experimental FSF studies (drop of k_on,FSF_ by 30%, Figure 5). However, fluorescence anisotropy measurements revealed an Importinβ^DA^ titration curve (Figure 5C) that confirms altered binding as compared to Importinβ WT, as e.g., due to an increase in k_off_.

### Single-Particle Tracking Connects Nuclear Transport of Importinβ^DA^ and Importinβ with FG-Nup Association Rates

**Figure 6:**
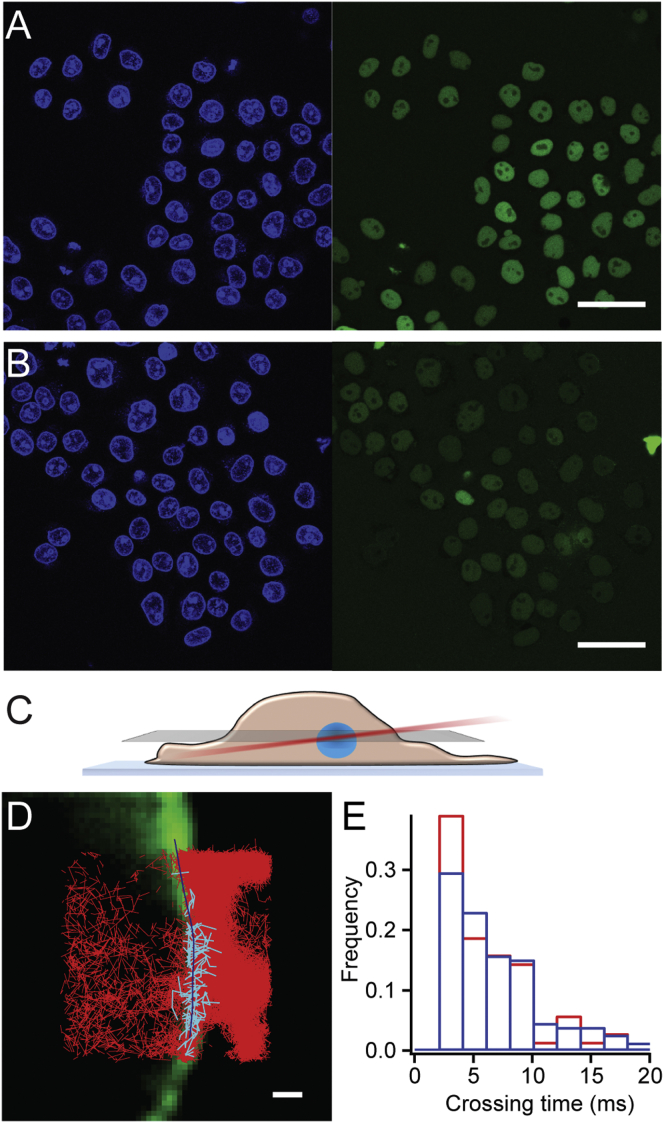
Nuclear Transport Assays of Importinβ and Importinβ^DA^ (A and B) DAPI staining shown in blue, and green fluorescent cargo (NLS-MBP-eGFP) in permeabilized HeLa cells incubated with either Importinβ (A) or Importinβ^DA^ (B) (scale bar 50 μm). After 45 min, cargo accumulation is higher in the nucleus in (A). (C) Single molecule trajectories of fluorescently labeled Importinβ were acquired in the equatorial plane of the nucleus exploiting an inclined (Hilo) illumination. (D) Representative image of acquired single molecule trajectories of Importinβ-Alexa488 (red lines) overlaid with the ensemble image of Importinβ-Alexa647 (in green, scale bar 1μm) used to identify the nuclear envelope position (blue line). Single particle tracks of the fluorescently labeled NTR (cyan lines) crossing the nuclear envelope were analyzed to yield the characteristic barrier crossing time. (E) The crossing time distributions reported for Importinβ (blue bars) and Importinβ^DA^ (red bars) are very fast.

The efficiency of an NTR to bring cargo across the NPC barrier can be assayed using standard NPC transport assays. In these assays, a fluorescent cargo (NLS-MBP-eGFP) recognized by the Importinβ transport machinery is incubated with permeabilized cells in the presence of a functional transport system and the resulting nuclear fluorescence is measured. In line with the previously reported lower K_d_ of Importinβ^DA^, cargo accumulated slower compared to Importinβ WT measurements (Figures 6A and 6B) which can e.g., be due to a lower barrier crossing time, a reduced docking efficiency to the NPC or cargo release from the NPC for example.

A prediction from our kinetic analysis is that the actual speed of barrier crossing, which involves several binding and unbinding steps between NTR and FG repeats should be rather similar for WT and mutant Importinβ, as changes in k_on_ were small, and if at all, a higher k_off_ for the mutant would make crossing even faster (see discussion).

In contrast to the “bulk” transport assay, the speed of barrier crossing (characteristic crossing time) can be measured directly using single molecule (sm) tracking assays (Figure 6C), in which individual Importinβ molecules are fluorescently labeled and tracked while they cross from one side of the NPC to the other. This yielded a typical value of 6.9 ± 0.2 ms for Importinβ and 6.1 ± 0.5 ms for Importinβ^DA^ for barrier crossing (Figures 6D and 6E). We note that this crossing time is near the sampling limit of our technology, and thus faster crossing times cannot easily be captured.

## Discussion

The realization that many proteins are disordered has attracted considerable attention to the study of the molecular mechanisms controlling their interactions (Csermely et al., 2010; Tompa and Fuxreiter, 2008; Wright and Dyson, 2009), including the role of disorder in promoting or facilitating binding. In particular, very little is known about the binding mechanisms involved in complex processes such as nucleocytoplasmic transport, where NTRs have to engage in multiple, specific binding and unbinding events while traversing a tens of nanometer thick permeability barrier.

In this study, we have used a multidisciplinary approach to investigate the molecular mechanism underlying the interaction process between NTRs and Nups. In general, from our three core findings a coherent view emerges on how multiple rapid, yet specific protein interactions can be achieved.

### Nup153FG Forms a Highly Dynamic Complex with Importinβ

Based on our smFRET measurements, we found that Nup153FG^PxFG^ resembles full-length Nup153FG with respect to its dynamics (Figures 2 and S2). Upon interaction with Importinβ, Nup153FG^PxFG^ remains flexible, engaging with Importinβ only locally, as is evident from peak broadening in the respective ^1^H-^15^N HSQC spectra as well as R_2_ relaxation rates (Figures 3, S1, and S4). Local backbone sampling even of the interacting F was not measurably modified upon interaction. The conformers of Nup153FG^PxFG^ that were subjected to Importinβ^N^ binding in the MD simulations were also devoid of large-scale conformational changes, and interactions were only observed between individual surface exposed residues of Nup153FG^PxFG^ and Importinβ^N^.

It appears therefore that globally, the FG-Nup maintains its conformational ensemble as shown by smFRET. This observation is sound, as IDPs frequently use motif binding to engage with their binding partners (Kragelj et al., 2015; Schneider et al., 2015; Tompa and Fuxreiter, 2008; Wright and Dyson, 2009). Our observation suggests an extraordinarily small motif (the side chain of F), which would be difficult to identify from large-scale bioinformatics approaches (Dinkel et al., 2014).

**Figure 7:**
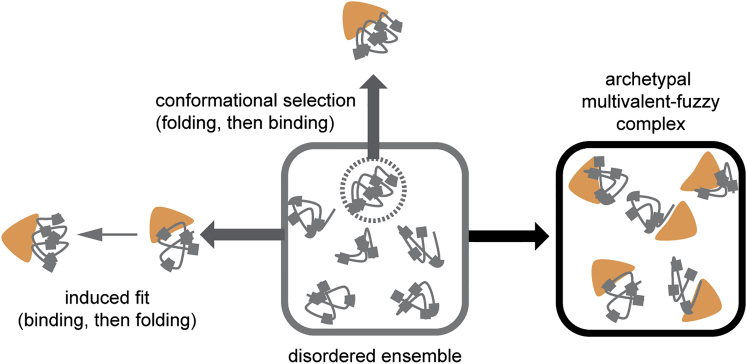
Binding Modes of IDPs to Folded Proteins Schematic representation of various models describing the binding of an IDP to its folded partner. In an induced-fit mechanism the IDP partially or completely folds upon interacting with its partner, potentially showing an intermediate encounter complex as in the fly-casting mechanism (Shoemaker et al., 2000). In a conformational selection mechanism, the folded protein selects one (or several) conformation(s) of the IDP that best fits its binding pocket. These models suggest a shift in the IDP’s conformational ensemble. For the Nup⋅NTR complex we observed formation of an “archetypal” fuzzy and multivalent complex, a binding mode that on a global scale does not require major energy or time investment for the Nup to transit from its free to the bound conformation. Note that multiple NTRs can bind one Nup and vice versa.

The observed binding mode appears distinct from other single motif binding interactions, as well as from mechanisms that involve global conformational transitions, such as folding upon binding (Csermely et al., 2010; Wright and Dyson, 2009) (Figure 7). The intrinsic flexibility of the Nup, the repeated occurrence and short length of the binding motif seem to create a highly reactive binding surface, which renders the individual FG-motifs prone to bind at any time without compromising the Nup’s inherent plasticity.

### Ultra Rapid Association of the Nup153FG⋅Importinβ Complex

The maximal association rate in the absence of electrostatic forces for a binary interaction system (in which all collisions are productive) can be approximated by the Einstein-Smoluchowski diffusion limit, which yields a theoretical k_on_ of ∼10^9^ M^−1^s^−1^ for the interaction of proteins of the size of Nup153FG and Importinβ.

Very high association rates have been observed previously in the presence of long-range electrostatic attractions (10^8^-10^10^ M^−1^s^−1^) for example for the barnase/barstar interaction (Spaar et al., 2006), as well as for small IDP complexes studied by NMR (Arai et al., 2012; Schneider et al., 2015). In the absence of electrostatic steering, this upper limit is typically never reached, as successful collisions require proper orientation of the binding partners. Consequently, most experimentally observed association rates at high salt concentrations fall into the regime of 10^4^–10^6^ M^−1^s^−1^ (Shammas et al., 2013, 2014).

Our ensemble FSF kinetics (for Nup153FG) and BD simulations (for Nup153FG^PxFG^) show a k_on_ of ∼10^9^ M^−1^s^−1^ (Figure 5) supporting the aforementioned idea of a strongly reactive binding surface. We specifically observe an influence of apolar desolvation energies in the BD simulation and electrostatics are not found to play a major role in association. This applies apparently to both, Nup153FG^PxFG^, which is uncharged and was tested in BD, as well as Nup153FG, which has several charges in the N-terminal regions (Figures 5D and S2). Even in the limiting case of electrostatic shielding we found complex formation to still have a remarkably fast k_on,FSF_ (Figures 5D, 5E and Table S2B).

While experimentally bridging the gap between our molecular-level description of the small binary Nup⋅NTR complex (160 kDa) in solution to the actual in vivo transport mechanisms (involving ∼120 MDa NPCs) is still a challenging quest, the sm transport experiments (Figure 6) underline that the initially unexpected kinetic findings for the Importinβ^DA^ mutant are in line with the finding in functional NPCs.

### Individual FG-Repeats Bind with Low Affinity and Act in Concert for Efficient Binding

According to ensemble titration fluorescence curves, we have observed an apparent local equilibrium constant (K_d,app_) between Nup153FG and Importinβ in the nanomolar regime (Figure 5C and Table S2). However, we report millimolar affinities per FG-motif from our NMR measurements within Nup153FG^PxFG^ (Figures 3 and S4), in line with a recent computational model (Tu et al., 2013). Our NMR studies further suggest that individual FG-motifs bind independently of each other, as the ^15^N R_2_ rates are similar to the values of the unbound Nup between the FG-repeats. Nevertheless, the sum of FG-motifs influences the effective binding strength of individual FGs to Importinβ, as can be seen by comparing the effective K_d_ for F1374 in the WT and the Nup153AG^PxAG, F1374^ mutant (Figure 3, S1, and S4).

While these estimates of K_d_ values (from NMR and ensemble fluorescence) were measured on different Nup constructs, they also report on two different properties: the binding of Importinβ to a larger region of Nup153FG (fluorescence anisotropy) and to a single FG-motif (NMR), and illustrate an important characteristic of the system, namely the importance of polyvalent interactions, which is exploited also by other transport receptors (Figure S4). While an individual FG-motif might be unlikely to be bound, the chances that at least one FG-motif within the Nup molecule is bound may remain high. This stabilizing effect of multivalency/polyvalency is well known, and is even used as a design principle in enhancing the affinity of ligand interactions with multi-site targets where ligands are connected in tandem via short linkers (Brabez et al., 2011; Kramer and Karpen, 1998). Stability enhancements achieved in such experiments can approach four-to-five orders of magnitude and are primarily due to substantial decreases in the global dissociation rate, i.e., in a multivalent system the molecules only separate as a result of a dissociation event if all other motifs are unbound.

To demonstrate generality of these three core findings, we performed additional smFRET, FCS (Figures S2 and S3), NMR (Figure S4), MD (Figure S5 and Movie S3), and FSF experiments (Figure S7 and Table S2C) on a variety of different Nups from human and yeast, including the most common motif in vertebrates (FxFG) and the crucial GLFG sequence in yeast, for a diverse set of NTRs (NTF2, TRN1, CRM1, Importinβ). All results are in close agreement, highlighting the universal nature of the observed mechanism.

Currently, several models are discussed on how a permeability barrier in the NPC can be built; among those are the selective phase, the brush, the reduction of dimensionality and the karyopherin centric model, etc., as well as mixtures of those (Eisele et al., 2013; Frey and Görlich, 2007; Jovanovic-Talisman et al., 2009; Lim et al., 2007; Lowe et al., 2015; Moussavi-Baygi et al., 2011; Peters, 2009; Wagner et al., 2015; Yamada et al., 2010). These models vary mainly over how FG-Nups are arranged and potentially interlinked inside the NPC to create a tight barrier. However, common to all these models is that the concentration of FG-repeats of about 50 mM creates a very crowded environment, which is roughly in line with stoichiometric measurements of Nups and the overall size of the central channel (Bui et al., 2013; Ori et al., 2013). Independently of the transport model assumed, mobility of an NTR inside the barrier is thus largely limited by the k_off_ and k_on_ of the interaction between FG-Nups and NTRs. This is also the case if FGs interact with FGs inside the barrier as proposed in the selective phase model (Frey and Görlich, 2007), as long as these interactions are highly dynamic and do not pose a substantial energetic barrier or rate-limiting step to be melted. That we do not observe obvious FG-FG interactions in our studies is thus not necessarily inconsistent with such a model.

If we were to naively consider the characteristic time for a single Nup and Importinβ to separate based on commonly measured fast k_on_ and affinities (e.g., K_d_ (Nup⋅NTR) ∼100 nM and k_on_ ∼10^6^ M^−1^s^−1^ → unbinding time (UT) ∼100 ms), it appears impossible that Importinβ could cross a 50 mM FG-filled pore within 5 ms. This is the previously described “transport paradox,” in which high specificity is somehow coupled with rapid transport (Bednenko et al., 2003; Ben-Efraim and Gerace, 2001; Tetenbaum-Novatt et al., 2012; Tu et al., 2013).

Our work (down to picosecond and atomic resolution) is largely compatible with the existing barrier models, as it addresses on a molecular mechanistic level how an NTR could rapidly pass through a dense barrier. Using a simple model of a bivalent system, we already expect an order of magnitude difference between the dissociation rate for an individual motif and that for the whole protein (Kramer and Karpen, 1998). We have also observed extremely rapid association rates (∼10^9^ M^−1^s^−1^) and in Supplemental Experimental Procedures (two toy models) we outline that if we consider a very rough estimate for the characteristic time for an individual motif unbinding event (UT∼1 μs) for full-length Nup153 (>24 valencies), it becomes clear that the Importinβ could “creep” through the dense FG-motif plug of the pore within the short transport time. Such movement is consistent with our (Figure 6) and other NTR diffusion studies through NPCs in intact cells and various model systems (Eisele et al., 2013; Frey and Görlich, 2007; Jovanovic-Talisman et al., 2009; Moussavi-Baygi et al., 2011; Schleicher et al., 2014; Tu et al., 2013; Wagner et al., 2015).

In this case, nature has achieved a combination of high specificity with fast interaction rates. This is based on many individual low-affinity motifs paired with a binding mode that requires relatively little energy or time investment for the Nup to transit between free and bound conformations, and provides a rationale for the fast, yet specific, nuclear transport. While rapid binding can in principle be realized between proteins of single binding elements (e.g., driven by strong electrostatics), the proofreading emanating from the multiplicity and rapid repetition of many such events is what contributes to specific transport.

We note that the transport paradox goes far beyond the relevance for the transport mechanism, since transient, but targeted interactions are central to the emerging view of highly dynamic protein (and other biomolecular) interaction networks. Furthermore, FG-repeats are also present in stress and P granules (Toretsky and Wright, 2014). It seems likely that such ultrafast binding mechanisms are also important for other biological recognition processes, where individual interaction motifs only make weak contributions, as e.g., in the recognition of glycans (Ziarek et al., 2013), or other very short linear motifs, like WG motifs in small RNA pathways (Chekulaeva et al., 2010), or binding of proteins to epigenetic marks, like many histone modifications.

In addition, ultrafast association is achieved by using the unique plasticity of multivalent disordered proteins, which is distinct from mechanisms where orientation specific binding is required for complex formation. This represents an additional biological advantage for IDPs in comparison to folded proteins, and might have further facilitated their enrichment in organisms of higher complexity.

## Experimental Procedures

### Expression and Purification of Importinβ, TRN1, NTF2, CRM1 and Nup153FG

The proteins were purified essentially as described in (Milles and Lemke, 2014) following routine column chromatography and then transferred into the respective measurement buffers. Labelling of Nup153FG (amino acids 875 to 1475 of the full length Nup153; numbering with respect to the full length protein as in ‘UniProt: P49790’) was performed using routine procedures to introduce Alexa488 as a donor and Alexa594 as an acceptor dye for smFRET experiments (and analog for other dyes), as described in (Milles and Lemke, 2011).

### NMR Studies of Nup153FG^PxFG^

Spectral assignments of ^13^C, ^15^N Nup153FG^PxFG^ were obtained from a set of BEST-TROSY-type triple resonance spectra: HNCO, intra-residue HN(CA)CO, HN(CO)CA, intra-residue HNCA, HN(COCA)CB, and intra-residue HN(CA)CB (Solyom et al., 2013). For the measurements of RDCs, ^13^C, ^15^N Nup153FG^PxFG^ was aligned in 12 mg/ml Pf1 phages yielding a D_2_O splitting of 2.16 Hz. RDCs were measured using BEST-type HNCO and HN(CO)CA experiments (Rasia et al., 2011). ^15^N relaxation dispersion was carried out at Nup153FG^PxFG^/Importinβ concentrations of 250 μM and 180 μM, respectively, applying CPMG frequencies between 25 and 1,000 Hz (Schneider et al., 2015). All experiments were performed in Na-phosphate buffer (pH 6), 150 mM NaCl, 2 mM DTT, 5 mM MgCl_2_, at 25°C and at a ^1^H frequency of 600 MHz if not noted otherwise.

The conformational space available to disordered Nup153FG^PxFG^ was sampled using the *Flexible-meccano* statistical coil description (Ozenne et al., 2012) and representative ensembles in agreement with experimental chemical shifts were selected using ASTEROIDS (Jensen et al., 2010) and the ensemble was subsequently cross-validated against experimental RDCs and SAXS.

### SmFRET Experiments

SmFRET measurements of dual labeled freely diffusing proteins were performed on a confocal geometry detecting donor and acceptor intensities (from which the FRET efficiency E_FRET_ is calculated) as well as fluorescence lifetimes (τ) on a custom built multiparameter setup as previously described (Milles and Lemke, 2011).

### Fluorescence Stopped-Flow Experiments

The association kinetics were monitored by following the fluorescence anisotropy change of Nup153FG labeled at the indicated position with Cy3B (see sequences in Supplemental Experimental Procedures) upon binding to different concentrations of NTRs, under pseudo-first order conditions. Anisotropy (r) was calculated from fluorescence intensities measured with polarizing filters in the parallel (‖) and perpendicular (⊥) position.

Each trace was obtained by averaging ≥30 traces and background fluorescence was then subtracted. The anisotropy traces where fit with a biexponential function to determine k_obs_. The different k_obs_ were plotted against the respective NTR concentrations and were linearly fit to obtain the association constant (k_on_) from the slope.

The used BioLogic (Grenoble, France) stopped-flow equipment permits automatic titration and repeated technical replicates, which typically yield a small standard deviation. We derived an experimental error of ∼20% in k_on_ measurements between different biological replicates. To be conservative, we thus do not show (the typically lower) standard deviations from technical replicates.

### Transport Experiments

Routine reconstitution of the nucleocytoplasmic transport machinery in permeabilized cells was used and fluorescence cargo (NLS-MBP-eGFP) was imaged on a confocal microscope (Leica, Mannheim) at the indicated time points.

For single molecule tracking of NTRs, the same assay was used, but Importinβ-Alexa488 at single molecule concentration was tracked with an acquisition time of 2ms on a previously described home built imaging microscope (Ori et al., 2013).

All data analyses for FSF, FCS, smFRET and tracking were performed with custom written routines in IgorPro (Wavemetrics, OR).

### MD and BD Simulations

The Nup153FG^PxFG^ fragment was modeled on the basis of its sequence that also included the exogenously inserted residues used for labeling of the fragment with fluorophores. For the binding simulations, Nup153FG^PxFG^ or Nup153^FGFxFG^ were randomly placed in a box of dimensions 15 × 15 × 15 nm^3^ together with the N-terminal segment of Importinβ^N^ (PDB: 1F59). Brownian Dynamics (BD) simulations were performed starting from the MD complex that showed a specific association between the partners, and resembled the crystallographic binding pose as reported by ref. (Bayliss et al., 2000).

## Author Contributions

S.M., D.M., I.V.A., designed and performed experiments, analyzed data and co-wrote the manuscript. M.R.J., N.B., C.K., S.T., J.C. provided additional reagents and analysis tools. S.L.S. designed experiments and analysis methods and co-wrote the manuscript. M.B., F.G. and E.A.L. conceived the project and co-wrote the manuscript.

## Accession Numbers

The accession number for the data reported in this paper is Protein Ensemble Database (PED): 2AAE.

## Supplemental Information

Document S1. Supplemental Experimental Procedures and Table S1

Table S2. Stopped-flow Measured Data, Related to Figure 5 (A) Stopped-flow measurement results of Nup153FG●Importinβ. (B) Results from the ionic strength dependence stopped-flow measurements of Nup153FG●Importinβ. (C) Results from stopped-flow measurements of different FG-rich regions in Nup153FG with Importinβ, NTF2 and TRN1, corresponding to Figure S7. (D) Comparison of values between FSF and BD. (E) Reaction criteria used for determining the occurrence of encounter complexes in BD simulations.

Movie S1. Conformational Ensemble of Nup153FG^PxFG^, Simulated Using AMBER99-sb∗-ILDN Force Field, Related to Figure 4 The movie shows one of the performed replicas (15 replicas each 150 ns long have been performed per force field). Protein backbone is represented as a red ribbon whereas the FG-repeats along the sequence are represented as cyan beads. On the left, simulated time is represented using a time bar and measured in ps.

Movie S2. Binding of Nup153FG^PxFG^ to the Major Binding Site of Importinβ^N^, Related to Figure 4 The movie shows one of the performed simulations. Nup153FG^PxFG^ is represented as described in Movie S1. Importinβ^N^ colored gray. The region of the protein including the major binding pocket of Nups is colored orange. On the left, simulated time is represented using a time bar and measured in ps.

Movie S3. Binding of Nup153FG^FxFG^ to the Major Binding Site of Importinβ^N^, Related to Figure 4 The movie shows one of the performed simulations. Nup153FG^FxFG^ is represented as described in Movie S1. Importinβ^N^’s surface is shown and colored gray. The region of the protein including the major binding pocket of Nups is colored orange. On the left, simulated time is represented using a time bar and measured in ps.

Document S2. Article plus Supplemental Information
