# Supplementary material for: Plasticity of an Ultrafast Interaction between Nucleoporins and Nuclear Transport Receptors
Source: Cell. 2015 Oct 22;163(3):734–45. doi: 10.1016/j.cell.2015.09.047 (PMC4622936; doi:10.1016/j.cell.2015.09.047)
Supplement: Document S1. Supplemental Experimental Procedures and Table S1 [file mmc1.pdf]

Cell
Supplemental Information

Plasticity of an Ultrafast Interaction between Nucleoporins and Nuclear Transport Receptors

Sigrid Milles, Davide Mercadante, Iker Valle Aramburu, Malene Ringkjøbing Jensen, Niccolò Banterle, Christine Koehler, Swati Tyagi, Jane Clarke, Sarah L. Shammas, Martin Blackledge, Frauke Gräter, and Edward A. Lemke

Supplemental Information

Supplemental Experimental Procedures

Sequences of used constructs

Nup153FG (875-1475)

10 20 30 40 50 60
SAKPGTKCGF KGFDTSSSSS NSAASSSFKF GVSSSSSGPS QTLTSTGNFK FGDQGGFKIG
70 80 90 100 110 120
VSSDSGSINP MSEGFKFSKP IGDFKFGVSS ESKPEEVKKD SKNDNFKFGL SSGLSNPVSL
130 140 150 160 170 180
TPFQFGVSNL GQEEKKEELP KSSSAGFSFG TGVINSTPAP ANTIVTSENK SSFNLGTIET
190 200 210 220 230 240
KSASVAPFTA KTSEAKKEEM PATKGGFSFG NVEPASLPSA SVFVLGRTEE KQQEPVTSTS
250 260 270 280 290 300
LVFGKKADNE EPKAQPVFSF GNSEQTKDEN SSKSTFSFSM TKPSEKESEQ PAKATFAFGA
310 320 330 340 350 360
QTSTTADQGA AKPVFSFLNN SSSSSSTPAT SAGGGIFGSS TSSSNPPVAT FVFGQSSNPV
370 380 390 400 410 420
SSSAFGNTAE SSTSQSLLFS QDSKLATTSS TGTAVTPFVF GPGASSNNTT TSGFGFGATT
430 440 450 460 470 480
TSSSAGSSFV FGTGPSAPSA SPAFGANQTP TFGQSQGASQ PNPPGFGSIS SSTALFPTGS
490 500 510 520 530 540
QPAPPTFGTV SSSSQPPVFG QQPSQSAFGS GTTPNCSSAF QFGSSTTNFN FTNNSPSGVF
550 560 570 580 590 600
TFGANSSTPA ASAQPSGSGG FPFNQSPAAF TVGSNGKNVF SSSGTSFSGR KIKTAVRRRK

F (total) = 60 # FxFG = 14 # PxFG= 5 # FG= 4 # SAFG= 2

Thick underlined sequence indicates Nup153FG^{PxFG}

Nup153FG^{PxFG} (1313-1390) fragment

(G/G/A)(C/A/C)PSASPAFG ANQTPTFGQS QGASQPNPPG FGSISSSTAL FPTGSQPAPP
TFGTVSSSSQ PPVFGQQPSQ SAFGSGTTPN (C/C/AcF)A

F (total) = 7 # FxFG = 0 # PxFG= 5 # FG= 0 # SAFG= 1

The constructs used for the various experiments (NMR/FSF/smFRET) differ as indicated in brackets.

Thin underlined sequence indicates Nup153FG^{FxFG}

Nup153FG^{FxFG} (883-993) fragment

GCGF KGFDTSSSSS NSAASSSFKF GVSSSSSGPS QTLTSTGNFK FGDQGGFKIG VSSDSGSINP
MSEGFKFSKP IGDFKFGVSS ESKPEEVKKD SKNDNFKFGL SSGLSNPVA

F (total) = 13 # FxFG = 4 # PxFG= 0 # FG= 0 # SAFG= 0

C labeled Cys

Expression and purification of Nup153FG constructs for smFRET and stopped flow experiments. Unless specified differently, all reagents were purchased from Sigma Aldrich at molecular biology or cell culture grade.

The codon-optimized Nup153FG^{PxFG} (amino acids 1312 to 1391 of the full length Nup153; numbering with respect to the full length protein as in "UniProt: P49790") was cloned into a pTXB3 vector as a 6His Nup153FG^{PxFG} intein chitin binding domain (CBD) construct with a TEV (Tobacco Etch Virus) cleavage site after the His-tag. A TAG stop-codon for Amber suppression and a cysteine were introduced at positions 1391 and 1312, respectively. The protein was recombinantly expressed in *Escherichia coli* (*E. coli*) BL21(AI) (Invitrogen, Carlsbad, CA) harboring the pEvol AcF plasmid for Amber suppression and genetic code expansion in Terrific Broth (TB) medium at 37°C and in the presence of 1 mM p-acetylphenylalanine (AcF) (Milles and Lemke, 2011). Expression was induced at OD_{600} = 1 with 0.02 % arabinose and 1 mM IPTG, and cells were harvested after 4 hours. The protein was purified using standard Ni and Chitin affinity purifications under mild denaturing conditions (phosphate buffered saline (PBS), pH 8, 150 mM NaCl, 2 M urea), intein and 6His-tags were simultaneously cleaved in 20 mM Tris, pH 8 with 100 mM β-mercaptoethanol (BME) and 1 mg of TEV protease per 2 liter expression. Nup153FG^{PxFG} was separated from the His-tagged TEV protease by Ni affinity purification. Fluorescence labeling (maleimide and oxime ligation with Alexa594-maleimide and Alexa488-hydroxylamine (Invitrogen, Carlsbad, CA)) was done as previously described (Milles and Lemke, 2011; Milles et al., 2012), and the protein was separated from free dye by size exclusion chromatography. Nup153FG^{PxFG} was transferred into PBS pH 7.4, 150 mM NaCl, 4 M guanidinium hydrochloride for long-term storage at -80°C. For smFRET measurements, Nup153FG was transferred into smFRET buffer (PBS pH 7.4, 150 mM NaCl, 2 mM DTT, 1 mM $Mg(CH_3COO)_2$).

For fluorescence stopped-flow (FSF) experiments, single cysteine mutant of the full length Nup153FG were labelled analogously with Cy3B maleimide (GE Lifesciences, Pennsylvania).

The full-length Nup153FG was essentially the same as in (Milles and Lemke, 2011). A single cysteine and TAG stop codon for Amber suppression were introduced by site-directed mutagenesis (position 1312 and 1391 respectively).

The mutants FxFG rich region I Nup153FG$^{FxFG(I)}$ (S994TAG,S883C) and FxFG rich region II Nup153FG$^{FxFG(II)}$ (S990C,S1049TAG) were purified and labeled as described above. The same purification was performed with the single cysteine labeled Nup153FG mutants (S1391C and S883C).

Nup153AG was expressed from a pBAD vector in a Nup153AG intein CBD 12His construct. Labeling sites were inserted as for Nup153FG. Expression and purification procedures followed were the same as in (Milles et al., 2013) and labeling of the purified protein was same as for Nup153FG.

Expression and purification of yNup49FG for smFRET. The yNup49FG (amino acids 1-260, numbering with respect to the full length protein "UniProt: Q02199") was expressed from a

pBAD-6His-Intein vector. This construct contains a C at residue position 250. The mutation A191TAG was introduced in addition to permit FRET labelling as described above for the Nup153FG proteins. Purification was performed using standard Ni affinity purification procedure with mild denaturing conditions (phosphate buffered saline (PBS), pH 8, 150 mM NaCl, 2 M urea). 6His-tag was cleaved by incubating the sample with 100 mM β-mercaptoethanol (BME) overnight at room temperature. After buffer exchange a second Ni affinity purification was performed and after labeling the cleaved yNup49FG was run over size exclusion chromatography.

Expression and purification of Nup153FG for NMR experiments. The same $Nup153FG^{PxFG}$ construct as for fluorescence measurements, but with a Cys instead of AcF was transformed in *E.coli* BL21 (DE3) and grown in Lysogeny broth (LB) medium at 37°C at an OD_{600} of 0.8. The culture was harvested and transferred into M9-AC medium containing 1 g/l $^{15}NH_4Cl$ and 2 g/l glucose (^{13}C labeled if needed). Growth was continued for 1 h at 20°C until expression was induced by addition of 1 mM IPTG for 12 to 14 h. Purification of $Nup153FG^{PxFG}$ was done essentially as for fluorescence experiments in 50 mM Tris pH 8, 150 mM NaCl. As a last step, $Nup153FG^{PxFG}$ was transferred into NMR buffer (50 mM Na-phosphate pH 6, 150 mM NaCl, 5 mM $MgCl_2$, 2 mM dithiothreitol (DTT)) by size exclusion chromatography.
^{15}N labeled $Nup153AG^{PxAG,\ F1374}$ was cloned the same way as $Nup153FG^{PxFG}$, but all F except F1374 were replaced by A. Expression and purification were analogous to $Nup153FG^{PxFG}$.

Expression and purification of Importinβ. Importinβ was expressed and purified essentially as described (Milles and Lemke, 2014). Briefly, an intein-6His construct in a pTXB3 vector was transformed in *E.coli* BL21 DE3(AI). Cultures were grown in TB or LB medium at 37°C until OD_{600} = 0.6, induced with 0.02 % arabinose and 1 mM IPTG, and growth was continued at 30°C for 4 hours or 20°C for 12 to 14 hours. Purification was done following standard Ni affinity protocols in 50 mM Tris pH 7.0, 650 mM NaCl, 5 mM $MgCl_2$. 0.2 mM TCEP (tris(2-carboxyethyl)phosphine). Intein cleavage was done overnight at room temperature using 100 mM BME, and the tag was removed by an additional Ni-affinity step. The protein was concentrated and submitted to size-exclusion chromatography. Importinβ was then transferred into NMR or smFRET buffer.

Expression and purification of Transportin1. Transportin1 (TRN1) was expressed in *E.coli* XL10 Gold cells transformed with pQE60-Transportin1-6His. Protein expression was induced at OD_{600}=0.6 with 1mM IPTG and the cultures were grown for 6 hours at 30 ˚C. Harvested cells were lysed in 50 mM Tris pH 8, 500 mM NaCl, 5 mM $MgCl_2$, 1 mM PMSF and 0.2 mM TCEP. The lysate was incubated with Ni-beads after centrifugation and a 1.5 M NaCl wash was performed before elution. The eluted protein was then run over size exclusion chromatography.

Expression and purification of NTF2. NTF2 was expressed in *E.coli* BL21 (DE3) transformed

with a pTXB3-NTF2-Intein-6His plasmid. Expression was induced with 1 mM IPTG at OD_{600}=0.6 and cells were incubated overnight at 34 ˚C. Harvested cells were lysed with 50 mM Tris pH 8, 500 mM NaCl, 5 mM $MgCl_2$, 5 mM imidazol, 1 mM PMSF and 0.2 mM TCEP. Then the clear lysate was incubated with Ni-beads. The elution from the Ni-beads was incubated with 100 mM BME overnight at room temperature, in the presence of Complete EDTA (Roche) free protease inhibitors. After buffer exchange the sample was incubated again on Ni-beads and the untagged NTF2 was run over size exclusion chromatography.

Expression and purification of CRM1. CRM1 was expressed on *E.coli* TG1 cells transformed with a pQE60-CRM1-Intein-12His vector. Protein expression was performed in TB medium. Induction was done at OD_{600}=0.8 with 1 mM IPTG and the cultures were incubated at 18 ˚C overnight. The harvested cells were lysed with 50 mM Tris pH 8, 650 mM NaCl, 5 mM imidazol, 1 mM PMSF and 0.2 mM TCEP. The clear lysate after centrifugation was then incubated with Ni beads following standard protocols. The elution from the Ni-beads was incubated with 100 mM BME overnight at room temperature to cleave the intein-12His-tag in the presence of Complete EDTA free protease inhibitors. Then the sample was buffer exchanged to perform a second incubation with Ni-beads. The obtained cleaved protein was then run over size exclusion chromatography.

Spectral assignment of Nup153FG^{PxFG} and RDC measurements. Spectral assignments of ^{13}C, ^{15}N Nup153FG^{PxFG} were obtained from a set of BEST-TROSY-type triple resonance spectra: HNCO, intra-residue HN(CA)CO, HN(CO)CA, intra-residue HNCA, HN(COCA)CB, and intra-residue HN(CA)CB (Solyom et al., 2013). The spectra were processed with NMRPipe (Delaglio et al., 1995) and automatic assignment was done with the program MARS (Jung and Zweckstetter, 2004), followed by manual verification. Secondary chemical shifts were calculated using the random coil values from refDB (Zhang et al., 2003).
For the measurements of RDCs, ^{13}C, ^{15}N Nup153FG^{PxFG} was aligned in Pf1 phages to yield a D_2O splitting of 2.16 Hz. RDCs were measured using BEST-type HNCO and HN(CO)CA experiments allowing for spin-coupling measurements in the ^{13}C dimension (Rasia et al., 2011). All experiments were performed in NMR buffer, at 25°C and at a ^{1}H frequency of 600 MHz.

Ensemble description of Nup153FG^{PxFG}. A statistical coil ensemble of Nup153FG^{PxFG} comprising 10,000 structures was generated using Flexible-Meccano. The genetic algorithm ASTEROIDS was used to select five times 200 conformations that best described the experimentally obtained chemical shifts (Jensen et al., 2010). Based on the ϕ and ψ angles of the selected conformers, a new ensemble of 8,500 conformers was generated, supplemented with 1500 structures from the initial ensemble, and subjected to another round of ASTEROIDS selection. This iteration step was repeated four times as described previously (Ozenne et al., 2012) until convergence was achieved with respect to the experimental chemical shifts.
Based on the selected ensembles (5 times 200 structures) from the fourth iteration, 10,000

conformers were again generated using Flexible-Meccano, from which ${}^1D_{N-NH}$, ${}^1D_{C\alpha-H\alpha}$ RDCs were calculated and compared to the experimentally obtained values.
Ensemble averaged chemical shifts were obtained using SPARTA (Shen and Bax, 2007), RDCs were calculated using PALES (Zweckstetter et al., 2004) and SAXS curves were calculated using CRYSOL (Svergun et al., 1995).

^{15}N relaxation and relaxation dispersion of Nup153FG^{PxFG} and Nup153AG$^{PxAG, F1374}$. ^{15}N R_1 and $R_{1\rho}$ experiments and $\{^1H\}$-^{15}N heteronuclear Overhauser effetcs (nOe) were carried out at a ^{1}H frequency of 600 MHz and at a temperature of 25°C. The ^{15}N R_1 relaxation rates were obtained by sampling the decay of magnetisation at 0, 0.08, 0.19, 0.38, 0.57, 0.76, 0.99, 1.44, and 1.71 s. The time point for a delay time of 0.57 s was repeated for error estimation. $R_{1\rho}$ relaxation rates were determined using delay times of 0.001, 0.02, 0.05, 0.08, 0.1, 0.15, 0.2, and 0.25 s, with a repetition at a delay time of 0.25 s for the interaction with Importinβ and the nucleoporin alone. Interactions with TRN1 and NTF2 were measured with time delays 0, 0.076, 0.304, 0.57, 0.874, 1.52 s with a repetition at delay time 0.57 s for R_1 and 0.001, 0.02, 0.05, 0.1, 0.17, and 0.25 s with a repetition at delay time 0.05 s for $R_{1\rho}$. The spin-lock field was 1500 Hz. R2 was calculated based on R1 and R1ρ according to ref. (Akke and Palmer, 1996).

$$R_{1\rho} = R_1 \cos^2(\theta) + R_2 \sin^2(\theta)$$

with $\theta = \arctan(\frac{\gamma_N B_1}{\Delta\upsilon \cdot 2\pi})$.

^{15}N relaxation dispersion was carried out at a Nup153FG^{PxFG} concentration of 250 μM and an Importinβ concentration of 180 μM (Hansen et al., 2008). 22 points, including one duplicate, were recorded at CPMG frequencies between 25 and 1,000 Hz using constant-time relaxation delay of 40 ms.

Calculation of residue-specific apparent K_d values from ^{15}N relaxation. R_2 from every F in the Nup153FG^{PxFG} and Nup153AG$^{PxAG, F1374}$ sequence was extracted for all the experiments at different Importinβ concentrations. The observed R_2 can be described as the weighted average between R_2 in the bound (R_2^{NI}) and unbound (R_2^N) form of Nup153FG according to

$$R_2 = c \cdot R_2^{NI} + (1-c) \cdot R_2^N$$

c describes the concentration ratio of the complex with respect to the total nucleoporin concentration as follows:

$$c = \frac{[NI]}{N_{tot}}$$

This ratio contains the dissociation coefficient (K_d) according to $K_d = \frac{[N]\cdot[I]}{[NI]}$, and

$[I] = [I_{tot}] - [NI]$, resulting in $[NI] = \frac{[N]\cdot[I_{tot}]}{K_d + [N]}$.

With the rotation time of Importinβ as measured by fluorescence correlation spectroscopy (Milles and Lemke, 2014), we can calculate the expected R_2^I for Importinβ assuming isotropic rotation as a spherical molecule (48 s^{-1} at 600MHz if only dipolar relaxation is considered). We can then further assume that the local correlation time (τ_c) of the FG repeats of Nup153FG when bound to Importinβ is equal to the correlation time of isolated Importinβ ($R_2^{NI} \approx R_2^I$).
Under the assumption of [N] = [N_{tot}], i.e. that only a small fraction of the nucleoporin is bound, we obtain the following expression for the observed R_2:

$$R_2 = \frac{[I_{tot}]}{(K_d + [N_{tot}])}(R_2^I - R_2^N) + R_2^N .$$

When we calculate the ratio of bound nucleoporin compared to the total nucleoporin concentration according to

$$\frac{[NI]}{[N_{tot}]} = c = 1 - \frac{R_2 - R_2^N}{R_2^N - R_2^{NI}}$$

at the highest Importinβ concentration employed and for the largest R_2 measured within an FG repeat, we obtain a fraction of bound Nup153 of lower than 17%.
The observed R_2 rates were then plotted against Importinβ concentration (Nup concentration remained constant at 250 μM), and a linear fit was obtained from these plots for every F, and the K_d was extracted from the slope.
Note that this calculation implicitly assumes that all F of Nup153FG^{PxFG} are free to bind even if one (or more) F of the same molecule are already bound to Importinβ, and that binding of Importinβ does not reduce the effectively available Importinβ concentration. Several FG binding sites are known on Importinβ (Bayliss et al., 2000; Bednenko et al., 2003; Isgro and Schulten, 2005; Otsuka et al., 2008), and we have shown that multiple Importinβ molecules can likely bind the full Nup153FG at the same time (Milles and Lemke, 2014).
The same protocol was applied to extract residue specific K_d values for TRN1 binding. Since Importinβ and TRN1 have approximately the same size and similar structures, we assumed the same rotation time as for Importinβ. Nup153FG^{PxFG} concentration remained constant at 250 μM and the TRN1/Nup153FG^{PxFG} molar ratio was varied according to 0.61, 0.33, and 0.17. While the same relaxation experiments were performed with NTF2, no experimental rotational correlation time of the complex was available and since NTF2 is very small compared to Importinβ and TRN1, the assumption of equal rotation in the bound and unbound form likely does not hold. Site-specific K_d values were therefore extracted from chemical shift changes.

Calculation of residue-specific apparent K_d values from chemical shift changes. ^{1}H-^{15}N HSQC spectra were measured at a ^{1}H frequency of 850 MHz and 25°C with a Nup153FG^{PxFG} concentration of 80 μM and varying NTF2 concentrations (6-, 12-, 18-, and 24-fold excess of NTF2). 36- and 48-fold excess of NTF2 was measured at a Nup concentration of 60 and 40 μM respectively, as only lowering the Nup concentration allowed these NTF2/Nup ratios. Chemical shifts for all F (except F1327, which was excluded due to spectral overlap), were extracted and

absolute chemical shift changes were calculated according to $\Delta = \sqrt{(\delta^{15}N)^2 + (\delta^{1}H \cdot 6.5)^2}$, with $\delta^{15}N$ and $\delta^{1}H$ the ^{15}N and ^{1}H chemical shift changes with respect to the unbound Nup153FG^{PxFG}. Δ was then plotted against the molar ratio between NTF2 and Nup153FG^{PxFG} and fit with a simple binding model under the assumption of excess NTF2 ($\Delta = \frac{\Delta_{max} \cdot B}{K_d + B}; B = \frac{NTF2}{Nup}$). Errors were extracted from the fit.

SmFRET experiments and protein diffusion. SmFRET measurements and the experimental setup for the measurements have previously been described (Milles and Lemke, 2011; Milles et al., 2012). The different FRET labelled Nup153FG constructs were provided at a concentration of ~ 50 pM and Importinβ, TRN1, NTF2 and CRM1 were added at 1 μM concentration. In the case of yeast Nup49 (aa 1-260) smFRET measurements were performed with 10 μM NTR concentration. Single molecule fluorescence lifetimes were calculated using a maximum likelihood estimator (Schaffer et al., 1999). E_{FRET} values were corrected for donor leakage into the acceptor channel, direct excitation of the acceptor by the 483 nm laser and intensity differences in the green (donor, I_{Do})/red (acceptor, I_{Ac}) channels due to differences in detection and fluorescence quantum yields (γ) (Ferreon et al., 2009):

$$E_{FRET} = \frac{I_{Ac}}{I_{Ac} + \gamma \cdot I_{Do}}$$

Measurements were acquired with interleaved donor and acceptor excitation at a pulse rate of 27 MHz per color. For analysis of ensemble averaged lifetime histograms, E_{FRET} versus stoichiometry (S) histograms with

$$S = \frac{I_{Do} + I_{Ac}}{I_{Do} + I_{Ac} + I_{Ac}^{PIE}},$$

With I_{Ac}^{PIE} being the acceptor fluorescence upon acceptor excitation, were generated. Fluorescence lifetime histograms were built from either all photons resulting from bursts that contained double labeled molecules, or all photons resulting from donor only containing bursts. Accumulated fluorescence lifetimes from single molecule events were fit with a simple mono-exponential decay convolved with the impulse response function.

Fluorescence correlation spectroscopy (FCS) experiments were performed on a custom built confocal setup. Linearly polarized laser light was used to excite the samples. Detection of fluorescence signal was performed in parallel and perpendicular polarization directions and then the correlation curve was obtained by cross correlation. The curves were normalized for comparison purposes. The titration experiments with different Importinβ concentrations were performed with Cy3B single labeled proteins. FCS of double labeled proteins was measured in order to test binding of different NTRs to the proteins used in smFRET experiments.

Fluorescence stopped-flow (FSF) experiments. The association kinetics were monitored by following the anisotropy change of Nup153FG^{S1391C} labeled with Cy3B at the position 1391C

(near the PxFG region) and of Nup153FG^{S883C} labeled with Cy3B at the position 883C (FxFG motif enriched region, see sequences) upon binding to different NTRs at different concentrations, under pseudo-first order conditions, using stopped-flow spectroscopy (SFM-3000, Bio-logic) with the uFC-08 micro-cuvette accessory. Excitation was performed with a custom polarized LASER excitation source 532nm and polarized emission was detected using emission filters with 538-642nm bandwidth. Fluorescence intensities were measured with polarizing filters in parallel (∥) and perpendicular (⊥) position. The anisotropy (r) was calculated following:

$$r = \frac{I_{\parallel} + G \cdot I_{\perp}}{I_{\parallel} + 2G \cdot I_{\perp}}$$

Measurements were performed using the automatic mixing function (concentration dependent study) provided by the software (Bio-Kine32 V.4.72). 20 nM Nup153FG-Cy3B were mixed with the different NTRs at 200-400 nM in 50 nM steps. All the measurements were performed at 20 ˚C. Each trace was obtained by averaging 30 traces and background fluorescence subtracted. The anisotropy traces where fit to:

$$f(t) = A1 \cdot (1 - e^{-k1_{obs} \cdot t}) + A2 \cdot (1 - e^{-k2_{obs} \cdot t}) + c$$

The different k_{obs} were plotted against the respective Importinβ concentrations and were linearly fit to obtain the corresponding association constant (k_{on}). The estimation of the equilibrium dissociation constant (K_d) was obtained from the final anisotropy value of measurements by fitting it to:

$$r = \frac{[\mathrm{Imp}\beta]}{[\mathrm{Imp}\beta] + K_d} \cdot (r_B - r_F) + r_F$$

where r_B and r_F correspond to the anisotropy of free and bound Nup153FG-Cy3B and [Impβ] is the Importinβ concentration.

Effect of electrostatics as assessed by FSF experiments. The stopped-flow measurements at different ionic strength were performed at 20 mM MOPS pH 7.4 with various NaCl concentrations at 20˚C. MOPS buffer was used because it buffers in the same range as PBS but enables to lower the ionic strength. In **Figure 5**, The $k_{on,ultrafast}$ obtained from the fit of the association experiment between Nup153FG and Importinβ WT were plotted against the corresponding ionic strength. Points which were out of a three standard deviation confidence band were treated as outliers. The basal association rate constant was obtained by fitting the data to a Debye-Huckel-like approximation as used in ref.(Shammas et al., 2014).

$$\ln(k_{on}) = \ln(k_{on,basal}) + \frac{AB}{BR} \cdot \frac{I^{-0.5}}{BR + I^{-0.5}}$$

where I is the ionic strength of the buffer and AB and BR are used as free fitting parameters.

Error in FSF experiments. The BioLogic stopped flow equipment permits automatic titration and repeated technical replicates, which typically yield a small standard deviation. However, for k_{on} measurements of such complex systems, several experimental parameters are important,

especially the exact concentration and purity of proteins. We thus performed over several months repeatedly the same measurements from different protein expression batches. From this, we derived an experimental error of ~20% in k_{on} measurements between different replicates. To be conservative, we thus do not show (the typically lower) standard deviations from technical replicates.

Estimation of the theoretical diffusion-limited rate constant. The diffusion-limited binding rate constant for bimolecular reactions can be calculated by substituting the Stokes-Einstein relations into the Smoluchowski diffusion-limited equation:

$$k_{on} = 4\pi DR = N_A \frac{2k_B T}{3\eta}\left(\frac{D_A}{D_B} + \frac{D_B}{D_A} + 2\right)$$

Where η is the viscosity of the solution, k_B the Boltzmann constant, T the temperature, D_A and D_B correspond with the diffusion coefficient of Importinβ and Nup153FG. FCS was performed to determine the diffusion coefficients (D) of Nup153FG 1391C, Nup153FG^{PxFG} and Importinβ labeled with Atto655. The D obtained were $3.83 \cdot 10^{-5}$, $1.42 \cdot 10^{-5}$ and $2.34 \cdot 10^{-5}$ $cm^2 s^{-1}$ respectively. The estimated diffusion-limited association rate constant for Importinβ and Nup153FG 1391C was of $6.8 \cdot 10^9$ $M^{-1}s^{-1}$ and for Importinβ and Nup153FG^{PXFG} of $6.81 \cdot 10^9$ $M^{-1}s^{-1}$. These values are within the accepted range of 10^9-10^{10} $M^{-1}s^{-1}$ for diffusion-limited reactions. In BD simulations, a truncated Importinβ^N was used, which would have a higher diffusion coefficient and thus also a slightly higher theoretical k_{on}. Overall, the changes in k_{on} due to different MW of the proteins in BD and FSF experiments are low (~ 6 fold in molecular weight), and were thus not corrected for (see experimental error discussion for FSF experiments).

Limitations of SFS measurements. Stopped flow experiments of Nup153FG^{PxFG} with Importinβ did not show any appreciable signal change in anisotropy (**Figure S7)**, due to the much lower affinity of this short construct to the NTR. Thus, we measured the full-length Nup153FG, which has a much higher affinity and yielded appreciable signal changes. The anisotropy changes are for most conditions best fit by a biexponential decay. One rate lies typically in the order of $k_{on,ultrafast} \sim 10^9$ $M^{-1}s^{-1}$, while the second one is around one order of magnitude lower $k_{on,\ fast} \sim 10^8$ $M^{-1}s^{-1}$. The amplitude of the second component decreases with increasing Importinβ concentration, so that the first component is the dominating species.

It is important to note that we monitor the interaction of two large highly multivalent proteins; Nup153 has 25 FG repeats, and Importinβ has been suggested to have between 4- 16 FG binding sites (Isgro and Schulten, 2005; Otsuka et al., 2008). Previous studies also suggested that Importinβ binds preferentially to different regions in the Nup153FG, and that we can expect up to 8 Importinβ molecules to bind to a single Nup153FG (Milles and Lemke, 2014). The multiple components most likely indicate primary binding and then slower secondary binding events of Importinβ molecules, which would be perfectly in line with observations made for multiple Importinβ binding to immobilized layers of Nup153 by (Schoch et al., 2012; Wagner et al., 2015). While we cannot pinpoint the exact origin of the two components, both are very fast, and

support all conclusions drawn in the paper. Crucially we are interested in how fast the first F binds, and as we probe entire segments and not just single F's in the anisotropy measurements (Milles and Lemke, 2014) the highest observed rate still defines a lower limit for this.

***k_{off}* estimates.** Note that the error for the intercept of the SFS measurement shown in **Figure 5** is very high. In principle from this a $k_{off,SFS}$ can be determined, and we report those also in **Table S2A**. Another way to get an estimate of k_{off} is via $K_{d,app}= k_{off,global}/k_{on,SFS}$. Due to the nature of our anisotropy measurement, we only detect local binding near the dye, and amplitude and rise of the concentration dependent binding curve can be affected by different multivalent effects (see (Milles and Lemke, 2014) for a detailed discussion). However, an apparent $K_{d,app}$ can still be extracted, which is useful for relative comparisons, and can be used to calculate a $k_{off,global}$. $k_{off,global}$ and $k_{off,SFS}$ turned out to be on the same order of magnitude for Importinβ measurements. Since the error in $k_{off,global}$ is much lower it is the preferred value used in the text.

Toy models to estimate site specific $k_{off,single}$ in a multivalent system. The polyvalency/multivalency within the system we have described is extensive and therefore problematic to model. However to illustrate its importance in determining dissociation rates for Nup153 molecules we consider two separate toy models.

We first consider as a limiting case a simple bivalent system. The two molecules are only allowed to separate if the neighbouring motif is also unbound when the dissociation event occurs. The probability of this is related to the affinity of that site and the effective concentration (given that its neighbor is bound nearby). According to Kramer and Karpen this reduces the overall dissociation rate constant according to (Kramer and Karpen, 1998):

$$k_{off,\text{dimer}} = k_{off,\text{single}} \frac{2K_d}{K_d + C_{eff}}$$

where K_d is the equilibrium constant for an individual site and C_{eff} is the local concentration of binding sites. As the full length Nup153FG domain has 25 FG binding sites (and 60 F in total), one can expect that in such a system the $k_{off,single}$ for each individual site would be related to the overall (measured) dissociation rate as follows:

$$k_{off,\text{single}} >> k_{off,globalSFS} (\frac{K_d + C_{eff,SFS}}{2K_d})$$

The average measured $K_{d,NMR}$ across all sites in Nup153FG^{PxFG} as measured by NMR (**Figure 3**) is ~ 2mM, and we use this figure to represent K_d. We can then combine this value with an estimate of the effective concentration of sites to provide a lower bound for $k_{off,single}$. Based upon previously reported dimensions of intrinsically disordered proteins (Bernado and Blackledge, 2009) the effective concentrations of FG motifs and F motifs within each Nup153 molecule are approximately 40 mM and 90 mM respectively. Using these figures we conclude that $k_{off,single}$ 10^3 s^{-1}.

Interestingly we note that since the stoichiometry and dimension of the NPC are known, the

concentration of FG repeats within the NPC has already been estimated to be 50 mM (Bui et al., 2013; Frey and Gorlich, 2007; Ori et al., 2013).

We then obtain an estimate of the dissociation rate by taking an entirely separate approach (toy model 2) and considering the association rates. For the simplest case, where only a single F motif is contained within the shorter Nup construct, the interaction is bimolecular, and we can apply $k_{off}(F1374) = k_{on}(F1374) \bullet K_d(F1374)$ to create an estimate of $k_{off,single}$. The equilibrium constant is known from our NMR measurements, $K_{d,NMR}(F1374)$= 8 mM (Figure 3). For practical reasons it is not possible for us to determine $k_{on}(F1374)$ experimentally, however we form an approximation for informative purposes. For diffusion-limited reactions where the entire surface is considered equally reactive the protein size is expected to make very limited difference to the reaction rate as a result of counteracting effects of diffusion rates and target area so we approximate $k_{on,global\ PxFG} \sim k_{on,global\ SFS}$. However for Nup153AG$^{PxAG,\ F1374}$ we anticipate that the achieved association rate will be lower than $k_{on,global,PxFG}$ (and therefore $k_{on,global\ SFS}$) because mutating all but one of the 8 identified binding sites leads to a roughly 8-fold lower proportionate "reactive" surface of the protein and so fewer collisions will be correctly aligned. To a first approximation therefore $k_{on,NMR}(F1374) = k_{on(global,SFS)}/8$. This leads to an estimate of $k_{off,single}$ as $k_{off}(F1374) = k_{on}(F1374) \bullet K_d(F1374) \sim 10^6\ s^{-1}$, corresponding to an unbinding time (UT) of ~1 µs.

We stress that both models are based on assumptions as we currently lack a quantitative understanding of such complex mechanisms. Nevertheless, they point to a substantially higher $k_{off,single}$ for a single site compared to the global k_{off} recovered from SFS measurements.

Bulk transport experiment. *In vitro* reconstitution of the nucleocytoplasmic transport was achieved as previously described (Kose et al., 2015). In brief cells were washed 3x with transport buffer (20 mM Hepes, 110 mM KOAc, 5 mM NaOAc, 2 mM MgOAc, 1 mM EGTA, pH 7.3) and incubated with Hoechst33342 (1µg/mL in PBS) for 15 min. Cells were then permeabilized via incubation on ice for 10 min with digitonin (40 µg/mL), washed 3 times with transport buffer supplied with 1.5% polyvinylpyrrolidone (PVP, 360 kDa) to remove the soluble factors and digitonin before the addition of the transport mix. The transport mix consists of the purified NTRs, recycling factors and a fluorescent cargo (NLS-MBP-eGFP) with the addition of a source of energy (1 µM Importinβ, 1 µM Importinα, 4 µM Ran, 2 µM NTF2, 0.5 µM NLS-MBP-eGFP, 2 mM GTP, 1 mM DTT in transport buffer). For negative controls cells were incubated for 15 min at RT with the lectin Wheat Germ Agglutinin (WGA)(500 µg/mL) which inhibits nuclear transport before addition of the transport mix (Finlay et al., 1987). Fluorescent eGFP signal was then imaged on a commercial confocal microscope (Leica SP8, Leica Microsystems) directly after the transport mix addition for up to 1 hour.

Single molecule tracking and experimental setup. Single molecule (sm) measurements were performed on a custom made experimental setup built around a 100x UAPON Olympus TIRFM

high N.A. objective. A 488 (150 mW LuxX, Omicron Laser, Germany) and a 568 (100 mW Sapphire, Coherent, US) were combined (using LASER dichroics from AHF, Tubingen, Germany) and focused at the back plane of the objective. Collected fluorescent light was then separated with a custom dichroic mirror and split on two high sensitivity EMCCD cameras for the green and red fluorescent channel (iXon3 and iXonUltra, Andor, Ireland).
Time lapse image series of fluorescently labeled Importinβ-Alexa488 were acquired with an acquisition time of 2 ms in presence of transport mix. The transport mix is the same described above except that the cargo was, is this case, a NLS-MBP-mCherry and that instead of unlabeled Importinβ, 15 nM Importinβ-Alexa647 and sm concentration (~pM) of Importinβ-Alexa488 were added. Fluorescently labeled Importinβ-Alexa488 positions were then detected (based on a Maximum Likelihood Ratio) in each frame and localized (fitting with a symmetrical 2D Gaussian function) with the Localizer Package (Dedecker et al., 2012) for IgorPro (Wavemetrics). Tracks were then reconstructed with the nearest neighbor algorithm implemented in the Localizer Package for IgorPro: if two particles appeared within a distance of less than 400 nm and 3 frames (6 ms) the particles were connected into a trajectory. To select the trajectories corresponding to particles interacting with the NPC, the position of the latter was determined starting from an ensemble image of Importinβ labeled with Alexa594. Since NPCs appear as diffraction limited spots, their position was detected with the same methodology adopted for single particles. The nuclear envelope shape was then reconstructed by fitting a 2D spline to NPCs position. For the analysis tracks were then selected for crossing events, i.e. requiring the initial and the final detected position in the trajectory to be on opposite side of the fitted NE (respectively outside and inside of the nucleus) at a minimum distance of 70 nm from the NE itself.

MD simulations, Nup153FG^{PxFG} unbound. Nup153FG^{PxFG} fragment was modeled on the base of its sequence that also included the exogenously inserted residues used for the labeling of the fragment with fluorophores. After energy minimization of the structure, the fragment was still partially stretched. The fragment was simulated using CHARMM22*(Lindorff-Larsen et al., 2010) and AMBER99-SB*(Piana et al., 2011) forcefields after being placed in a box of TIP3P water molecules (Jorgensen et al., 1983; Pronk et al., 2013) and 100 mM of Na^+ and Cl^- ions having dimensions 7.113 x 6.79 x 4.98 nm^3. After solvation, the solvent was equilibrated for 100 ps during which the atoms of the protein were kept at the energy minimized Cartesian coordinates by a spring constant of 1000 kJ mol^{-1} nm^{-2}. Equilibration of the system was achieved through two phases, first a canonical ensemble (NVT) phase (isochoric environment) in which the velocities for each particle were generated and a V-rescale thermostat was used to couple the temperature of the system, then a second isobaric-isothermal ensemble (NPT) phase, in which pressure was coupled using the Parrinello-Rahaman barostat (Teraoka, 2002). After equilibration, molecular dynamics trajectories were recorded for 150 ns in 15 independent simulations, for which the initial sets of particle velocities were different. Based on a root mean squared deviation (RMSD) cutoff, we performed a clustering analysis of the conformational ensemble

explored by the Nup153FG^{PxFG} and, for each force field, two conformations representing central structures of the most numerously populated clusters were picked randomly to perform the MD simulations in presence of Importinβ^{N}. The clustering analysis was carried out after concatenating the trajectories collected for all the simulations and choosing a RMSD cutoff of 0.1 nm for the backbone+Cβ atoms of the protein. All simulations were performed using the GROMACS 4.5.5 package (Pronk et al., 2013).

We note that the Nup153FG^{PxFG} conformers exhibit an average R_g below the experimental values (Mercadante et al., 2015), owing to the well-known tendency of canonical force fields including AMBER99-sb*-ILDN and CHARMM22* to favor globe-like structures (Lindorff-Larsen et al., 2010; Mercadante et al., 2015; Piana et al., 2015) and to the lack of net charged amino acids along the sequence of the simulated Nup153^{PxFG}.

MD simulations, Nup153FG^{FxFG} unbound. The N-terminal fragment of Nup153FG comprising residues 883 to 993 and named as Nup153FG^{FxFG} (FxFG rich region I, see sequences) was simulated similarly to the Nup153FG^{PxFG} fragment described above. With respect to the simulations sampling the dynamics of the Nup153FG^{PxFG} the simulation box used to sample the dynamics of the Nup153FG^{FxFG} was, however, bigger (14.256 x 12.798 x 6.926 nm^3) in order to contain such a longer fragment, which is composed, in this case, of 114 residues. Water model and all the other parameters used to run MD simulations were adapted from the previous simulations ran on Nup153FG^{PxFG}.

MD simulations Nup153FG^{PxFG} in the presence of Importinβ^{N}. Simulations were set up choosing two conformations of Nup153FG^{PxFG} as described above and were performed using the same setup described for unbound Nup153FG^{PxFG}. Nup153FG^{PxFG} was randomly placed in a box of dimensions 15 x 15 x 15 nm^3 together with the N-terminal segment of Importinβ ("PDB: 1F59" (Bayliss et al., 2000)). The choice of selecting only the N-terminal half of Importinβ for the MD simulations is justified by the observation that Importinβ is modularly built and the C-terminal end is highly similar to the N-terminal end (Bayliss et al., 2000). Indeed, several FG-Nups binding pockets at the C-terminal end of Importinβ have been predicted (and confirmed by computational and experimental studies) through a simple sequence alignment and structural modeling of the C-terminal using the binding pockets identified on the N-terminal part of the protein as a template (Isgro and Schulten, 2005). The initial positions of Nup153FG^{PxFG} with respect to Importinβ were chosen randomly, so that the binding would not be biased by the initial orientation or distance to the binding partners. Five independent 100 ns-long simulations were performed on each system for a total of 20 simulations (5 simulations for each of the two most numerous clusters using both force fields, AMBER99-SB* and CHARMM22*).

Binding was detected by measuring an increase in the contact area between the two partners. Such an area has been calculated by rolling a spherical probe with a diameter of 1.4 Angstroms on the surface of the partners alone and in complex. The resulting contact area is therefore given by half of the difference between the solvent accessible areas calculated for the complex and the

sum of the solvent accessible areas calculated for each binding partner unbound ((SAS[complex] - (SAS[Importinβ] + SAS[Nup153]))/2) (**Figure 4**). Importantly, the ability to bind, as well as the time of binding, did not appear to be correlated to the initial distance at which the binding partners were placed in the simulation system.

MD simulations Nup153FG^{FxFG} in the presence of Importinβ^{N}. The binding of Nup153FG^{FxFG} to Importinβ^{N} was sampled as previously described for the simulations involving Importinβ^{N} and Nup153FG^{PxFG}. Nevertheless, in this case the size of the system was different for different simulations in order to enhance computational efficiency. Indeed the size of the solvent box in which the binding partners were incubated varied accordingly to their distance, yielding systems with a different number of atoms ranging between 166,162 and 520, 540 particles.

BD simulations Nup153FG^{PxFG} in the presence of Importinβ^{N}. To perform BD simulations, the binding partners in the Nup153FG^{PxFG}•Importinβ^{N} complex, obtained from MD, were separated and placed at an initial distance of 40 nm. The analysis of the complex obtained from MD allowed the identification of a set of contacts used to define the formation of fruitful complexes between the partners. A fruitful complex was recorded when 1 or more contacts (up to 4) were independently satisfied according to the distances shown in **Table S2E** and set after a structural analysis of the obtained complex. Rotational and translational diffusion coefficients of the two binding partners were retrieved experimentally and used for the simulation of diffusional association. 25,000 trajectories were generated in each run and simulations were performed to sample, independently, the association of Importinβ^{N} and Importinβ$^{I178D/Y255A}$ to Nup153^{PxFG}. In order to test the contribution of apolar desolvation and electrostatics to the estimated reaction rates hydrophobic and electrostatic interactions were ignored. The determination of the rate describing the association of Importinβ^{N} and Importinβ$^{I178D/Y255A}$ and Nup153FG^{PxFG} was followed by a bootstrap analysis of the results to determine the standard deviations of the computed rates.

Supplemental Tables

Table S1, Related to Figure 4: Nup153FG^{PxFG}-Importinβ^{N} binding - details from MD simulations. Summary of the different simulations of Nup153FG^{PxFG} in presence of Importinβ^{N} both with AMBER99-sb* and CHARMM22* force fields. Time of binding is defined as in **Supplemental Experimental Procedures** and binding positions are numbered as in (Isgro and Schulten, 2005). 'New' indicates binding spots not previously identified, and 'not known binding spot' indicates that this spot does not directly involve HEAT repeats

**Force field**	**Cluster ID - Simulation ID**	**Binding**	**Time of binding**	**Binding position on Importinβ**
AMBER	7811 - 1	Yes	13 ns	#1 (HEAT repeats 3/4)
AMBER	7811 - 2	Yes	2 ns	#1 (Heat repeats 3/4)
AMBER	7811 - 3	No	-	-
AMBER	7811 - 4	No	-	-
AMBER	7811 - 5	Yes	12 ns	#5 (HEAT repeats 8/9)
CHARMM	3450 - 1	Yes	67 ns	#1 (HEAT repeats 3/4)
CHARMM	3450 - 2	Yes	50 ns	#2, #3<br>(HEAT repeats 5/6, 6/7)
CHARMM	3450 - 3	Yes	37 ns	Not known binding spot
CHARMM	3450 - 4	No	-	-
CHARMM	3450 - 5	No	-	-
AMBER	2635 - 1	Yes	16 ns	#1, new<br>(HEAT repeats 3/4, 4/5)
AMBER	2635 - 2	No	-	-
AMBER	2635 - 3	Yes	10 ns	#1, new<br>(HEAT repeats 3/4, 4/5)
AMBER	2635 - 4	Yes	30 ns	#2<br>(HEAT repeats 5/6)
AMBER	2635 - 5	Yes	42 ns	Not known binding spot
CHARMM	3591 - 1	Yes	12 ns	new (HEAT repeats 10/11)
CHARMM	3591 - 2	No	-	-
CHARMM	3591 - 3	No	-	-
CHARMM	3591 - 4	No	-	-
CHARMM	3591 - 5	No	-	-

Supplemental References:

Akke, M., and Palmer, A.G. (1996). Monitoring Macromolecular Motions on Microsecond to Millisecond Time Scales by R1ρ−R1 Constant Relaxation Time NMR Spectroscopy. Journal of the American Chemical Society *118*, 911-912.

Bayliss, R., Littlewood, T., and Stewart, M. (2000). Structural basis for the interaction between FxFG nucleoporin repeats and importin-beta in nuclear trafficking. Cell *102*, 99-108.

Bednenko, J., Cingolani, G., and Gerace, L. (2003). Importin beta contains a COOH-terminal nucleoporin binding region important for nuclear transport. The Journal of cell biology *162*, 391-401.

Bernado, P., and Blackledge, M. (2009). A self-consistent description of the conformational behavior of chemically denatured proteins from NMR and small angle scattering. Biophys J *97*, 2839-2845.

Bui, K.H., von Appen, A., DiGuilio, A.L., Ori, A., Sparks, L., Mackmull, M.T., Bock, T., Hagen, W., Andres-Pons, A., Glavy, J.S., *et al.* (2013). Integrated structural analysis of the human nuclear pore complex scaffold. Cell *155*, 1233-1243.

Dedecker, P., Duwe, S., Neely, R.K., and Zhang, J. (2012). Localizer: fast, accurate, open-source, and modular software package for superresolution microscopy. Journal of biomedical optics *17*, 126008.

Delaglio, F., Grzesiek, S., Vuister, G.W., Zhu, G., Pfeifer, J., and Bax, A. (1995). NMRPipe: a multidimensional spectral processing system based on UNIX pipes. Journal of biomolecular NMR *6*, 277-293.

Ferreon, A.C., Gambin, Y., Lemke, E.A., and Deniz, A.A. (2009). Interplay of alpha-synuclein binding and conformational switching probed by single-molecule fluorescence. Proc Natl Acad Sci U S A *106*, 5645-5650.

Finlay, D.R., Newmeyer, D.D., Price, T.M., and Forbes, D.J. (1987). Inhibition of in vitro nuclear transport by a lectin that binds to nuclear pores. The Journal of cell biology *104*, 189-200.

Frey, S., and Gorlich, D. (2007). A saturated FG-repeat hydrogel can reproduce the permeability properties of nuclear pore complexes. Cell *130*, 512-523.

Hansen, D.F., Vallurupalli, P., and Kay, L.E. (2008). An improved 15N relaxation dispersion experiment for the measurement of millisecond time-scale dynamics in proteins. The journal of physical chemistry B *112*, 5898-5904.

Hillger, F., Hanni, D., Nettels, D., Geister, S., Grandin, M., Textor, M., and Schuler, B. (2008). Probing protein-chaperone interactions with single-molecule fluorescence spectroscopy. Angewandte Chemie *47*, 6184-6188.

Isgro, T.A., and Schulten, K. (2005). Binding dynamics of isolated nucleoporin repeat regions to importin-beta. Structure *13*, 1869-1879.

Jensen, M.R., Salmon, L., Nodet, G., and Blackledge, M. (2010). Defining conformational ensembles of intrinsically disordered and partially folded proteins directly from chemical shifts. Journal of the American Chemical Society *132*, 1270-1272.

Jorgensen, W.L., Chandrasekhar, J., Madura, J.D., Impey, R.W., and Klein, M.L. (1983). Comparison of simple potential functions for simulating liquid water. The Journal of Chemical Physics *79*, 926-935.

Jung, Y.S., and Zweckstetter, M. (2004). Mars -- robust automatic backbone assignment of proteins. Journal of biomolecular NMR *30*, 11-23.

Kose, S., Funakoshi, T., and Imamoto, N. (2015). Reconstitution of nucleocytoplasmic transport using

digitonin-permeabilized cells. Methods in molecular biology *1262*, 291-303.

Kramer, R.H., and Karpen, J.W. (1998). Spanning binding sites on allosteric proteins with polymer-linked ligand dimers. Nature *395*, 710-713.

Lindorff-Larsen, K., Piana, S., Palmo, K., Maragakis, P., Klepeis, J.L., Dror, R.O., and Shaw, D.E. (2010). Improved side-chain torsion potentials for the Amber ff99SB protein force field. Proteins *78*, 1950-1958.

Mercadante, D., Milles, S., Fuertes, G., Svergun, D.I., Lemke, E.A., and Grater, F. (2015). Kirkwood-Buff Approach Rescues Overcollapse of a Disordered Protein in Canonical Protein Force Fields. The journal of physical chemistry B *119*, 7975-7984.

Milles, S., Huy Bui, K., Koehler, C., Eltsov, M., Beck, M., and Lemke, E.A. (2013). Facilitated aggregation of FG nucleoporins under molecular crowding conditions. EMBO reports *14*, 178-183.

Milles, S., and Lemke, E.A. (2011). Single molecule study of the intrinsically disordered FG-repeat nucleoporin 153. Biophys J *101*, 1710-1719.

Milles, S., and Lemke, E.A. (2014). Mapping multivalency and differential affinities within large intrinsically disordered protein complexes with segmental motion analysis. Angewandte Chemie *53*, 7364-7367.

Milles, S., Tyagi, S., Banterle, N., Koehler, C., VanDelinder, V., Plass, T., Neal, A.P., and Lemke, E.A. (2012). Click strategies for single-molecule protein fluorescence. Journal of the American Chemical Society *134*, 5187-5195.

Ori, A., Banterle, N., Iskar, M., Andres-Pons, A., Escher, C., Khanh Bui, H., Sparks, L., Solis-Mezarino, V., Rinner, O., Bork, P.*, et al.* (2013). Cell type-specific nuclear pores: a case in point for context-dependent stoichiometry of molecular machines. Molecular systems biology *9*, 648.

Otsuka, S., Iwasaka, S., Yoneda, Y., Takeyasu, K., and Yoshimura, S.H. (2008). Individual binding pockets of importin-beta for FG-nucleoporins have different binding properties and different sensitivities to RanGTP. Proc Natl Acad Sci U S A *105*, 16101-16106.

Ozenne, V., Bauer, F., Salmon, L., Huang, J.R., Jensen, M.R., Segard, S., Bernado, P., Charavay, C., and Blackledge, M. (2012). Flexible-meccano: a tool for the generation of explicit ensemble descriptions of intrinsically disordered proteins and their associated experimental observables. Bioinformatics *28*, 1463-1470.

Piana, S., Donchev, A.G., Robustelli, P., and Shaw, D.E. (2015). Water dispersion interactions strongly influence simulated structural properties of disordered protein States. The journal of physical chemistry B *119*, 5113-5123.

Piana, S., Lindorff-Larsen, K., and Shaw, D.E. (2011). How robust are protein folding simulations with respect to force field parameterization? Biophys J *100*, L47-49.

Pronk, S., Páll, S., Schulz, R., Larsson, P., Bjelkmar, P., Apostolov, R., Shirts, M.R., Smith, J.C., Kasson, P.M., van der Spoel, D.*, et al.* (2013). GROMACS 4.5: a high-throughput and highly parallel open source molecular simulation toolkit. Bioinformatics *29*, 845-854.

Rasia, R.M., Lescop, E., Palatnik, J.F., Boisbouvier, J., and Brutscher, B. (2011). Rapid measurement of residual dipolar couplings for fast fold elucidation of proteins. Journal of biomolecular NMR *51*, 369-378.

Schaffer, J., Volkmer, A., Eggeling, C., Subramaniam, V., Striker, G., and Seidel, C.A.M. (1999). Identification of single molecules in aqueous solution by time-resolved fluorescence anisotropy. J Phys Chem A *103*, 331-336.

Schoch, R.L., Kapinos, L.E., and Lim, R.Y. (2012). Nuclear transport receptor binding avidity triggers a self-healing collapse transition in FG-nucleoporin molecular brushes. Proc Natl Acad Sci U S A *109*,

16911-16916.

Shammas, S.L., Travis, A.J., and Clarke, J. (2014). Allostery within a transcription coactivator is predominantly mediated through dissociation rate constants. Proc Natl Acad Sci U S A *111*, 12055-12060.

Shen, Y., and Bax, A. (2007). Protein backbone chemical shifts predicted from searching a database for torsion angle and sequence homology. Journal of biomolecular NMR *38*, 289-302.

Solyom, Z., Schwarten, M., Geist, L., Konrat, R., Willbold, D., and Brutscher, B. (2013). BEST-TROSY experiments for time-efficient sequential resonance assignment of large disordered proteins. Journal of biomolecular NMR *55*, 311-321.

Soranno, A., Buchli, B., Nettels, D., Cheng, R.R., Muller-Spath, S., Pfeil, S.H., Hoffmann, A., Lipman, E.A., Makarov, D.E., and Schuler, B. (2012). Quantifying internal friction in unfolded and intrinsically disordered proteins with single-molecule spectroscopy. Proc Natl Acad Sci U S A *109*, 17800-17806.

Svergun, D., Barberato, C., and Koch, M.H.J. (1995). CRYSOL - A program to evaluate x-ray solution scattering of biological macromolecules from atomic coordinates. J Appl Crystallogr *28*, 768-773.

Teraoka, I. (2002). Polymer solutions : an introduction to physical properties (New York: Wiley).

Wagner, R.S., Kapinos, L.E., Marshall, N.J., Stewart, M., and Lim, R.Y. (2015). Promiscuous binding of Karyopherinbeta1 modulates FG nucleoporin barrier function and expedites NTF2 transport kinetics. Biophys J *108*, 918-927.

Zhang, H., Neal, S., and Wishart, D.S. (2003). RefDB: a database of uniformly referenced protein chemical shifts. Journal of biomolecular NMR *25*, 173-195.

Zweckstetter, M., Hummer, G., and Bax, A. (2004). Prediction of charge-induced molecular alignment of biomolecules dissolved in dilute liquid-crystalline phases. Biophys J *86*, 3444-3460.
