# Supplementary figures and images for: Plasticity of an Ultrafast Interaction between Nucleoporins and Nuclear Transport Receptors
Source: Cell. 2015 Oct 22;163(3):734–45. doi: 10.1016/j.cell.2015.09.047 (PMC4622936; doi:10.1016/j.cell.2015.09.047)

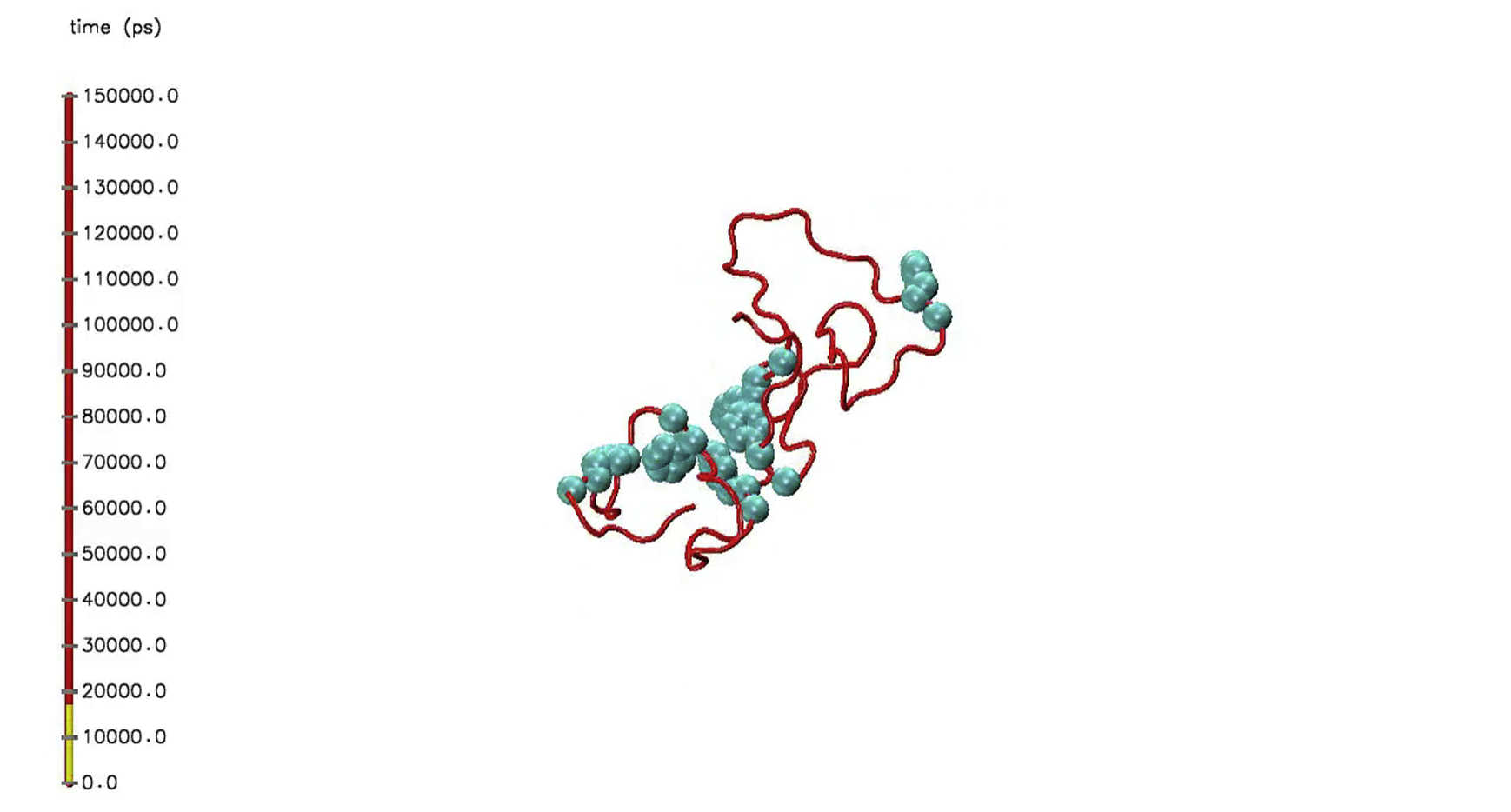

Supplement: Movie S1. Conformational Ensemble of Nup153FGPxFG, Simulated Using AMBER99-sb∗-ILDN Force Field, Related to Figure 4 — The movie shows one of the performed replicas (15 replicas each 150 ns long have been performed per force field). Protein backbone is represented as a red ribbon whereas the FG-repeats along the sequence are represented as cyan beads. On the left, simulated time is represented using a time bar and measured in ps. [file mmc3.jpg]

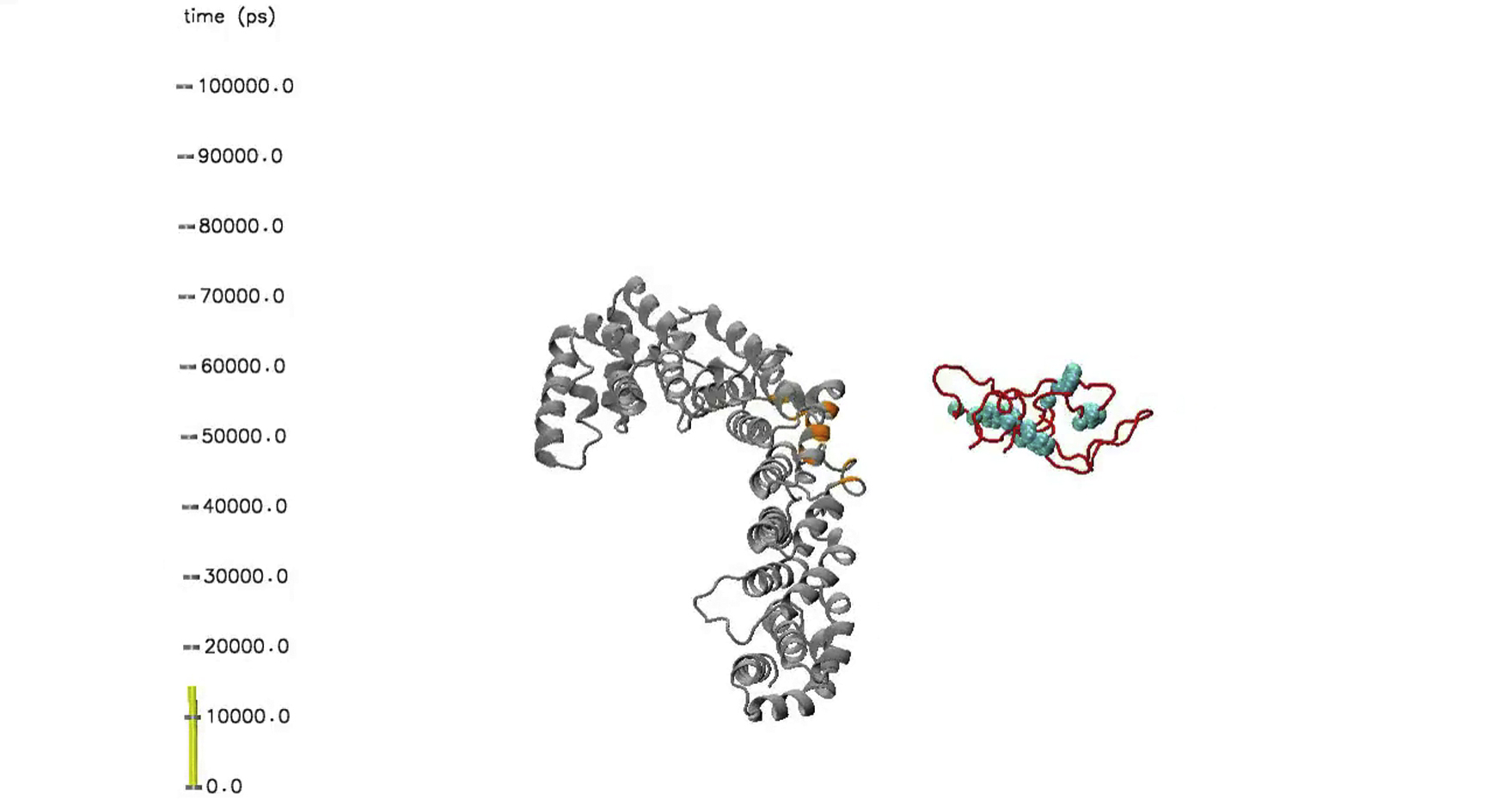

Supplement: Movie S2. Binding of Nup153FGPxFG to the Major Binding Site of ImportinβN, Related to Figure 4 — The movie shows one of the performed simulations. Nup153FGPxFG is represented as described in Movie S1. ImportinβN colored gray. The region of the protein including the major binding pocket of Nups is colored orange. On the left, simulated time is represented using a time bar and measured in ps. [file mmc4.jpg]

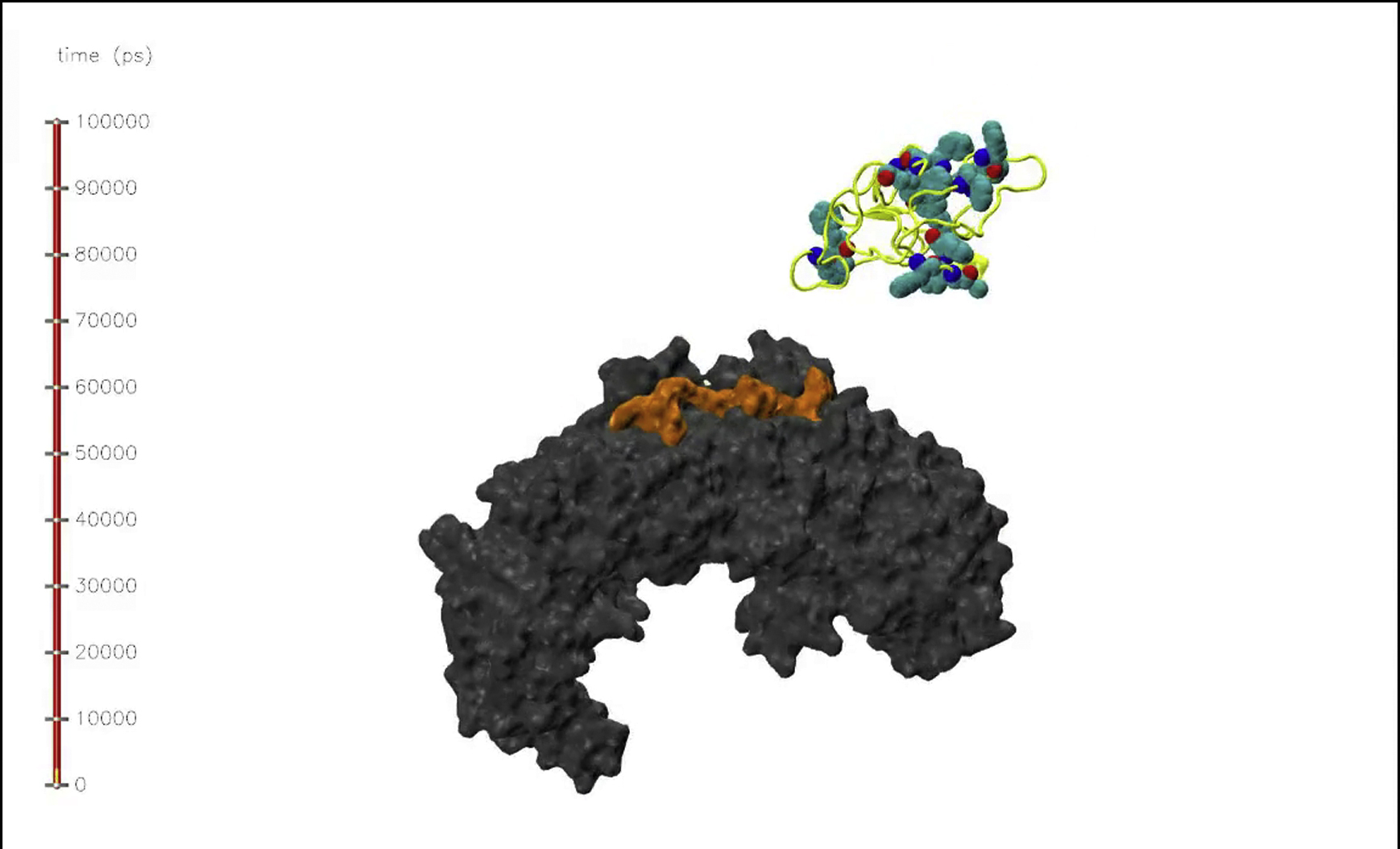

Supplement: Movie S3. Binding of Nup153FGFxFG to the Major Binding Site of ImportinβN, Related to Figure 4 — The movie shows one of the performed simulations. Nup153FGFxFG is represented as described in Movie S1. ImportinβN’s surface is shown and colored gray. The region of the protein including the major binding pocket of Nups is colored orange. On the left, simulated time is represented using a time bar and measured in ps. [file mmc5.jpg]
