# Supplementary material for: Plasticity of an Ultrafast Interaction between Nucleoporins and Nuclear Transport Receptors
Source: Cell. 2015 Oct 22;163(3):734–45. doi: 10.1016/j.cell.2015.09.047 (PMC4622936; doi:10.1016/j.cell.2015.09.047)
Supplement: Document S2. Article plus Supplemental Information [file mmc6.pdf]

Cell

Article

Plasticity of an Ultrafast Interaction between Nucleoporins and Nuclear Transport Receptors

Graphical Abstract

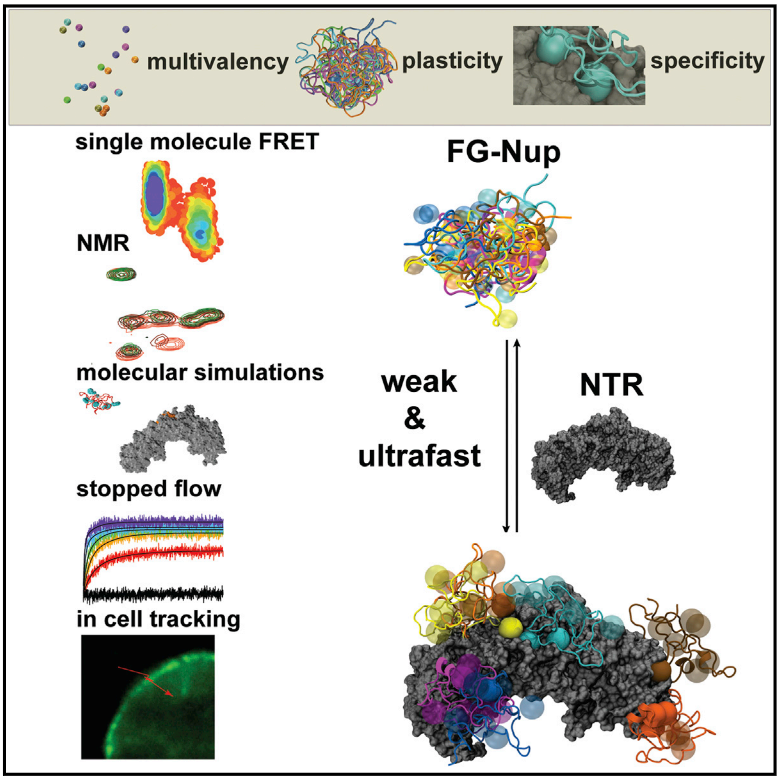

Authors

Sigrid Milles, Davide Mercadante, Iker Valle Aramburu, ..., Martin Blackledge, Frauke Gräter, Edward A. Lemke

Correspondence

martin.blackledge@ibs.fr (M.B.),
frauke.graeter@h-its.org (F.G.),
lemke@embl.de (E.A.L.)

In Brief

Intrinsically disordered nucleoporins (Nups) engage rapidly with nuclear transport receptors through many minimalistic, weakly binding motifs. These Nups form polyvalent complexes while retaining conformational plasticity thus ensuring both rapid and specific transport.

Highlights

- Integrative structural biology reveals the basis of rapid nuclear transport
- Transient binding of disordered nucleoporins leaves their plasticity unaffected
- Multiple minimalistic low-affinity binding motifs create a polyvalent complex
- A highly reactive and dynamic surface permits an ultrafast binding mechanism

Milles et al., 2015, Cell *163*, 734–745
October 22, 2015 ©2015 The Authors
http://dx.doi.org/10.1016/j.cell.2015.09.047

Cell

Article

Plasticity of an Ultrafast Interaction between Nucleoporins and Nuclear Transport Receptors

Sigrid Milles,[1,4,5,6,8] Davide Mercadante,[2,3,8] Iker Valle Aramburu,[1,8] Malene Ringkjøbing Jensen,[4,5,6] Niccolò Banterle,[1] Christine Koehler,[1] Swati Tyagi,[1] Jane Clarke,[7] Sarah L. Shammas,[7] Martin Blackledge,[4,5,6,*] Frauke Gräter,[2,3,*] and Edward A. Lemke[1,*]

[1]Structural and Computational Biology Unit, Cell Biology and Biophysics Unit, European Molecular Biology Laboratory (EMBL), Meyerhofstrasse 1, 69117 Heidelberg, Germany

[2]Molecular Biomechanics group, HITS gGmbH, Schloß-Wolfsbrunnenweg 35, 69118 Heidelberg, Germany

[3]IWR – Interdisciplinary Center for Scientific Computing, Im Neuenheimer Feld 368, 69120, Heidelberg, Germany

[4]University Grenoble Alpes, IBS, F-38044 Grenoble, France

[5]CNRS, IBS, F-38044 Grenoble, France

[6]CEA, IBS, F-38044 Grenoble, France

[7]Department of Chemistry, University of Cambridge, Cambridge CB2 1EW, UK

[8]Co-first author

*Correspondence: martin.blackledge@ibs.fr (M.B.), frauke.graeter@h-its.org (F.G.), lemke@embl.de (E.A.L.)

http://dx.doi.org/10.1016/j.cell.2015.09.047

This is an open access article under the CC BY license (http://creativecommons.org/licenses/by/4.0/).

SUMMARY

The mechanisms by which intrinsically disordered proteins engage in rapid and highly selective binding is a subject of considerable interest and represents a central paradigm to nuclear pore complex (NPC) function, where nuclear transport receptors (NTRs) move through the NPC by binding disordered phenylalanine-glycine-rich nucleoporins (FG-Nups). Combining single-molecule fluorescence, molecular simulations, and nuclear magnetic resonance, we show that a rapidly fluctuating FG-Nup populates an ensemble of conformations that are prone to bind NTRs with near diffusion-limited on rates, as shown by stopped-flow kinetic measurements. This is achieved using multiple, minimalistic, low-affinity binding motifs that are in rapid exchange when engaging with the NTR, allowing the FG-Nup to maintain an unexpectedly high plasticity in its bound state. We propose that these exceptional physical characteristics enable a rapid and specific transport mechanism in the physiological context, a notion supported by single molecule in-cell assays on intact NPCs.

INTRODUCTION

The plasticity of intrinsically disordered proteins (IDPs) is thought to be key to their highly diverse roles in the eukaryotic interactome and a variety of vital processes such as transcription, epigenetic regulation mechanisms, and transport through nuclear pore complexes (NPCs) (Dyson and Wright, 2005; Tompa and Fuxreiter, 2008). The central channel of the NPC is filled with phenylalanine-glycine-rich proteins, called FG-nucleoporins (FG-Nups) that are intrinsically disordered (Denning et al., 2003). FG-Nups build up an approximately 30-nm-thick permeability barrier through which large molecules (>40 kDa) can only be shuttled when bound to a nuclear transport receptor (NTR) with passage times as fast as 5 ms (Hoelz et al., 2011; Kubitscheck et al., 2005; Tu et al., 2013; Wälde and Kehlenbach, 2010). Due to the intrinsic dynamics of the FG-Nups, even state-of-the-art electron tomographic studies are not able to visualize them within the central NPC channel, despite their millimolar concentrations (Bui et al., 2013). Consequently, the molecular structure of the permeability barrier and its general mode of action are widely debated (for a review see Adams and Wente, 2013).

The key to understanding the observed nucleocytoplasmic transport phenomena resides in a description of the binding mode between FG-Nups and NTRs, for which a molecular analysis of the FG-Nup•NTR interaction is a prerequisite. Our current understanding of the molecular basis of FG-Nup•NTR interactions is in large part derived from X-ray crystallographic structures or molecular dynamics (MD) simulations of NTRs in the presence of short FG-peptides (up to ~13 amino acids in length) (Bayliss et al., 2000; Isgro and Schulten, 2005), as well as binding measurements with different NTRs or mutated NTR binding pockets (Bednenko et al., 2003; Milles and Lemke, 2014; Otsuka et al., 2008). Even for FG-Nups alone, only overall chain dimensions or long-range interactions within the Nups have so far been analyzed in solution (Milles and Lemke, 2011; Yamada et al., 2010). Notably, even such fundamental binding characteristics as the equilibrium dissociation constant (K_d) between Nups and NTRs are still matter of discussion - estimates range from a few nM to several mM (Bednenko et al., 2003; Ben-Efraim and Gerace, 2001; Tetenbaum-Novatt et al., 2012; Tu et al., 2013). However, high K_d (low affinity, ~mM) values are not easily compatible with high specificity of the transport process, while low K_d values (~nM range) cannot easily explain high transport rates, since these might be expected to correlate with long

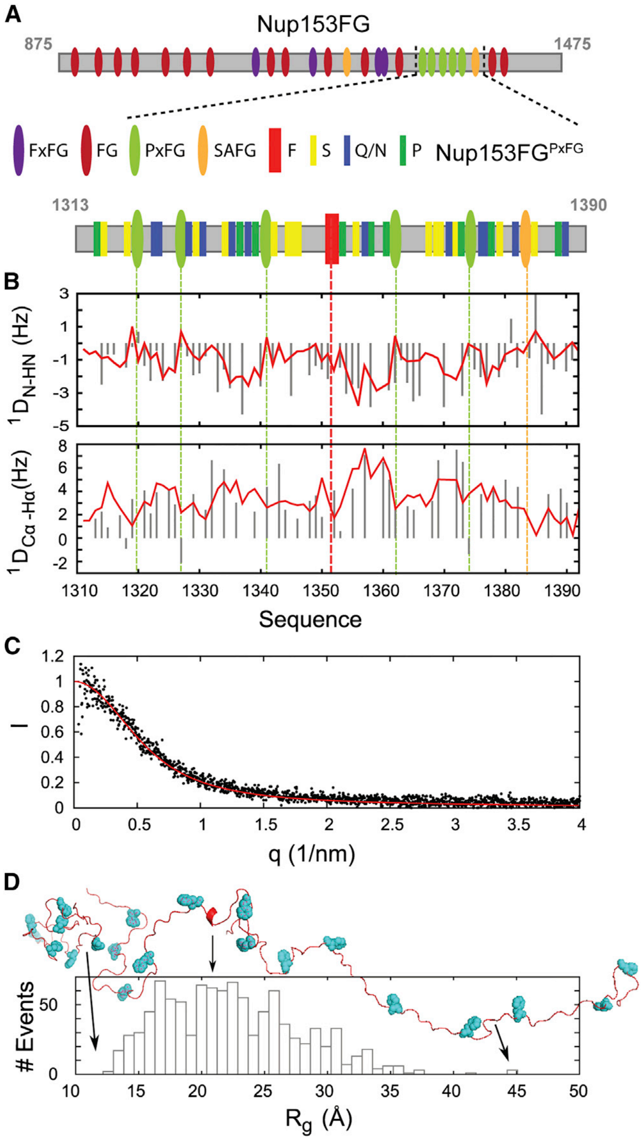

Figure 1. Conformation of Nup153FG^{PxFG}

(A) Scheme of Nup153FG constructs.

(B) Residual dipolar couplings (RDCs) of Nup153FG^{PxFG} aligned in phages. Experimentally obtained RDCs (gray bars) were compared with RDCs calculated from the ASTEROIDS ensemble obtained on the basis of experimental chemical shifts (red line). Dashed lines represent positions of FG-repeats and F1374. Color code as in (A).

(C) The same conformational ensemble was used to calculate a small angle X-ray scattering (SAXS) curve using CRYSOL (red line). The back calculated scattering curve is in good agreement with measured SAXS data under similar experimental conditions (black dots) (Mercadante et al., 2015).

(D) Distribution of the radius of gyration (R_G) from five equivalent ASTEROIDS selections. The three conformations displayed on top represent the most compact, the least compact, and one of the most prevalent conformations in the ensemble.

residence times whereas NTRs must encounter many FG-Nups while crossing the thick barrier.

Fast protein binding also typically requires proper orientation of the protein binding partners as well as conformational adaptation of the IDP to bind to a folded protein. Those can occur prior to or during binding, as described by either of the two prevalent models for protein binding namely conformational selection and induced fit (Csermely et al., 2010; Wright and Dyson, 2009). While such a conformational shift or fit can present the rate-limiting step of binding, fast binding is warranted in many biological processes. Several binding rate enhancing effects have been suggested or observed experimentally, such as maintenance of a degree of disorder (termed "fuzziness"; Tompa and Fuxreiter, 2008) by conformational funneling (Schneider et al., 2015), a large capture radius of the flexible IDPs (Shoemaker et al., 2000), and the involvement of long-range electrostatic interactions to steer (attract) proteins together (Ganguly et al., 2013).

In this work, we characterize the conformational plasticity of Nups from human and yeast in the presence of structurally and functionally diverse NTRs. A focus was a PxFG-rich domain of the Nup153 (Nup153FG^{PxFG}) as its size permitted a combination of nuclear magnetic resonance (NMR), single molecule Förster resonance energy transfer (smFRET), and molecular dynamics (MD) simulations to characterize local, residue specific, as well as long-range implications of Importinβ binding to Nup153FG^{PxFG} conformation and dynamics. Additional Brownian dynamics (BD), fluorescence stopped-flow and single molecule transport experiments with functional NPCs in permeabilized cells, revealed the detailed kinetics of the complex formation between Nup and NTR. Using this molecular, integrative structural biology approach we propose a mechanism whereby Nups contribute low-affinity minimalistic binding motifs that act in concert to create a polyvalent complex. The global Nup structure and dynamics are largely unaffected by the interaction, thereby ensuring ultrafast binding and unbinding of individual motifs—a result that explains how nuclear transport can be fast yet specific, and that may have general implications for the mechanism of action of other IDPs that exhibit a multiplicity of binding motifs.

RESULTS

Nup153FG^{PxFG} Populates a Disordered Ensemble in Solution

We initially characterized the structure and dynamics of Nup153FG^{PxFG} using high resolution NMR (Figure 1A, sequences given in Supplemental Experimental Procedure). Complete assignment of the backbone resonances (Figure S1) allowed us to develop a multi-conformational model of the protein in solution using a combination of Flexible-Meccano (Ozenne et al., 2012) and the genetic algorithm ASTEROIDS (Jensen et al., 2010). Representative ensembles comprising 200 conformers were selected on the basis of the experimental chemical shifts and were in excellent agreement with $^{1}D_{N\text{-}NH}$ and $^{1}D_{C\alpha\text{-}H\alpha}$ residual dipolar couplings and small angle X-ray scattering (SAXS) curves (Mercadante et al., 2015) that were not used in the selection process (Figures 1B–1D). The amino acid specific backbone dihedral angle distributions determined from the ensemble selections (Figure S1) show that negligible secondary structure is present.

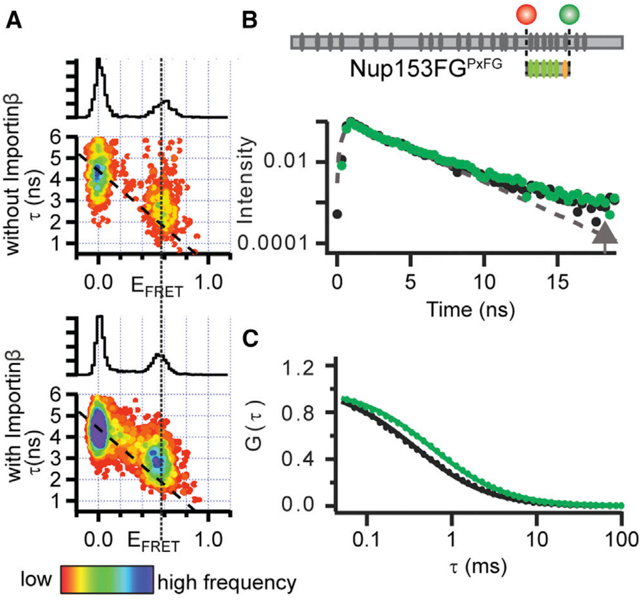

Figure 2. Nup153FG^{PxFG}•Importinβ Interaction Analyzed by smFRET

(A) FRET efficiency (E_{FRET}) versus fluorescence lifetime (τ) histograms of Nup153FG^{PxFG} in the presence and absence of Importinβ. The dotted line visualizes the center position of the FRET peak. The dashed (diagonal) lines show the static E_{FRET} relationship, on which a distribution would lie in the absence of fast dynamics.

(B) Fluorescence lifetimes (τ) of the double labeled population accumulated from single molecule data in the absence (black) and presence (green) of Importinβ. Offset from a single exponential lifetime (dashed gray curve and arrow) is a strong indicator of protein dynamics.

(C) Fluorescence correlation spectroscopy (FCS) traces retrieved from measurements of Nup153FG^{PxFG} (black dots) reflect a slower translational motion in the presence of Importinβ (green dots).

Global Structure and Dynamics of the Nup153FG^{PxFG} Are Retained upon Interaction with Importinβ as Measured by smFRET

We labeled Nup153FG^{PxFG} with a donor (Alexa488) and acceptor dye (Alexa594) for FRET at its C- and N terminus, respectively. This allowed us to measure average distance between the dyes as well as the dynamic properties of the protein using histograms relating FRET efficiency (E_{FRET}) and donor lifetimes (τ) of single molecules (sm), a method widely used to detect even minute changes in structure and dynamics, for example when IDPs bind, fold or expand (Kalinin et al., 2010; Milles and Lemke, 2011; Schuler and Eaton, 2008).

We added unlabeled Importinβ to the FRET labeled Nup153FG^{PxFG} and followed the smFRET response. While the diffusion of Nup153FG^{PxFG} in the absence and presence of Importinβ confirmed the binding of Importinβ under single molecule conditions (Figures 2 and S2), we detected neither substantial changes in E_{FRET} nor in the width of the histograms indicating absence of significant changes in the distance distribution (Figure S2 shows an all F to all A negative control). Indeed, the E_{FRET} populations of the unbound and bound Nup153FG^{PxFG} also overlay very closely with respect to τ, which indicates similarly fast dynamics of both forms (Figures 2 and S2 for detailed analysis of structure and dynamics) (Kalinin et al., 2010).

As smFRET is compatible with large proteins, we were able to repeat the same experiments for the same PxFG region within the full-length Nup153FG (601 amino acids), finding similar characteristics, and suggesting that our truncated Nup153FG^{PxFG} largely retains the conformational sampling from within the whole Nup153FG (Figure S2).

In order to determine the general nature of this binding mode, we repeated the experiments with two different FxFG-rich regions of Nup153FG, as well as the GLFG-rich yeast Nup49 and several different NTRs: i) transportin 1 (TRN1), a transport receptor involved in the import of proteins containing an M9 recognition sequence, ii) nuclear transport factor 2 (NTF2), the import receptor of RanGDP and iii) chromosomal region maintenance 1 (CRM1), a major exportin. While TRN1 and CRM1 have a similar molecular weight and superhelical structure as Importinβ, NTF2 is a much smaller, β sheet-rich dimer (Cook et al., 2007; Morrison et al., 2003). As detailed in Figure S3, despite the very distinct functionalities of the different NTRs, the smFRET and FCS measurements of the different Nups and NTRs indicate similar binding characteristics as for the Nup153FG•Importinβ complex.

Interaction with Importinβ Influences Nup153FG^{PxFG} Only Locally and Transiently

To characterize the effects of Importinβ binding on Nup153FG^{PxFG} at atomic resolution, we titrated Importinβ into a solution of ^{15}N labeled Nup153FG^{PxFG} and measured ^{1}H-^{15}N HSQC spectra at different molar ratios. Peak intensities, as well as $^{1}H^{N}$ and ^{15}N chemical shifts of Nup153FG^{PxFG}, were analyzed for each titration step (Figures 3 and S4). Resonance line broadening, associated with small changes in both $^{1}H^{N}$ and ^{15}N chemical shifts, was observed around all F's in the Nup sequence (Figure 3A). Binding was clearly highly localized, and limited to F's, with only F and the immediately adjacent amino acids being affected by the interaction. Interestingly, one single F, which is not associated with a G, is also involved in binding to Importinβ, showing the largest chemical shift changes in the ^{1}H-^{15}N HSQC spectrum during titration with Importinβ (Figure 3A and S4). ^{15}N relaxation rates measured as a function of molar ratio of Importinβ suggest that, overall, the molecule remains flexible in the complex with the transverse relaxation (R_2) increasing significantly upon Importinβ titration only around the interaction sites (Figures 3C and S4), in agreement with the above smFRET-based observations that global disorder and flexibility are not affected by Importinβ binding. Carr-Purcell-Meiboom-Gill (CPMG) relaxation dispersion experiments (Figure S4) suggested that fast exchange (< 10 μs) between the bound and unbound form of Nup153FG^{PxFG} gives rise to the increased R_2 rates around the interaction sites, which makes it possible to estimate a residue-specific $K_{d,individual}$ for each position in Nup153FG^{PxFG} with Importinβ (Figures 3E, 3F and S4) from the population weighted R_2 measurements. Interestingly, the FG-specific affinities to Importinβ are not identical across the Nup153FG^{PxFG} sequence, implying a contribution of inter-FG residues to binding, although all FG-specific $K_{d,individual}$ values lie in the millimolar range.

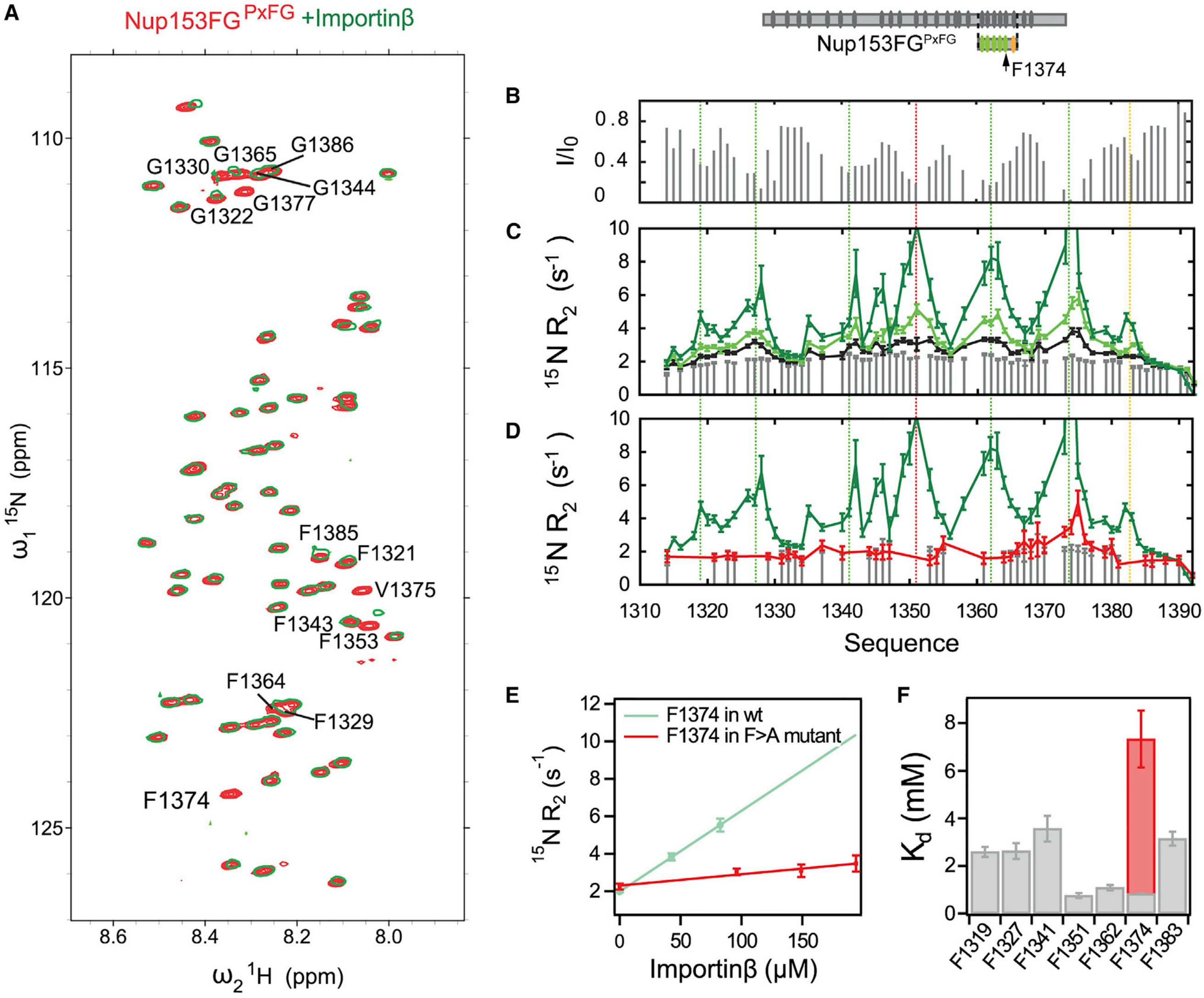

Figure 3. Nup153FG^{PxFG}·Importinβ Interaction by NMR Spectroscopy

(A) ^{1}H-^{15}N HSQC spectrum of Nup153FG^{PxFG} (red) overlayed with a spectrum of Nup153FG^{PxFG} in the presence of Importinβ (green, Nup to NTR molar ratio of 1.14, at a Nup concentration of 240 μM).

(B) The intensity ratio of the bound and unbound form of Nup153FG^{PxFG} was plotted under the same conditions as in (A).

(C) ^{15}N R_2 relaxation rates at 25°C and a ^{1}H frequency of 600 MHz were measured at different concentrations of Importinβ (gray bars are without Importinβ; black, light green and dark green at Importinβ/Nup153FG^{PxFG} molar ratios of 0.17, 0.33, and 0.72 at the constant Nup153FG^{PxFG} concentration of 250 μM).

(D) ^{15}N R_2 of Nup153AG$^{PxAG,\ F1374}$ in the absence (gray) and in the presence of Importinβ (red) overlayed with the rates for Nup153FG^{PxFG} in the presence of Importinβ under the same conditions (green).

(E) For all F in the Nup153FG^{PxFG} sequence, ^{15}N R_2 values were plotted against Importinβ concentration and fitted with a linear slope. The same analysis was performed for F1374 in Nup153AG$^{PxAG,\ F1374}$ and compared to the same F in Nup153FG^{PxFG} (compare red to green slope). R2 with errors greater than 20% were excluded from the analysis.

(F) Local K_d values were calculated from the slopes obtained in Figure S4. Gray bars correspond to K_d values obtained from Nup153FG^{PxFG}, the red bar shows the local K_d of Nup153AG$^{PxAG,\ F1374}$ binding to Importinβ.

Error bars show SD.

Strikingly, when studying the binding to different NTRs like TRN1 and NTF2 (Figure S4), despite exhibiting different binding preferences for FG-Nups (Cook et al., 2007; Milles and Lemke, 2014), their binding modes are remarkably similar to that of the Importinβ complex. The same regions in Nup153FG^{PxFG} are affected by the interaction, again with very low residue specific affinities, with the Nup remaining overall flexible when bound while interacting only locally as seen from both chemical shift changes, in the case of NTF2, and remarkably similar locally elevated transverse relaxation rates in TRN1 (Figure S4). Comparison of ^{13}C backbone chemical shifts measured in the free and NTF2-bound forms of Nup153FG^{PxFG} demonstrates that

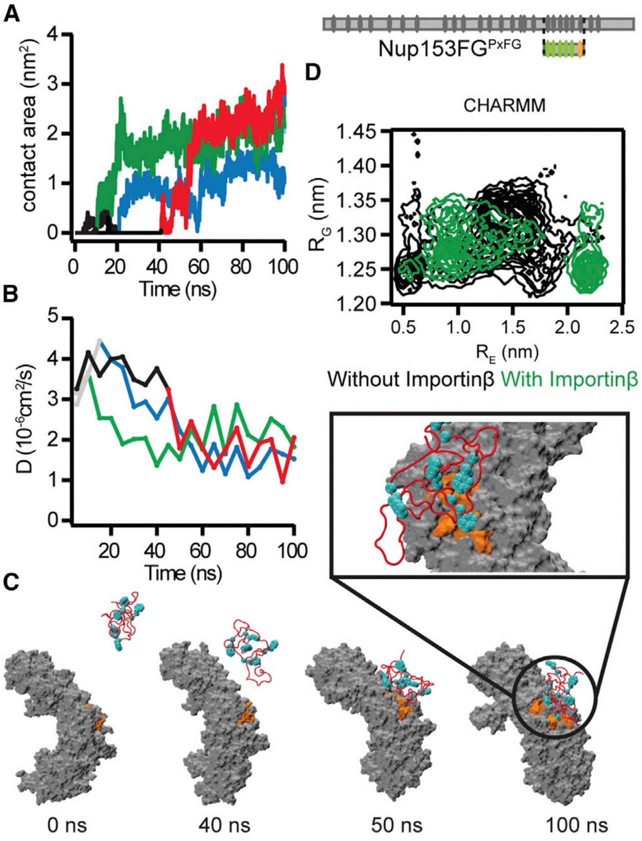

Figure 4. Binding of Nup153FG^{PxFG} to Importinβ^{N}

(A–C) Contact area between (A) Nup153FG^{PxFG} and Importinβ^{N} and (B) diffusion coefficients D as a function of time for the 4 binding events out of 10 simulations (gray/black: prior to binding; different colors: after binding; black/red curves refer to the cartoon in (C) sampled using CHARMM22* force field.

(C) Snapshots collected along one of the recorded MD trajectories showing the binding between Nup153FG^{PxFG} (red cartoon) and Importinβ^{N} (gray surface). The binding sites on Importinβ^{N} and Nup153FG^{PxFG} FG-repeats are colored in orange and cyan respectively.

(D) Nup153FG^{PxFG} radius of gyration (R_G) as a function of end-to-end distance (R_E) for the unbound (black) and bound (green) ensembles of Nup153FG^{PxFG} obtained from the simulations performed using CHARMM22*.

See Figure S5 for data using the AMBER force field.

the protein backbone remains flexible upon interaction, sampling effectively the same conformational equilibrium in the free and bound state (Figure S4).

We note that during the publication process of this work, localized interaction was also reported for the yeast Nsp1 with Kap95 (the yeast homolog of Importinβ) using NMR (Hough et al., 2015), suggesting that a similar interaction mechanism may also be conserved across species.

Co-operativity of FG-Nup·Importinβ Binding

To further quantify the action of multiple FG-repeats, we designed a Nup construct, in which all F of Nup153FG^{PxFG} except F1374, the strongest interaction site for Importinβ, were replaced by A (Figure S1). Titration of Importinβ into this Nup153AG^{PxAG,F1374} mutant resulted in strongly reduced peak broadening and negligible chemical shift changes compared to Nup153FG^{PxFG} (Figure S4). As in the case of Nup153FG^{PxFG}, ^{15}N R_2 relaxation rates of Nup153AG^{PxAG,F1374} at the interaction site exhibited a linear dependence on Importinβ concentration (Figure 3E). However the effective $K_{d,individual}$ from F1374 within Nup153AG^{PxAG,F1374} reveals significantly weaker binding for this interaction site than for F1374 when situated within the wild-type (WT) protein ($K_{d,individual}$ = 7.3 mM compared to 0.8 mM, Figure 3). This result clearly shows that presenting multiple equivalent binding sites to the binding partner has a measurably positive effect on the effective affinity of the individual interaction site.

Monitoring the Nup153FG^{PxFG}·Importinβ Binding Using All-Atom MD

We employed MD simulations to investigate the experimental observations of Nup153FG^{PxFG}·Importinβ association from NMR and smFRET. From a broad ensemble of Nup153FG^{PxFG} obtained from unbiased MD simulations in explicit solvent (Movie S1), we incubated different conformers with the N-terminal portion of Importinβ (from here named Importinβ^{N} (Bayliss et al., 2000)) and monitored their binding for a total simulation time of 2 μs (Figures S5 and S6, and Table S1). The association of Nup153FG^{PxFG} to Importinβ^{N} was repeatedly observed within the simulated timescale and occurred in a specific manner (Figures 4 and S5, and Movie S2). FG-repeats docked into previously identified binding pockets on the surface of Importinβ^{N} and even formed contacts similar to those previously observed crystallographically upon interaction between Importinβ and Nsp1-derived peptides (Figures 4C and S6) (Bayliss et al., 2000). Binding was reduced and less specific for Nup153FG^{PxAG} (Figure S5), in agreement with NMR and smFRET (Figures S1, S2, and S4).

We suggest that the high solvent exposure of Fs in the unbound state (typically contained within the hydrophobic interior of folded proteins) (Figure S5) renders them readily available for Nup153FG^{PxFG}·Importinβ^{N} association, without requiring any global structural transitions in either partner (Figures 4D, S6, Movie S2).

The ability to monitor spontaneous Nup153FG^{PxFG}·Importinβ^{N} association on the sub-microsecond timescale suggests an ultrafast association (Figure S5). Underlining the generality of our observation, we were also able to monitor such a spontaneous binding event when repeating simulations for an FxFG-rich region of Nup153 binding to Importinβ^{N} (Figure S5, Movie S3, sequences given in Supplemental Experimental Procedure). However, force field inaccuracies and limited sampling prohibit the reliable extraction of an association rate, and we therefore studied the interaction further through fluorescence stopped-flow experiments (FSF) and Brownian dynamics (BD) simulations.

FSF Experiments and BD Simulations Reveal Ultrafast Binding between Nup and Importinβ

Stopped-flow kinetics monitoring fluorescence anisotropy (*r*) can be used to study binding mechanisms and measure the association rate (k_{on}) between proteins (Shammas et al., 2013). The binding of Importinβ to Nup153FG site-specifically labeled with Cy3B elicits detectable changes in *r*, due to slowed rotational

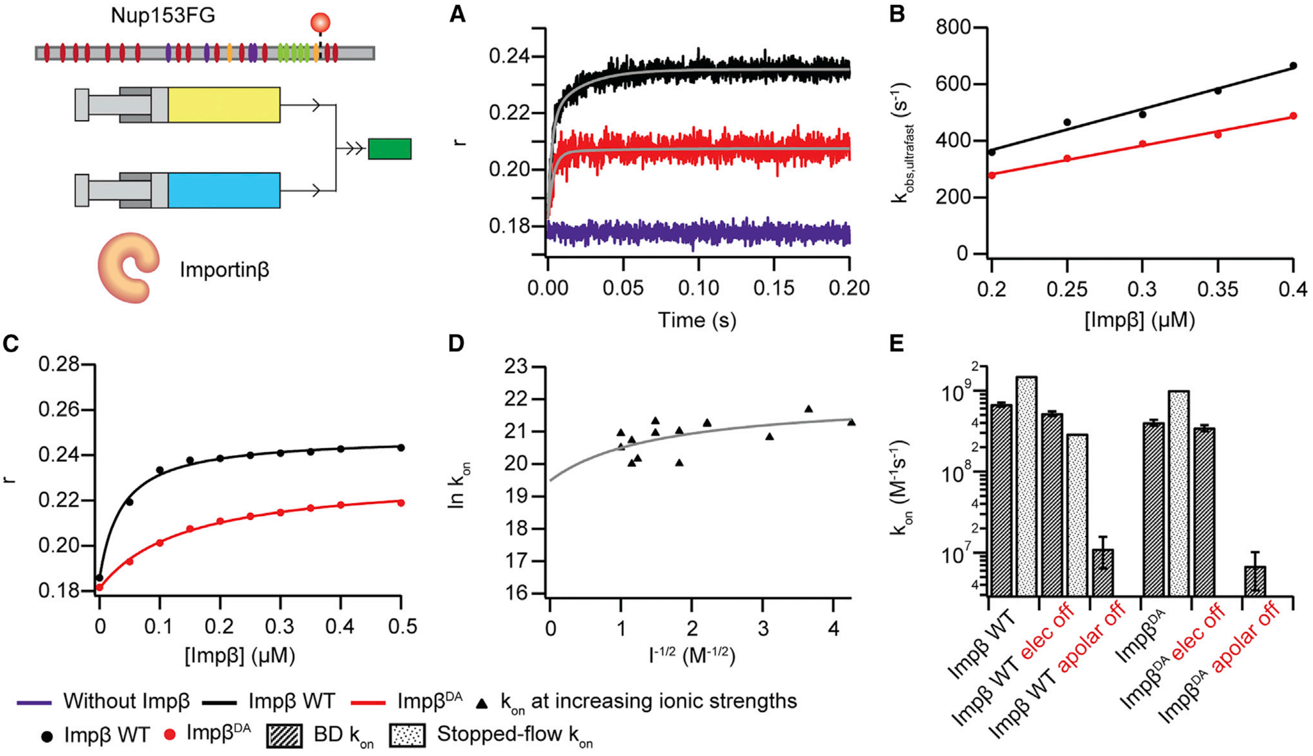

Figure 5. Association Kinetics for Nup153FG with Importinβ

(A) Stopped-flow fluorescence anisotropy was used to monitor the binding of Importinβ (Impβ) at different concentrations to Nup153FG-Cy3B. A selection of anisotropy (r) traces against time is shown for Nup153FG alone (purple) and for the binding of Importinβ WT (black) and Importinβ^{DA} (red).

(B) The observed rates ($k_{obs,ultrafast}$) from association experiments were plotted against the different Importinβ concentrations, the data were linearly fitted to obtain the association rate constants ($k_{on,ultrafast}$).

(C) Apparent $K_{d,app}$ values under the different experimental conditions.

(D) k_{on} obtained from association experiments of Nup153FG and Importinβ at different ionic strengths fitted with a Debye-Hückel-like approximation to calculate the basal rate constant at infinite ionic strength.

(E) Summary of the k_{on} values obtained from BD (dark bars) and FSF measurements (light bars) (Table S2D).

Error bars show SD.

motion (Milles and Lemke, 2014). Since Nup153FG^{PxFG} has only a very small overall binding affinity toward Importinβ, we could not detect a sufficiently strong signal change in the anisotropy measurements in the tested and experimentally feasible concentration range (Figure S7). Thus, for FSF, we used fluorescently labeled full-length Nup153FG. We performed rapid mixing experiments under pseudo-first order conditions in “physiological” transport buffer. A monoexponential function does not describe well the observed anisotropy changes in Figure 5 (Figure S7 and Table S2). This is likely a result of having multiple different binding motifs and/or the ability of multiple Importinβ to engage into binding a single Nup, which adds another level of complexity (multivalency) (Milles and Lemke, 2014; Schoch et al., 2012; Wagner et al., 2015). A biexponential equation is able to describe the kinetics, resulting in two k_{obs} per Importinβ concentration (Figures 5A, 5B, and S7). The fluorescence anisotropy at the end of the reaction was used to calculate the apparent $K_{d,app}$ (Figure 5C). Remarkably, by performing experiments at multiple NTR concentrations we extracted an ultrafast $k_{on,ultrafast} = 1.5 \cdot 10^9\ M^{-1}s^{-1}$ (Figure 5B) for the major component (average amplitude of 70%), while the second component was still very fast, with a $k_{on,fast} = 6.1 \cdot 10^7\ M^{-1}s^{-1}$ at room temperature. These FSF measurements report on overall formation of the Nup153FG• Importinβ complex i.e., one or more F binding. While we provide all results and further analysis details in Figure S7 and Table S2, for later discussion we focus on the fastest measured $k_{on,ultrafast}$.

We next estimated association rates from BD simulations, which compared to MD permit larger statistical sampling, at the cost of freezing the internal dynamics of the binding partners. Upon successful complex formation, starting from the conformations obtained from MD, the association rate was estimated (Figure S7) to be around $10^9\ M^{-1}\ s^{-1}$ (Figure 5E), in agreement with stopped-flow measurements.

BD simulations carried out without the contribution of apolar desolvation generated a drastic decrease of the estimated $k_{on,BD}$ by around two orders of magnitude, while the absence of electrostatic interactions had a negligible effect (Figures 5E and S7, and Table S2D and S2E). These observations complement our evidence for an association mainly favored by the energetic gain of sequestrating F residues from the solvent and burying them into the Importinβ^N binding pockets.

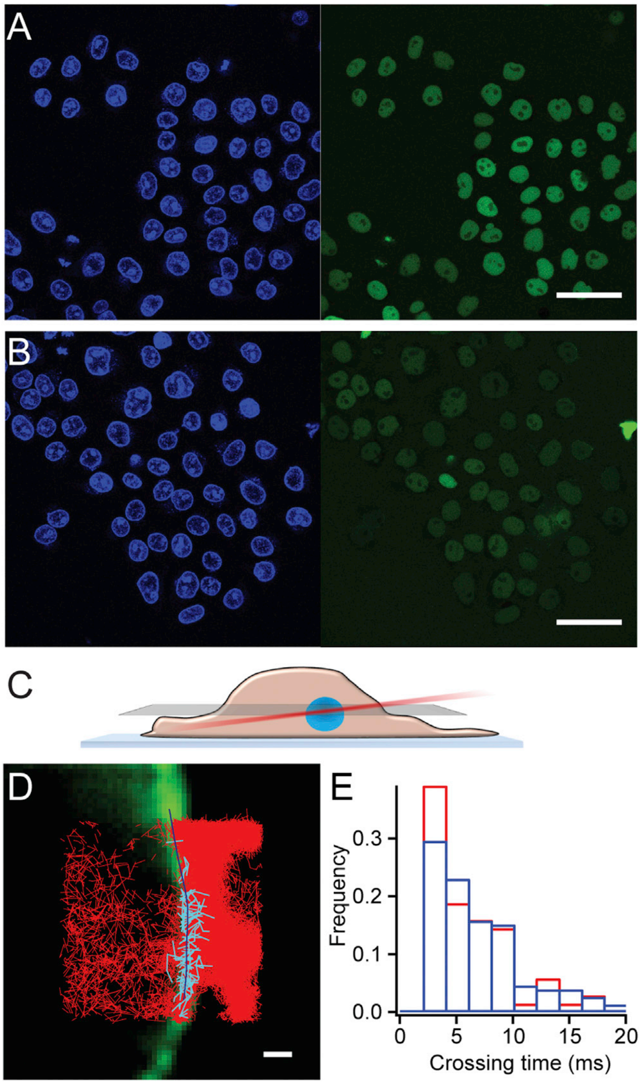

Figure 6. Nuclear Transport Assays of Importinβ and Importinβ^{DA}

(A and B) DAPI staining shown in blue, and green fluorescent cargo (NLS-MBP-eGFP) in permeabilized HeLa cells incubated with either Importinβ (A) or Importinβ^{DA} (B) (scale bar 50 μm). After 45 min, cargo accumulation is higher in the nucleus in (A).

(C) Single molecule trajectories of fluorescently labeled Importinβ were acquired in the equatorial plane of the nucleus exploiting an inclined (Hilo) illumination.

(D) Representative image of acquired single molecule trajectories of Importinβ-Alexa488 (red lines) overlaid with the ensemble image of Importinβ-Alexa647 (in green, scale bar 1μm) used to identify the nuclear envelope position (blue line). Single particle tracks of the fluorescently labeled NTR (cyan lines) crossing the nuclear envelope were analyzed to yield the characteristic barrier crossing time.

(E) The crossing time distributions reported for Importinβ (blue bars) and Importinβ^{DA} (red bars) are very fast.

While desolvation effects cannot easily be tested experimentally, high ionic strength buffers can be used to shield long-range electrostatic interactions. We thus performed a salt titration ranging from 0.05 to 1 M ionic strength (using NaCl), permitting an estimate of k_{on} under infinite electrostatic shielding by extrapolation using a Debye-Hückel-like approximation (Figures 5D and S7 and Table S2B) (Shammas et al., 2014). In line with the BD simulations, we obtained a $k_{on,elect\ off}$ of $2.9 \cdot 10^8\ M^{-1}s^{-1}$, i.e., binding remains very fast even under electrostatic shielding.

Additional stopped-flow measurements probing different Nup153FG regions (FxFG-, PxFG-rich) with diverse NTRs (NTF2, TRN1, Importinβ) are shown in Figure S7 and Table S2C. In all cases, we observed similar remarkably fast kinetics yielding consistent results for $k_{on} > 5 \cdot 10^8\ M^{-1}s^{-1}$.

Previously, solid phase binding assays indicated that the Importinβ double mutant (I178D/Y255A, termed Importinβ^{DA}) has a more than 60-fold lower K_d for binding to full-length Nup153FG as compared to Importinβ WT (Bednenko et al., 2003). $k_{on,BD}$ dropped by only 40% compared to Importinβ WT, which we confirmed by experimental FSF studies (drop of $k_{on,FSF}$ by 30%, Figure 5). However, fluorescence anisotropy measurements revealed an Importinβ^{DA} titration curve (Figure 5C) that confirms altered binding as compared to Importinβ WT, as e.g., due to an increase in k_{off}.

Single-Particle Tracking Connects Nuclear Transport of Importinβ^{DA} and Importinβ with FG-Nup Association Rates

The efficiency of an NTR to bring cargo across the NPC barrier can be assayed using standard NPC transport assays. In these assays, a fluorescent cargo (NLS-MBP-eGFP) recognized by the Importinβ transport machinery is incubated with permeabilized cells in the presence of a functional transport system and the resulting nuclear fluorescence is measured. In line with the previously reported lower K_d of Importinβ^{DA}, cargo accumulated slower compared to Importinβ WT measurements (Figures 6A and 6B) which can e.g., be due to a lower barrier crossing time, a reduced docking efficiency to the NPC or cargo release from the NPC for example.

A prediction from our kinetic analysis is that the actual speed of barrier crossing, which involves several binding and unbinding steps between NTR and FG repeats should be rather similar for WT and mutant Importinβ, as changes in k_{on} were small, and if at all, a higher k_{off} for the mutant would make crossing even faster (see discussion).

In contrast to the "bulk" transport assay, the speed of barrier crossing (characteristic crossing time) can be measured directly using single molecule (sm) tracking assays (Figure 6C), in which individual Importinβ molecules are fluorescently labeled and tracked while they cross from one side of the NPC to the other. This yielded a typical value of 6.9 ± 0.2 ms for Importinβ and 6.1 ± 0.5 ms for Importinβ^{DA} for barrier crossing (Figures 6D and 6E). We note that this crossing time is near the sampling limit of our technology, and thus faster crossing times cannot easily be captured.

DISCUSSION

The realization that many proteins are disordered has attracted considerable attention to the study of the molecular mechanisms controlling their interactions (Csermely et al., 2010; Tompa and Fuxreiter, 2008; Wright and Dyson, 2009), including the role of disorder in promoting or facilitating binding. In particular, very

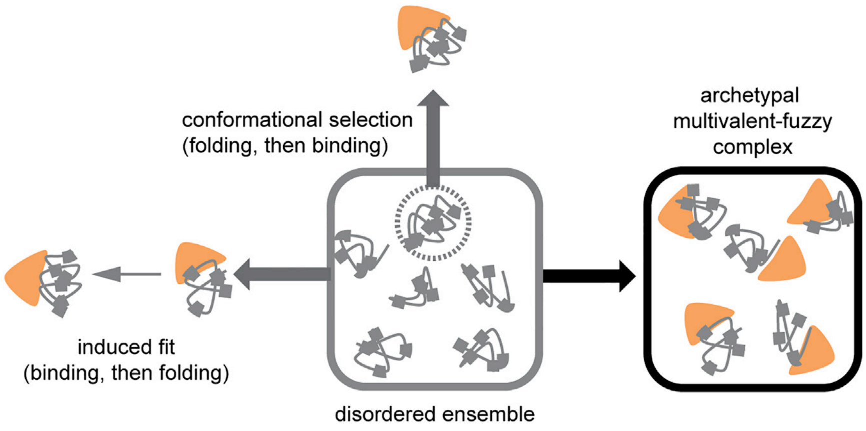

Figure 7. Binding Modes of IDPs to Folded Proteins

Schematic representation of various models describing the binding of an IDP to its folded partner. In an induced-fit mechanism the IDP partially or completely folds upon interacting with its partner, potentially showing an intermediate encounter complex as in the fly-casting mechanism (Shoemaker et al., 2000). In a conformational selection mechanism, the folded protein selects one (or several) conformation(s) of the IDP that best fits its binding pocket. These models suggest a shift in the IDP's conformational ensemble. For the Nup•NTR complex we observed formation of an "archetypal" fuzzy and multivalent complex, a binding mode that on a global scale does not require major energy or time investment for the Nup to transit from its free to the bound conformation. Note that multiple NTRs can bind one Nup and vice versa.

little is known about the binding mechanisms involved in complex processes such as nucleocytoplasmic transport, where NTRs have to engage in multiple, specific binding and unbinding events while traversing a tens of nanometer thick permeability barrier.

In this study, we have used a multidisciplinary approach to investigate the molecular mechanism underlying the interaction process between NTRs and Nups. In general, from our three core findings a coherent view emerges on how multiple rapid, yet specific protein interactions can be achieved.

Nup153FG Forms a Highly Dynamic Complex with Importinβ

Based on our smFRET measurements, we found that Nup153FG^{PxFG} resembles full-length Nup153FG with respect to its dynamics (Figures 2 and S2). Upon interaction with Importinβ, Nup153FG^{PxFG} remains flexible, engaging with Importinβ only locally, as is evident from peak broadening in the respective ^{1}H-^{15}N HSQC spectra as well as R_2 relaxation rates (Figures 3, S1, and S4). Local backbone sampling even of the interacting F was not measurably modified upon interaction. The conformers of Nup153FG^{PxFG} that were subjected to Importinβ^{N} binding in the MD simulations were also devoid of large-scale conformational changes, and interactions were only observed between individual surface exposed residues of Nup153FG^{PxFG} and Importinβ^{N}.

It appears therefore that globally, the FG-Nup maintains its conformational ensemble as shown by smFRET. This observation is sound, as IDPs frequently use motif binding to engage with their binding partners (Kragelj et al., 2015; Schneider et al., 2015; Tompa and Fuxreiter, 2008; Wright and Dyson, 2009). Our observation suggests an extraordinarily small motif (the side chain of F), which would be difficult to identify from large-scale bioinformatics approaches (Dinkel et al., 2014).

The observed binding mode appears distinct from other single motif binding interactions, as well as from mechanisms that involve global conformational transitions, such as folding upon binding (Csermely et al., 2010; Wright and Dyson, 2009) (Figure 7). The intrinsic flexibility of the Nup, the repeated occurrence and short length of the binding motif seem to create a highly reactive binding surface, which renders the individual FG-motifs prone to bind at any time without compromising the Nup's inherent plasticity.

Ultra Rapid Association of the Nup153FG•Importinβ Complex

The maximal association rate in the absence of electrostatic forces for a binary interaction system (in which all collisions are productive) can be approximated by the Einstein-Smoluchowski diffusion limit, which yields a theoretical k_{on} of $\sim 10^{9}$ $M^{-1}s^{-1}$ for the interaction of proteins of the size of Nup153FG and Importinβ.

Very high association rates have been observed previously in the presence of long-range electrostatic attractions (10^{8}-10^{10} $M^{-1}s^{-1}$) for example for the barnase/barstar interaction (Spaar et al., 2006), as well as for small IDP complexes studied by NMR (Arai et al., 2012; Schneider et al., 2015). In the absence of electrostatic steering, this upper limit is typically never reached, as successful collisions require proper orientation of the binding partners. Consequently, most experimentally observed association rates at high salt concentrations fall into the regime of 10^{4}–10^{6} $M^{-1}s^{-1}$ (Shammas et al., 2013, 2014).

Our ensemble FSF kinetics (for Nup153FG) and BD simulations (for Nup153FG^{PxFG}) show a k_{on} of $\sim 10^{9}$ $M^{-1}s^{-1}$ (Figure 5) supporting the aforementioned idea of a strongly reactive binding surface. We specifically observe an influence of apolar desolvation energies in the BD simulation and electrostatics are not found to play a major role in association. This applies apparently to both, Nup153FG^{PxFG}, which is uncharged and was tested in BD, as well as Nup153FG, which has several charges in the N-terminal regions (Figures 5D and S2). Even in the limiting case of electrostatic shielding we found complex formation to still have a remarkably fast $k_{on,FSF}$ (Figures 5D, 5E and Table S2B).

While experimentally bridging the gap between our molecular-level description of the small binary Nup•NTR complex (160 kDa) in solution to the actual in vivo transport mechanisms (involving ~120 MDa NPCs) is still a challenging quest, the sm transport

experiments (Figure 6) underline that the initially unexpected kinetic findings for the Importinβ^{DA} mutant are in line with the finding in functional NPCs.

Individual FG-Repeats Bind with Low Affinity and Act in Concert for Efficient Binding

According to ensemble titration fluorescence curves, we have observed an apparent local equilibrium constant ($K_{d,app}$) between Nup153FG and Importinβ in the nanomolar regime (Figure 5C and Table S2). However, we report millimolar affinities per FG-motif from our NMR measurements within Nup153FG^{PxFG} (Figures 3 and S4), in line with a recent computational model (Tu et al., 2013). Our NMR studies further suggest that individual FG-motifs bind independently of each other, as the ^{15}N R_2 rates are similar to the values of the unbound Nup between the FG-repeats. Nevertheless, the sum of FG-motifs influences the effective binding strength of individual FGs to Importinβ, as can be seen by comparing the effective K_d for F1374 in the WT and the Nup153AG$^{PxAG,\ F1374}$ mutant (Figure 3, S1, and S4).

While these estimates of K_d values (from NMR and ensemble fluorescence) were measured on different Nup constructs, they also report on two different properties: the binding of Importinβ to a larger region of Nup153FG (fluorescence anisotropy) and to a single FG-motif (NMR), and illustrate an important characteristic of the system, namely the importance of polyvalent interactions, which is exploited also by other transport receptors (Figure S4). While an individual FG-motif might be unlikely to be bound, the chances that at least one FG-motif within the Nup molecule is bound may remain high. This stabilizing effect of multivalency/polyvalency is well known, and is even used as a design principle in enhancing the affinity of ligand interactions with multi-site targets where ligands are connected in tandem via short linkers (Brabez et al., 2011; Kramer and Karpen, 1998). Stability enhancements achieved in such experiments can approach four-to-five orders of magnitude and are primarily due to substantial decreases in the global dissociation rate, i.e., in a multivalent system the molecules only separate as a result of a dissociation event if all other motifs are unbound.

To demonstrate generality of these three core findings, we performed additional smFRET, FCS (Figures S2 and S3), NMR (Figure S4), MD (Figure S5 and Movie S3), and FSF experiments (Figure S7 and Table S2C) on a variety of different Nups from human and yeast, including the most common motif in vertebrates (FxFG) and the crucial GLFG sequence in yeast, for a diverse set of NTRs (NTF2, TRN1, CRM1, Importinβ). All results are in close agreement, highlighting the universal nature of the observed mechanism.

Currently, several models are discussed on how a permeability barrier in the NPC can be built; among those are the selective phase, the brush, the reduction of dimensionality and the karyopherin centric model, etc., as well as mixtures of those (Eisele et al., 2013; Frey and Görlich, 2007; Jovanovic-Talisman et al., 2009; Lim et al., 2007; Lowe et al., 2015; Moussavi-Baygi et al., 2011; Peters, 2009; Wagner et al., 2015; Yamada et al., 2010). These models vary mainly over how FG-Nups are arranged and potentially interlinked inside the NPC to create a tight barrier. However, common to all these models is that the concentration of FG-repeats of about 50 mM creates a very crowded environment, which is roughly in line with stoichiometric measurements of Nups and the overall size of the central channel (Bui et al., 2013; Ori et al., 2013). Independently of the transport model assumed, mobility of an NTR inside the barrier is thus largely limited by the k_{off} and k_{on} of the interaction between FG-Nups and NTRs. This is also the case if FGs interact with FGs inside the barrier as proposed in the selective phase model (Frey and Görlich, 2007), as long as these interactions are highly dynamic and do not pose a substantial energetic barrier or rate-limiting step to be melted. That we do not observe obvious FG-FG interactions in our studies is thus not necessarily inconsistent with such a model.

If we were to naively consider the characteristic time for a single Nup and Importinβ to separate based on commonly measured fast k_{on} and affinities (e.g., K_d (Nup•NTR) ~100 nM and k_{on} ~10^6 $M^{-1}s^{-1}$ → unbinding time (UT) ~100 ms), it appears impossible that Importinβ could cross a 50 mM FG-filled pore within 5 ms. This is the previously described "transport paradox," in which high specificity is somehow coupled with rapid transport (Bednenko et al., 2003; Ben-Efraim and Gerace, 2001; Tetenbaum-Novatt et al., 2012; Tu et al., 2013).

Our work (down to picosecond and atomic resolution) is largely compatible with the existing barrier models, as it addresses on a molecular mechanistic level how an NTR could rapidly pass through a dense barrier. Using a simple model of a bivalent system, we already expect an order of magnitude difference between the dissociation rate for an individual motif and that for the whole protein (Kramer and Karpen, 1998). We have also observed extremely rapid association rates (~10^9 $M^{-1}s^{-1}$) and in Supplemental Experimental Procedures (two toy models) we outline that if we consider a very rough estimate for the characteristic time for an individual motif unbinding event (UT~1 μs) for full-length Nup153 (>24 valencies), it becomes clear that the Importinβ could "creep" through the dense FG-motif plug of the pore within the short transport time. Such movement is consistent with our (Figure 6) and other NTR diffusion studies through NPCs in intact cells and various model systems (Eisele et al., 2013; Frey and Görlich, 2007; Jovanovic-Talisman et al., 2009; Moussavi-Baygi et al., 2011; Schleicher et al., 2014; Tu et al., 2013; Wagner et al., 2015).

In this case, nature has achieved a combination of high specificity with fast interaction rates. This is based on many individual low-affinity motifs paired with a binding mode that requires relatively little energy or time investment for the Nup to transit between free and bound conformations, and provides a rationale for the fast, yet specific, nuclear transport. While rapid binding can in principle be realized between proteins of single binding elements (e.g., driven by strong electrostatics), the proofreading emanating from the multiplicity and rapid repetition of many such events is what contributes to specific transport.

We note that the transport paradox goes far beyond the relevance for the transport mechanism, since transient, but targeted interactions are central to the emerging view of highly dynamic protein (and other biomolecular) interaction networks. Furthermore, FG-repeats are also present in stress and P granules (Toretsky and Wright, 2014). It seems likely that such ultrafast binding mechanisms are also important for other biological

recognition processes, where individual interaction motifs only make weak contributions, as e.g., in the recognition of glycans (Ziarek et al., 2013), or other very short linear motifs, like WG motifs in small RNA pathways (Chekulaeva et al., 2010), or binding of proteins to epigenetic marks, like many histone modifications.

In addition, ultrafast association is achieved by using the unique plasticity of multivalent disordered proteins, which is distinct from mechanisms where orientation specific binding is required for complex formation. This represents an additional biological advantage for IDPs in comparison to folded proteins, and might have further facilitated their enrichment in organisms of higher complexity.

EXPERIMENTAL PROCEDURES

Expression and Purification of Importinβ, TRN1, NTF2, CRM1 and Nup153FG

The proteins were purified essentially as described in (Milles and Lemke, 2014) following routine column chromatography and then transferred into the respective measurement buffers. Labelling of Nup153FG (amino acids 875 to 1475 of the full length Nup153; numbering with respect to the full length protein as in 'UniProt: P49790') was performed using routine procedures to introduce Alexa488 as a donor and Alexa594 as an acceptor dye for smFRET experiments (and analog for other dyes), as described in (Milles and Lemke, 2011)

NMR Studies of Nup153FG^{PxFG}

Spectral assignments of ^{13}C, ^{15}N Nup153FG^{PxFG} were obtained from a set of BEST-TROSY-type triple resonance spectra: HNCO, intra-residue HN(CA)CO, HN(CO)CA, intra-residue HNCA, HN(COCA)CB, and intra-residue HN(CA)CB (Solyom et al., 2013). For the measurements of RDCs, ^{13}C, ^{15}N Nup153FG^{PxFG} was aligned in 12 mg/ml Pf1 phages yielding a D_2O splitting of 2.16 Hz. RDCs were measured using BEST-type HNCO and HN(CO)CA experiments (Rasia et al., 2011). ^{15}N relaxation dispersion was carried out at Nup153FG^{PxFG}/Importinβ concentrations of 250 μM and 180 μM, respectively, applying CPMG frequencies between 25 and 1,000 Hz (Schneider et al., 2015). All experiments were performed in Na-phosphate buffer (pH 6), 150 mM NaCl, 2 mM DTT, 5 mM $MgCl_2$, at 25°C and at a ^1H frequency of 600 MHz if not noted otherwise.

The conformational space available to disordered Nup153FG^{PxFG} was sampled using the *Flexible-meccano* statistical coil description (Ozenne et al., 2012) and representative ensembles in agreement with experimental chemical shifts were selected using ASTEROIDS (Jensen et al., 2010) and the ensemble was subsequently cross-validated against experimental RDCs and SAXS.

SmFRET Experiments

SmFRET measurements of dual labeled freely diffusing proteins were performed on a confocal geometry detecting donor and acceptor intensities (from which the FRET efficiency E_{FRET} is calculated) as well as fluorescence lifetimes (τ) on a custom built multiparameter setup as previously described (Milles and Lemke, 2011).

Fluorescence Stopped-Flow Experiments

The association kinetics were monitored by following the fluorescence anisotropy change of Nup153FG labeled at the indicated position with Cy3B (see sequences in Supplemental Experimental Procedures) upon binding to different concentrations of NTRs, under pseudo-first order conditions. Anisotropy (r) was calculated from fluorescence intensities measured with polarizing filters in the parallel (||) and perpendicular (⊥) position.

Each trace was obtained by averaging ≥30 traces and background fluorescence was then subtracted. The anisotropy traces where fit with a biexponential function to determine k_{obs}. The different k_{obs} were plotted against the respective NTR concentrations and were linearly fit to obtain the association constant (k_{on}) from the slope.

The used BioLogic (Grenoble, France) stopped-flow equipment permits automatic titration and repeated technical replicates, which typically yield a small standard deviation. We derived an experimental error of ~20% in k_{on} measurements between different biological replicates. To be conservative, we thus do not show (the typically lower) standard deviations from technical replicates.

Transport Experiments

Routine reconstitution of the nucleocytoplasmic transport machinery in permeabilized cells was used and fluorescence cargo (NLS-MBP-eGFP) was imaged on a confocal microscope (Leica, Mannheim) at the indicated time points.

For single molecule tracking of NTRs, the same assay was used, but Importinβ-Alexa488 at single molecule concentration was tracked with an acquisition time of 2ms on a previously described home built imaging microscope (Ori et al., 2013).

All data analyses for FSF, FCS, smFRET and tracking were performed with custom written routines in IgorPro (Wavemetrics, OR).

MD and BD Simulations

The Nup153FG^{PxFG} fragment was modeled on the basis of its sequence that also included the exogenously inserted residues used for labeling of the fragment with fluorophores. For the binding simulations, Nup153FG^{PxFG} or Nup153^{FGFxFG} were randomly placed in a box of dimensions 15 × 15 × 15 nm^3 together with the N-terminal segment of Importinβ^N (PDB: 1F59). Brownian Dynamics (BD) simulations were performed starting from the MD complex that showed a specific association between the partners, and resembled the crystallographic binding pose as reported by ref. (Bayliss et al., 2000).

ACCESSION NUMBERS

The accession number for the data reported in this paper is Protein Ensemble Database (PED): 2AAE.

SUPPLEMENTAL INFORMATION

Supplemental Information includes Supplemental Experimental Procedures, seven figures, two tables, and three movies and can be found with this article online at http://dx.doi.org/10.1016/j.cell.2015.09.047.

AUTHOR CONTRIBUTIONS

S.M., D.M., I.V.A., designed and performed experiments, analyzed data and co-wrote the manuscript. M.R.J., N.B., C.K., S.T., J.C. provided additional reagents and analysis tools. S.L.S. designed experiments and analysis methods and co-wrote the manuscript. M.B., F.G. and E.A.L. conceived the project and co-wrote the manuscript.

ACKNOWLEDGMENTS

We are grateful for helpful comments and various discussions with Cedric Debes, Martin Beck as well as the whole Lemke group. We thank Guillaume Bouvignies for help with relaxation dispersion experiments, and Damien Maurin for sample preparation. S.M. acknowledges funding from the Boehringer Ingelheim Fonds (BIF) and an EMBO long-term fellowship (ALTF 468-2014) and EC (EMBOCOFUND2012, GA-2012-600394) via Marie Curie Action. I.V.A. acknowledges a BIF short-term fellowship. J.C. and S.L.S. are supported by the Wellcome Trust. J.C. is a Wellcome Trust Senior Research Fellow (WT/095195). E.A.L. is grateful to funds from the SFB1129 and the Emmy Noether program of the DFG, F.G. from the Klaus Tschira Foundation, and D.M. from the BIOMS program of Heidelberg University. We are also grateful to instrument access via the EMBL Pepcore facility.

Received: June 25, 2015
Revised: August 17, 2015
Accepted: September 23, 2015
Published: October 8, 2015

REFERENCES

Adams, R.L., and Wente, S.R. (2013). Uncovering nuclear pore complexity with innovation. Cell *152*, 1218–1221.

Arai, M., Ferreon, J.C., and Wright, P.E. (2012). Quantitative analysis of multisite protein-ligand interactions by NMR: binding of intrinsically disordered p53 transactivation subdomains with the TAZ2 domain of CBP. J. Am. Chem. Soc. *134*, 3792–3803.

Bayliss, R., Littlewood, T., and Stewart, M. (2000). Structural basis for the interaction between FxFG nucleoporin repeats and importin-beta in nuclear trafficking. Cell *102*, 99–108.

Bednenko, J., Cingolani, G., and Gerace, L. (2003). Importin beta contains a COOH-terminal nucleoporin binding region important for nuclear transport. J. Cell Biol. *162*, 391–401.

Ben-Efraim, I., and Gerace, L. (2001). Gradient of increasing affinity of importin beta for nucleoporins along the pathway of nuclear import. J. Cell Biol. *152*, 411–417.

Brabez, N., Lynch, R.M., Xu, L., Gillies, R.J., Chassaing, G., Lavielle, S., and Hruby, V.J. (2011). Design, synthesis, and biological studies of efficient multivalent melanotropin ligands: tools toward melanoma diagnosis and treatment. J. Med. Chem. *54*, 7375–7384.

Bui, K.H., von Appen, A., DiGuilio, A.L., Ori, A., Sparks, L., Mackmull, M.T., Bock, T., Hagen, W., Andrés-Pons, A., Glavy, J.S., and Beck, M. (2013). Integrated structural analysis of the human nuclear pore complex scaffold. Cell *155*, 1233–1243.

Chekulaeva, M., Parker, R., and Filipowicz, W. (2010). The GW/WG repeats of Drosophila GW182 function as effector motifs for miRNA-mediated repression. Nucleic Acids Res. *38*, 6673–6683.

Cook, A., Bono, F., Jinek, M., and Conti, E. (2007). Structural biology of nucleocytoplasmic transport. Annu. Rev. Biochem. *76*, 647–671.

Csermely, P., Palotai, R., and Nussinov, R. (2010). Induced fit, conformational selection and independent dynamic segments: an extended view of binding events. Trends Biochem. Sci. *35*, 539–546.

Denning, D.P., Patel, S.S., Uversky, V., Fink, A.L., and Rexach, M. (2003). Disorder in the nuclear pore complex: the FG repeat regions of nucleoporins are natively unfolded. Proc. Natl. Acad. Sci. USA *100*, 2450–2455.

Dinkel, H., Van Roey, K., Michael, S., Davey, N.E., Weatheritt, R.J., Born, D., Speck, T., Krüger, D., Grebnev, G., Kuban, M., et al. (2014). The eukaryotic linear motif resource ELM: 10 years and counting. Nucleic Acids Res. *42*, D259–D266.

Dyson, H.J., and Wright, P.E. (2005). Intrinsically unstructured proteins and their functions. Nat. Rev. Mol. Cell Biol. *6*, 197–208.

Eisele, N.B., Labokha, A.A., Frey, S., Görlich, D., and Richter, R.P. (2013). Cohesiveness tunes assembly and morphology of FG nucleoporin domain meshworks - Implications for nuclear pore permeability. Biophys. J. *105*, 1860–1870.

Frey, S., and Görlich, D. (2007). A saturated FG-repeat hydrogel can reproduce the permeability properties of nuclear pore complexes. Cell *130*, 512–523.

Ganguly, D., Zhang, W., and Chen, J. (2013). Electrostatically accelerated encounter and folding for facile recognition of intrinsically disordered proteins. PLoS Comput. Biol. *9*, e1003363.

Hoelz, A., Debler, E.W., and Blobel, G. (2011). The structure of the nuclear pore complex. Annu. Rev. Biochem. *80*, 613–643.

Hough, L.E., Dutta, K., Sparks, S., Temel, D.B., Kamal, A., Tetenbaum-Novatt, J., Rout, M.P., and Cowburn, D. (2015). The molecular mechanism of nuclear transport revealed by atomic scale measurements. eLife *4*, 4.

Isgro, T.A., and Schulten, K. (2005). Binding dynamics of isolated nucleoporin repeat regions to importin-beta. Structure *13*, 1869–1879.

Jensen, M.R., Salmon, L., Nodet, G., and Blackledge, M. (2010). Defining conformational ensembles of intrinsically disordered and partially folded proteins directly from chemical shifts. J. Am. Chem. Soc. *132*, 1270–1272.

Jovanovic-Talisman, T., Tetenbaum-Novatt, J., McKenney, A.S., Zilman, A., Peters, R., Rout, M.P., and Chait, B.T. (2009). Artificial nanopores that mimic the transport selectivity of the nuclear pore complex. Nature *457*, 1023–1027.

Kalinin, S., Valeri, A., Antonik, M., Felekyan, S., and Seidel, C.A. (2010). Detection of structural dynamics by FRET: a photon distribution and fluorescence lifetime analysis of systems with multiple states. J. Phys. Chem. B *114*, 7983–7995.

Kragelj, J., Palencia, A., Nanao, M.H., Maurin, D., Bouvignies, G., Blackledge, M., and Jensen, M.R. (2015). Structure and dynamics of the MKK7-JNK signaling complex. Proc. Natl. Acad. Sci. USA *112*, 3409–3414.

Kramer, R.H., and Karpen, J.W. (1998). Spanning binding sites on allosteric proteins with polymer-linked ligand dimers. Nature *395*, 710–713.

Kubitscheck, U., Grünwald, D., Hoekstra, A., Rohleder, D., Kues, T., Siebrasse, J.P., and Peters, R. (2005). Nuclear transport of single molecules: dwell times at the nuclear pore complex. J. Cell Biol. *168*, 233–243.

Lim, R.Y., Fahrenkrog, B., Köser, J., Schwarz-Herion, K., Deng, J., and Aebi, U. (2007). Nanomechanical basis of selective gating by the nuclear pore complex. Science *318*, 640–643.

Lowe, A.R., Tang, J.H., Yassif, J., Graf, M., Huang, W.Y., Groves, J.T., Weis, K., and Liphardt, J.T. (2015). Importin-β modulates the permeability of the nuclear pore complex in a Ran-dependent manner. eLife *4*, 4.

Mercadante, D., Milles, S., Fuertes, G., Svergun, D.I., Lemke, E.A., and Gräter, F. (2015). Kirkwood-Buff Approach Rescues Overcollapse of a Disordered Protein in Canonical Protein Force Fields. J. Phys. Chem. B *119*, 7975–7984.

Milles, S., and Lemke, E.A. (2011). Single molecule study of the intrinsically disordered FG-repeat nucleoporin 153. Biophys. J. *101*, 1710–1719.

Milles, S., and Lemke, E.A. (2014). Mapping multivalency and differential affinities within large intrinsically disordered protein complexes with segmental motion analysis. Angew. Chem. Int. Ed. Engl. *53*, 7364–7367.

Morrison, J., Yang, J.C., Stewart, M., and Neuhaus, D. (2003). Solution NMR study of the interaction between NTF2 and nucleoporin FxFG repeats. J. Mol. Biol. *333*, 587–603.

Moussavi-Baygi, R., Jamali, Y., Karimi, R., and Mofrad, M.R. (2011). Brownian dynamics simulation of nucleocytoplasmic transport: a coarse-grained model for the functional state of the nuclear pore complex. PLoS Comput. Biol. *7*, e1002049.

Ori, A., Banterle, N., Iskar, M., Andrés-Pons, A., Escher, C., Khanh Bui, H., Sparks, L., Solis-Mezarino, V., Rinner, O., Bork, P., et al. (2013). Cell type-specific nuclear pores: a case in point for context-dependent stoichiometry of molecular machines. Mol. Syst. Biol. *9*, 648.

Otsuka, S., Iwasaka, S., Yoneda, Y., Takeyasu, K., and Yoshimura, S.H. (2008). Individual binding pockets of importin-beta for FG-nucleoporins have different binding properties and different sensitivities to RanGTP. Proc. Natl. Acad. Sci. USA *105*, 16101–16106.

Ozenne, V., Bauer, F., Salmon, L., Huang, J.R., Jensen, M.R., Segard, S., Bernadó, P., Charavay, C., and Blackledge, M. (2012). Flexible-meccano: a tool for the generation of explicit ensemble descriptions of intrinsically disordered proteins and their associated experimental observables. Bioinformatics *28*, 1463–1470.

Peters, R. (2009). Translocation through the nuclear pore: Kaps pave the way. BioEssays *31*, 466–477.

Rasia, R.M., Lescop, E., Palatnik, J.F., Boisbouvier, J., and Brutscher, B. (2011). Rapid measurement of residual dipolar couplings for fast fold elucidation of proteins. J. Biomol. NMR *51*, 369–378.

Schleicher, K.D., Dettmer, S.L., Kapinos, L.E., Pagliara, S., Keyser, U.F., Jeney, S., and Lim, R.Y. (2014). Selective transport control on molecular velcro made from intrinsically disordered proteins. Nat. Nanotechnol. *9*, 525–530.

Schneider, R., Maurin, D., Communie, G., Kragelj, J., Hansen, D.F., Ruigrok, R.W., Jensen, M.R., and Blackledge, M. (2015). Visualizing the molecular recognition trajectory of an intrinsically disordered protein using multinuclear relaxation dispersion NMR. J. Am. Chem. Soc. *137*, 1220–1229.

Schoch, R.L., Kapinos, L.E., and Lim, R.Y. (2012). Nuclear transport receptor binding avidity triggers a self-healing collapse transition in FG-nucleoporin molecular brushes. Proc. Natl. Acad. Sci. USA *109*, 16911–16916.

Schuler, B., and Eaton, W.A. (2008). Protein folding studied by single-molecule FRET. Curr. Opin. Struct. Biol. *18*, 16–26.

Shammas, S.L., Travis, A.J., and Clarke, J. (2013). Remarkably fast coupled folding and binding of the intrinsically disordered transactivation domain of cMyb to CBP KIX. J. Phys. Chem. B *117*, 13346–13356.

Shammas, S.L., Travis, A.J., and Clarke, J. (2014). Allostery within a transcription coactivator is predominantly mediated through dissociation rate constants. Proc. Natl. Acad. Sci. USA *111*, 12055–12060.

Shoemaker, B.A., Portman, J.J., and Wolynes, P.G. (2000). Speeding molecular recognition by using the folding funnel: the fly-casting mechanism. Proc. Natl. Acad. Sci. USA *97*, 8868–8873.

Solyom, Z., Schwarten, M., Geist, L., Konrat, R., Willbold, D., and Brutscher, B. (2013). BEST-TROSY experiments for time-efficient sequential resonance assignment of large disordered proteins. J. Biomol. NMR *55*, 311–321.

Spaar, A., Dammer, C., Gabdoulline, R.R., Wade, R.C., and Helms, V. (2006). Diffusional encounter of barnase and barstar. Biophys. J. *90*, 1913–1924.

Tetenbaum-Novatt, J., Hough, L.E., Mironska, R., McKenney, A.S., and Rout, M.P. (2012). Nucleocytoplasmic transport: a role for nonspecific competition in karyopherin-nucleoporin interactions. Mol. Cell. Proteomics *11*, 31–46.

Tompa, P., and Fuxreiter, M. (2008). Fuzzy complexes: polymorphism and structural disorder in protein-protein interactions. Trends Biochem. Sci. *33*, 2–8.

Toretsky, J.A., and Wright, P.E. (2014). Assemblages: functional units formed by cellular phase separation. J. Cell Biol. *206*, 579–588.

Tu, L.C., Fu, G., Zilman, A., and Musser, S.M. (2013). Large cargo transport by nuclear pores: implications for the spatial organization of FG-nucleoporins. EMBO J. *32*, 3220–3230.

Wagner, R.S., Kapinos, L.E., Marshall, N.J., Stewart, M., and Lim, R.Y. (2015). Promiscuous binding of Karyopherinβ1 modulates FG nucleoporin barrier function and expedites NTF2 transport kinetics. Biophys. J. *108*, 918–927.

Wälde, S., and Kehlenbach, R.H. (2010). The Part and the Whole: functions of nucleoporins in nucleocytoplasmic transport. Trends Cell Biol. *20*, 461–469.

Wright, P.E., and Dyson, H.J. (2009). Linking folding and binding. Curr. Opin. Struct. Biol. *19*, 31–38.

Yamada, J., Phillips, J.L., Patel, S., Goldfien, G., Calestagne-Morelli, A., Huang, H., Reza, R., Acheson, J., Krishnan, V.V., Newsam, S., et al. (2010). A bimodal distribution of two distinct categories of intrinsically disordered structures with separate functions in FG nucleoporins. Mol. Cell. Proteomics *9*, 2205–2224.

Ziarek, J.J., Veldkamp, C.T., Zhang, F., Murray, N.J., Kartz, G.A., Liang, X., Su, J., Baker, J.E., Linhardt, R.J., and Volkman, B.F. (2013). Heparin oligosaccharides inhibit chemokine (CXC motif) ligand 12 (CXCL12) cardioprotection by binding orthogonal to the dimerization interface, promoting oligomerization, and competing with the chemokine (CXC motif) receptor 4 (CXCR4) N terminus. J. Biol. Chem. *288*, 737–746.

Supplemental Figures

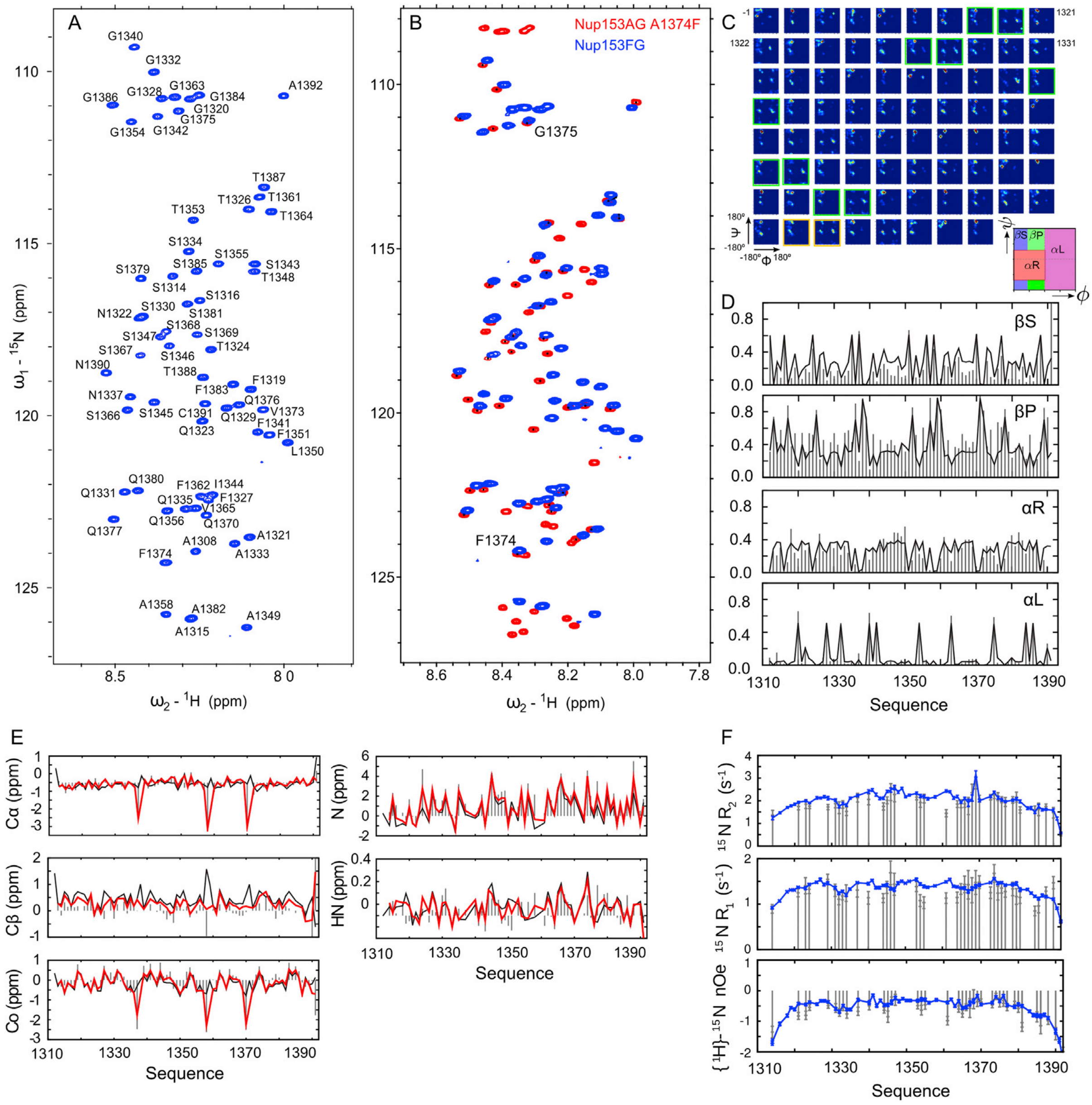

Figure S1. Conformational Behavior of Nup153FG^{PxFG}/ Nup153FG^{PxFG,F1374}, Related to Figure 1

(A) Assignment of the ^{1}H-^{15}N HSQC spectrum of Nup153FG^{PxFG}. Amino acid numbers refer to the sequence as in Uniprot P49790 (see sequences in Supplemental Experimental Procedures). Note that residues 1391 and 1392 refer to Cys and Ala for cloning and fluorescence labeling purposes rather than serines.

(B) Characterization of Nup153AG$^{PxAG,\ F1374}$. Nup153AG$^{PxAG,\ F1374}$ ^{1}H-^{15}N HSQC spectrum (red) overlaid with a spectrum of Nup153FG^{PxFG} (blue). The only F and neighboring G in the remaining FG-motif, as well as all amino acids with sufficient distances to mutated residues display very similar chemical shifts in the AG compared to the FG variant of the protein.

(C–E) Atomic resolution ensemble description of Nup153FG^{PxFG} based on experimental NMR data. (C) Ramachandran maps for all Nup153FG^{PxFG} residues as obtained from the ASTEROIDS selection. FG-repeats are color-coded as in Figure 1 (A). (D) Local conformational sampling of Nup153FG^{PxFG} showing the population of four regions of Ramachandran space (βS: β sheet; βP: Polyproline; αR and αL: right and left handed alpha helices). Gray bars result from the ASTEROIDS ensemble selected using chemical shifts. Black lines illustrate the conformational sampling of the initial statistical coil ensemble. (E) Nup153FG^{PxFG} secondary chemical shifts obtained from experiments (gray bars) and ASTEROIDS selection from a Flexible Meccano ensemble (red), based on the experimental chemical shifts. Black lines represent the initial ensemble, prior to selection.

(F) Local dynamic behavior of Nup153FG^{PxFG} and Nup153AG$^{PxAG,\ F1374}$. ^{15}N R_2, ^{15}N R_1, and {^{1}H}-^{15}N steady-state heteronuclear Overhauser effects of Nup153AG$^{PxAG,\ F1374}$ (gray bars) at a ^{1}H frequency of 600 MHz and 25°C compared to Nup153FG^{PxFG} (blue lines), showing very similar fast (ps-ns) timescale dynamics, characteristic of intrinsically disordered proteins.

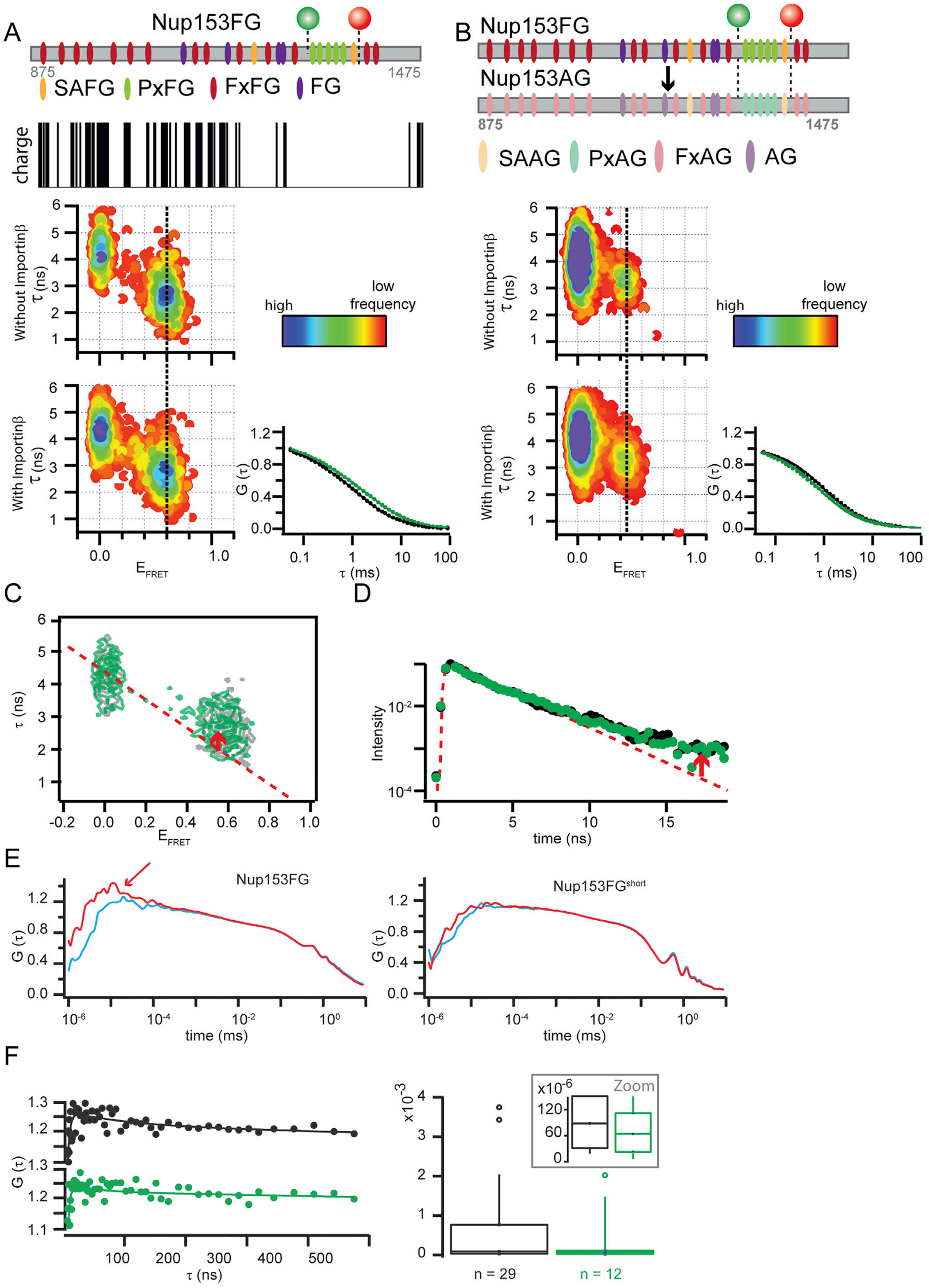

(legend on next page)

Figure S2. Structure and Dynamics of Nup153FG, PxFG-rich region, in Presence and Absence of Importinβ and Controls, Related to Figure 2

(A) Schemes of (601 aa full FG domain) Nup153FG A1391TAG S1312C FRET mutant (probing the PxFG-rich region) and $Nup153FG^{PxFG}$. Numbers indicate the selected amino acid range with respect to the full-length Nup153FG (UniProt: P49790). Donor (green, Alexa488) and acceptor (red, Alexa594) were attached on the genetically encoded unnatural amino acid acetyl-phenylalanine (AcF) and a cysteine, respectively. The 2D histograms show burst integrated τ versus E_{FRET} histograms of a double-labeled Nup153FG. The plots are color-coded for frequency of occurrence. Top and right histograms are projections along the τ and E_{FRET} axis respectively. Changes in ratio between the so called donor-only peak (arising from molecules that contain no active acceptor) and the FRET peak typically originate from photophysical effects and different labeling efficiencies between the different sample preparation of full-length Nup153FG and $Nup153FG^{PxFG}$ (see also Figure S3 for additional effects contributing to differences in observed number of events). Also shown are fluorescence correlation spectroscopy (FCS, $G(\tau)$) traces retrieved from the same measurements (black curves). Those show slower translational motion in the presence of Importinβ (green curves).

(B) Analog to (A), but data shown for an all F to A mutant termed Nup153AG, which binds Importinβ less well than the WT.

(C) Burst integrated fluorescence lifetime (τ) versus FRET efficiency (E_{FRET}) histograms of a double-labeled Nup153FG in the presence (green) and absence (gray) of Importinβ are overlaid for comparison. Dashed lines represent the static FRET lines and red arrows indicate deviations due to donor-acceptor dynamics.

(D) Fluorescence lifetimes (τ) of the double-labeled population accumulated from single molecule data in the absence (black) and presence (green) of Importinβ. The single exponential lifetime fit (dashed curve) does not adequately fit the fluorescence lifetime decay (red arrow), which is a strong indicator for protein dynamics.

(E and F) Measurements of donor-acceptor dynamics timescales are non-trivial and have in the past been extracted from a combination of nanosecond resolved FCS (nsFCS) and smFRET for a few small IDPs (Soranno et al., 2012). We measured donor-acceptor dynamics of FRET labeled Nup153FG and $Nup153FG^{PxFG}$ by splitting polarized light of the donor light channel onto three detectors. Parallel polarized light ($||$) was detected on two detectors (each recording 50% of the total intensity in the $||$ channel), perpendicular light ($\perp$) on one detector. These experiments were performed with a higher protein concentration of 5 nM. $||$ light was correlated with $||$ light (red curves) and with $\perp$ light (blue curves). The marked difference of red and blue curves in the case of Nup153FG (left plot) indicated that—due to the large size of Nup153FG compared to most previously studied proteins—fluorescence anisotropy significantly hampers analysis of the correlation curves. Small contributions from donor-acceptor fluctuations can therefore not easily be extracted. A similar observation was previously made for bigger protein complexes in accordance with our interpretation (Hillger et al., 2008). $Nup153FG^{PxFG}$ (E, right plot), however, a much smaller protein, does not pose these difficulties and correlation times can therefore be analyzed: The curves in which $||$ light was correlated with $\perp$ light were first fit with a simple diffusion model (down to $\sim$0.3 μs) in which the triplet component of the donor was extracted. The correlation curves were then normalized and plotted with a linear scale up to 500 ns (F, dots in left panel). These curves were then fit (solid lines) with the following model: $G(t) = (1-\alpha \cdot exp(t/\tau\alpha))*(1+\beta \cdot exp(t/\tau_{\beta}))(1+\gamma \cdot exp(t/\tau_{\gamma}))$, with component α the antibunching, component β the FRET dynamics and γ the triplet component, where τ_{γ} was fixed as extracted from diffusion fits. The right panel in (F) displays a statistical analysis (median 89 ns and 65 ns; 25th quantiles 31 ns and 23 ns, 75th quantile 768 ns and 112 ns without (black) and with (green) Importinβ, respectively) of the obtained fit values (note that amplitudes are so small that the fitting error is rather large). Correlations were performed with SymPhoTime (Picoquant, Berlin, Germany).

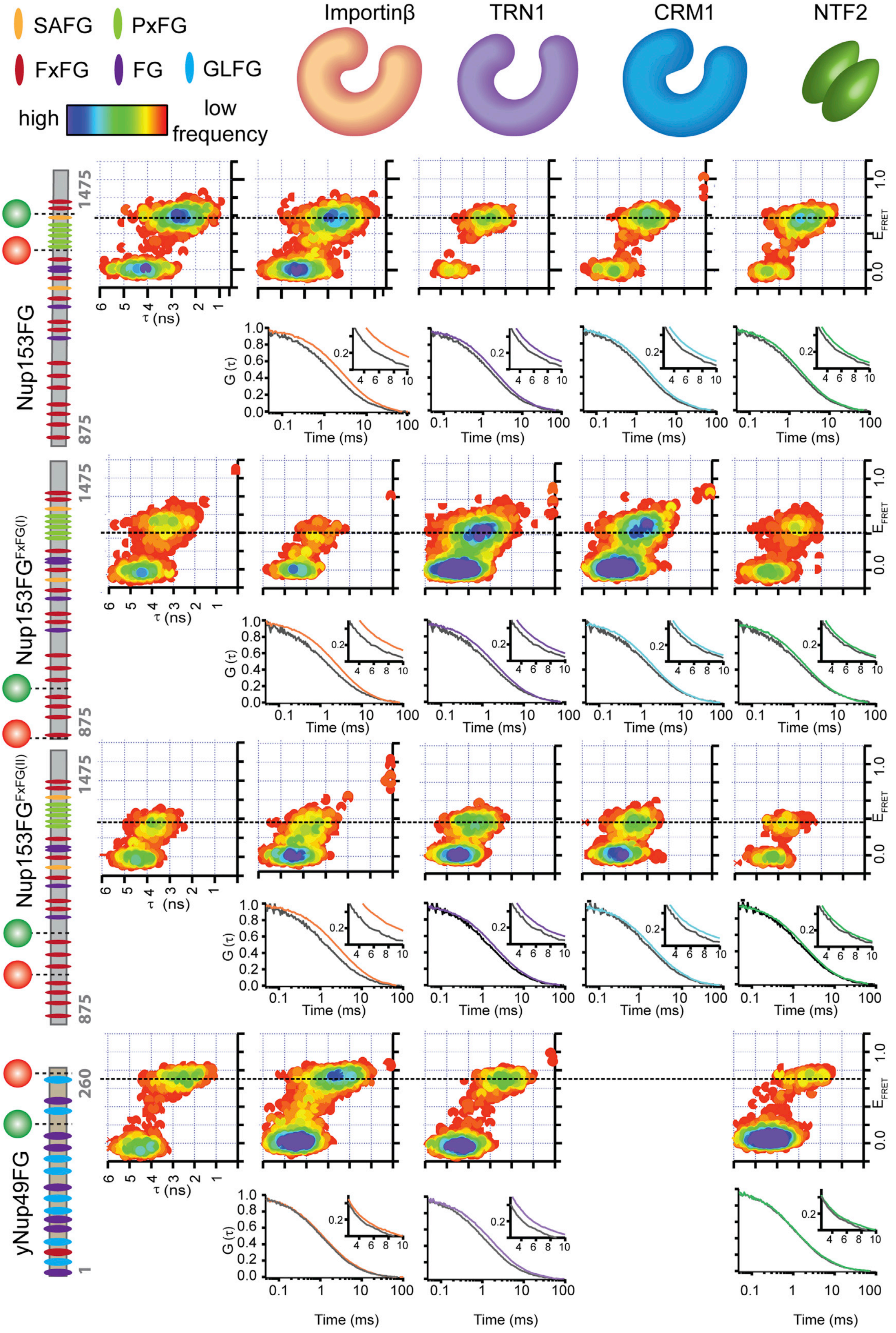

(legend on next page)

Figure S3. Conformation of Different Nucleoporin Regions in the Presence and Absence of Different NTRs Probed by smFRET, Related to Figure 2

This figure shows a cartoon of the labeling site in the respective Nup (Nup153FG or yNup49FG) on the left, as well as the resulting 2D histograms (τ versus E_{FRET}) in the presence of the respective NTR (depicted on top: Importinβ [orange], TRN1 [purple], CRM1 [blue] and NTF2 [green]). In the smFRET experiments, the Nup was used at pM concentration, and the NTR at 1 μM. Also shown are FCS experiments, were the Nups was used at 10 nM concentration. In summary, FCS detects that overall Nups are binding competent to all tested NTRs, while in the smFRET measurements no substantial conformational changes were observed in agreement with the main conclusion of this work. Note that only in the yNup49FG experiments, NTR concentration was increased to 10 μM, due to an overall lower binding affinity. However, high background in the CRM1 sample preparation did not permit high resolution single molecule FRET measurements anymore at such high concentration, and was thus excluded from this one analysis. Note also that the absolute values for FRET, depend among other factors mainly on the amino spacing of the dyes, which were different for the different constructs. Furthermore, a burst search algorithm was used to select signals from noise with a total photon count >100. In such an analysis it is inherent that the absolute values (intensities, i.e., number of events) for smFRET peaks differ depending on e.g., diffusion time (different for different sized complexes) different background (due to slight contamination from different preparations), sample sticking issues, etc.

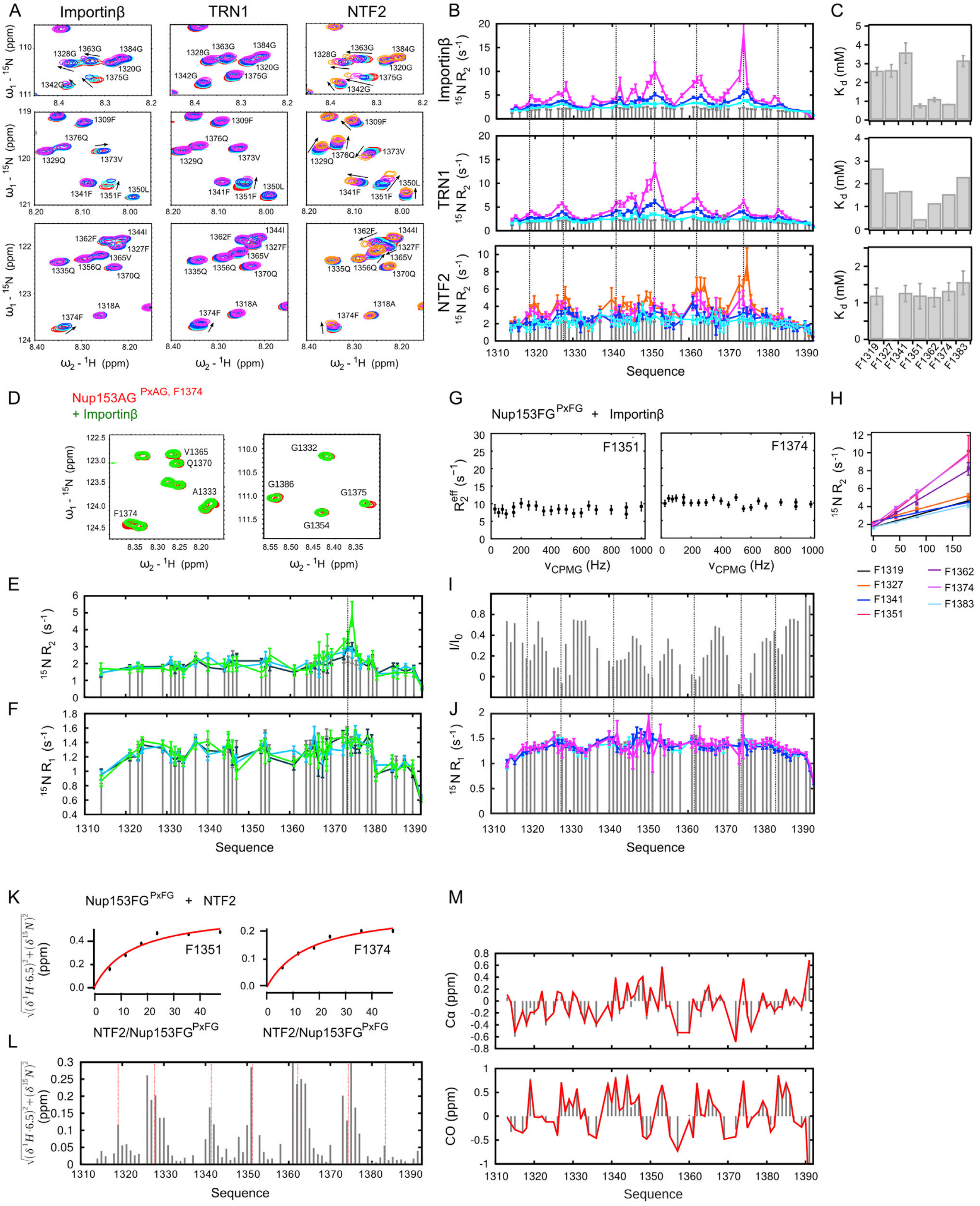

(legend on next page)

Figure S4. Interaction of Nup153FG^{PxFG} with NTRs Related to Figure 3

(A) Titration of Nup153FG^{PxFG} with different transport receptors. ^{1}H-^{15}N HSQC spectra of Nup153FG^{PxFG} in the presence of different concentrations of Importinβ (left row), TRN1 (middle row) and NTF2 (right row) were overlayed. Importinβ was added at a molar excess of 0.17 (light blue), 0.33 (dark blue) and 0.72 (magenta), TRN1 at a molar excess of 0.17 (light blue), 0.33 (dark blue), and 0.61 (magenta), and NTF2 at a molar excess of 0.33 (light blue), 1 (dark blue), 2 (magenta), and 4 (orange). Nup153FG^{PxFG} was kept at a concentration of about 250 μM throughout the titration. The spectrum of the unbound Nup153FG^{PxFG} is displayed in red. Chemical shift changes are indicated by black arrows. Spectral differences are clearly localized to the same amino acids in all cases. For NTF2 it was experimentally possible to access much higher molar ratios, and the molecular weight of the partner is much smaller than Importinβ and TRN1, explaining why larger shifts are observed.

(B) Relaxation of Nup153FG^{PxFG} at different NTR mixtures. ^{15}N R_2 of Nup153FG^{PxFG} in the presence of different concentrations of NTRs (concentrations as described in A; colored in the order of increasing NTR concentration as light blue, dark blue, magenta, and orange). ^{15}N R_2 of Nup153FG^{PxFG} alone are displayed as gray bars.

(C) Residue specific K_d values. Residue specific K_d values were extracted from the R_2 rates as described in the Supplemental Experimental Procedures. The rotational correlation time of Nup153FG^{PxFG} bound to TRN1 was assumed to be the same as when bound to Importinβ, an assumption that is justified by the similar architecture of the two transport receptors and their similar molecular weight. Since we do not have an estimate for the rotational correlation time of Nup153FG^{PxFG} bound to NTF2, we extracted the residue specific K_d values from the absolute chemical shift changes from a titration of Nup153FG^{PxFG} with NTF2 to near saturation.

(D–F) Binding of Nup153AG$^{PxAG,\ F1374}$ to Importinβ. (D) Illustration of chemical shift changes upon interaction of ^{15}N labeled Nup153AG$^{PxAG,\ F1374}$ with Importinβ. ^{1}H-^{15}N HSQC of Nup153AG$^{PxAG,\ F1374}$ (red) overlayed with a spectrum of Nup153AG$^{PxAG,\ F1374}$ in the presence of Importinβ (green, Nup at 250 μM, Importinβ at 190 μM). (E) ^{15}N R_2 relaxation rates of Nup153FG^{PxAG} were measured at different concentrations of Importinβ (gray bars are without Importinβ; dark green, blue, and light green at Importinβ/Nup153AG$^{PxAG,\ F1374}$ molar ratios of 0.38, 0.6, and 0.76 at a constant Nup153AG$^{PxAG,\ F1374}$ concentration of 250 μM). Dashed line indicates the position of F1374. (F) Associated ^{15}N R_1 relaxation rates (see E).

(G) Relaxation dispersion of Nup153FG^{PxFG} when interacting with Importinβ. CPMG relaxation dispersion measurements were performed with Nup153FG^{PxFG} bound to Importinβ at a stoichiometry ratio of 0.72 (Importinβ/nucleoporin; Nup concentration was 250 μM). The effective R_2 (R_2^{eff}) at different refocusing frequencies (υ_{cpmg}) is plotted for two representative Fs in the Nup153FG^{PxFG} sequence.

(H) Estimation of local dissociation constants. ^{15}N R_2 values plotted against Importinβ concentration (molar ratios of 0.17, 0.133, and 0.72 at the constant Nup153FG^{PxFG} concentration of 250 μM) and fitted with a slope from which FG specific K_d values were extracted under the assumption that the local rotation time (τ_c) of Nup153FG^{PxFG} bound to Importinβ equals τ_c of Importinβ itself.

(I) Intensity ratio of the bound and unbound form of Nup153FG^{PxFG} at a Nup concentration of 250 μM and Importinβ at 180 μM as obtained from two ^{1}H-^{15}N HSQC spectra.

(J) Characterization of the structure and dynamics of Nup153FG^{PxFG} in complex with Importinβ. ^{15}N R_1 of Nup153FG^{PxFG} at different concentrations of Importinβ (gray bars - without Importinβ; light blue, dark blue, magenta at Importinβ/Nup153FG^{PxFG} molar ratios of 0.17, 0.133, and 0.72 at the constant Nup153FG^{PxFG} concentration of 250 μM, as in B).

(K) Chemical shift titration of Nup153FG^{PxFG} with NTF2. Residue specific K_d values derived from the absolute chemical shift changes from a titration of Nup153FG^{PxFG} with NTF2. The data were fit with a simple binding model under the assumption of excess NTF2 (see Supplemental Experimental Procedures). Shown are titration curves for two representative Fs.

(L) Chemical shift titration of Nup153FG^{PxFG} with NTF2. Absolute chemical shift changes mapped for Nup153FG^{PxFG} in the presence of 4-fold excess of NTF2. (M) Cα and CO secondary chemical shifts obtained from Nup153FG^{PxFG} alone (gray bars) and in the presence of 24-fold excess of NTF2 at a Nup153FG^{PxFG} concentration of 80 μM (red curve), demonstrating that local conformational sampling is conserved upon interaction. Note that prolines are excluded from this plot. Error bars show SD.

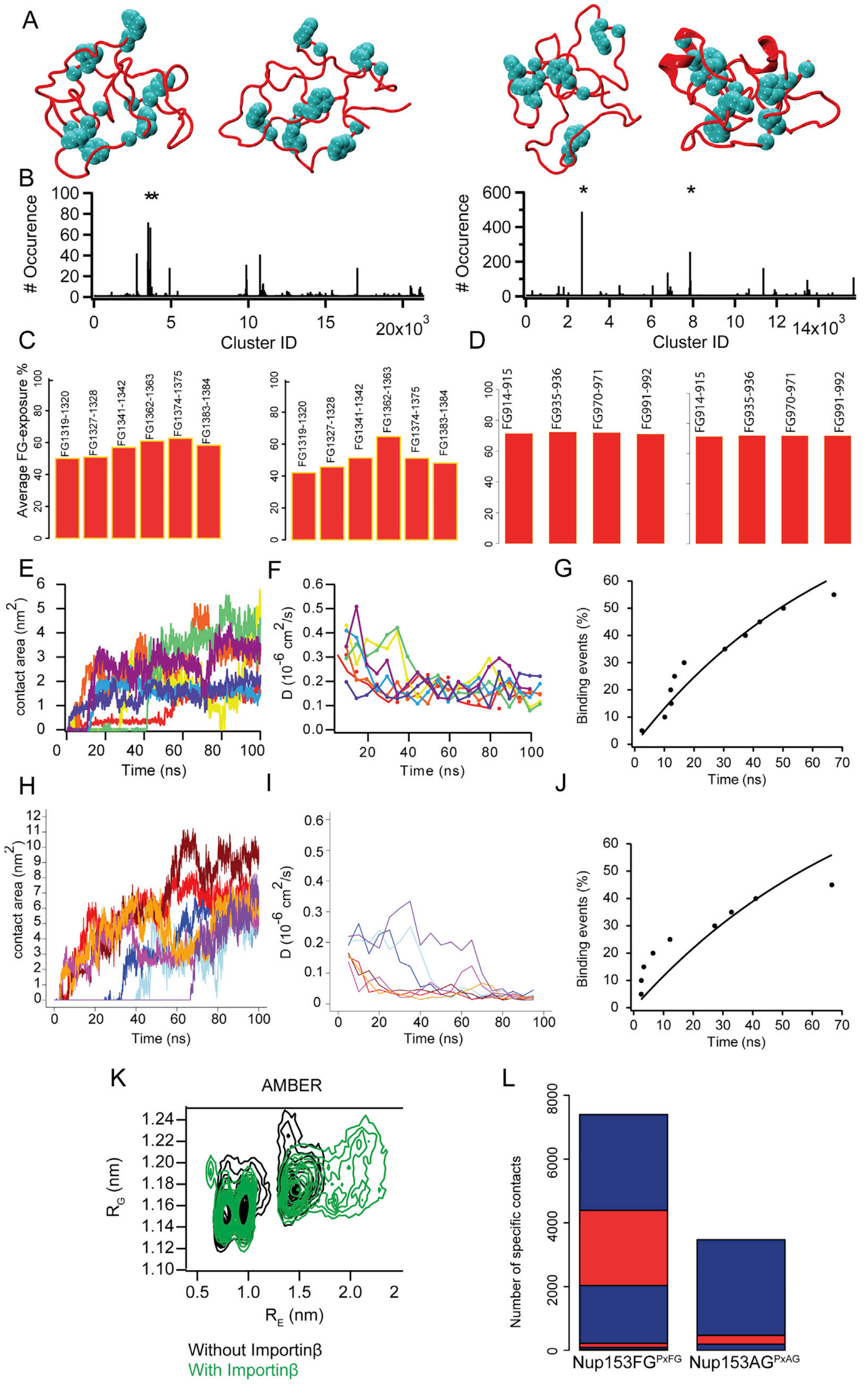

(legend on next page)

Figure S5. Computational Analysis of Nup153FG^{PxFG} and Nup153FG^{FxFG} Dynamics and of Their Binding to Importinβ^{N}, Related to Figure 4

(A) Nup153FG^{PxFG} conformations (means of the cluster) that have been selected from MD simulations in the absence of Importinβ^{N} using AMBER99-sb*-ILDN (left) and CHARMM22* (right). FG-repeats are shown by their van der Walls (vdW) radius.

(B) Count for each cluster of the simulated conformations of Nup153FG^{PxFG} as simulated with AMBER99-sb*-ILDN (left) CHARMM22* (right). Stars indicate the clusters corresponding to the conformations represented in (A).

(C and D) Average solvent exposure of Nup153FG^{PxFG} (C) and Nup153FG^{FxFG} (D) FG-repeats estimated from molecular dynamics trajectories collected from molecular dynamics simulations performed using AMBER99-sb*-ILDN (C - left panel) and CHARMM22* (D, left) force fields. The average degree of exposure has been calculated as the ratio between the mean SASA of the FG-repeat within the equilibrated Nup153FG$^{PxFG/FxFG}$ ensembles and of an FG-repeat not forming any tertiary contacts.

(E and H) Contact areas between Nup153FG^{PxFG} (E) or Nup153FG^{FxFG} (H) and Importinβ^{N} as a function of time for the binding events observed in the MD simulations performed using AMBER99-sb*-ILDN force field.

(F and I) Diffusion coefficients D, of Nup153FG^{PxFG} (F) and Nup153FG^{FxFG} (I) as a function of time are reported for the same replicas shown in (E) and (H). The drop in the diffusion coefficient is coincident with the increase in contact area upon the binding of the Nup153FG^{PxFG} and Nup153FG^{FxFG} to Importinβ^{N}.

(G and J) Percentage of binding as a function of the binding time observed in the MD runs for the simulations reporting the binding of Nup153FG^{PxFG} (G) or Nup153FG^{FxFG} (J) to Importinβ^{N}. The fit of (solid line for Nup153FG^{PxFG} - Importinβ^{N} and for Nup153FG^{FxFG} - Importinβ^{N} association) such a cumulative distribution of binding events gives a rough estimate of the $k_{on,\,MD}$ of approximately $10^{10}\ M^{-1}s^{-1}$. We note that due to the low sampling and known lower friction of water and other force field issues, (Mercadante et al., 2015) the results form MD rather define an upper limit.

(K) Nup153FG^{PxFG} radius of gyration (R_G) as a function of end-to-end distance (R_E) for the unbound (black) and bound (green) ensembles of Nup153FG^{PxFG} obtained from the simulations performed using the AMBER99-sb*-ILDN force field.

(L) Count of specific contacts has been performed for the simulations of Nup153FG^{PxFG} and Nup153AG^{PxAG} (in which all F residues were mutated to A) binding to Importinβ^{N}. Blue and red bars indicate the five different simulations performed in each case using the AMBER99-sb*-ILDN force field. In the case of the Nup153AG^{PxAG}, two out of five simulations did not yield any specific contact between the partners. Specific contacts have been considered as any contact occurring within a cutoff distance of 0.6 nm between the FG/AG-repeats of Nup153FG/AG$^{PxFG/PxAG}$ and any binding site on Importinβ^{N}.

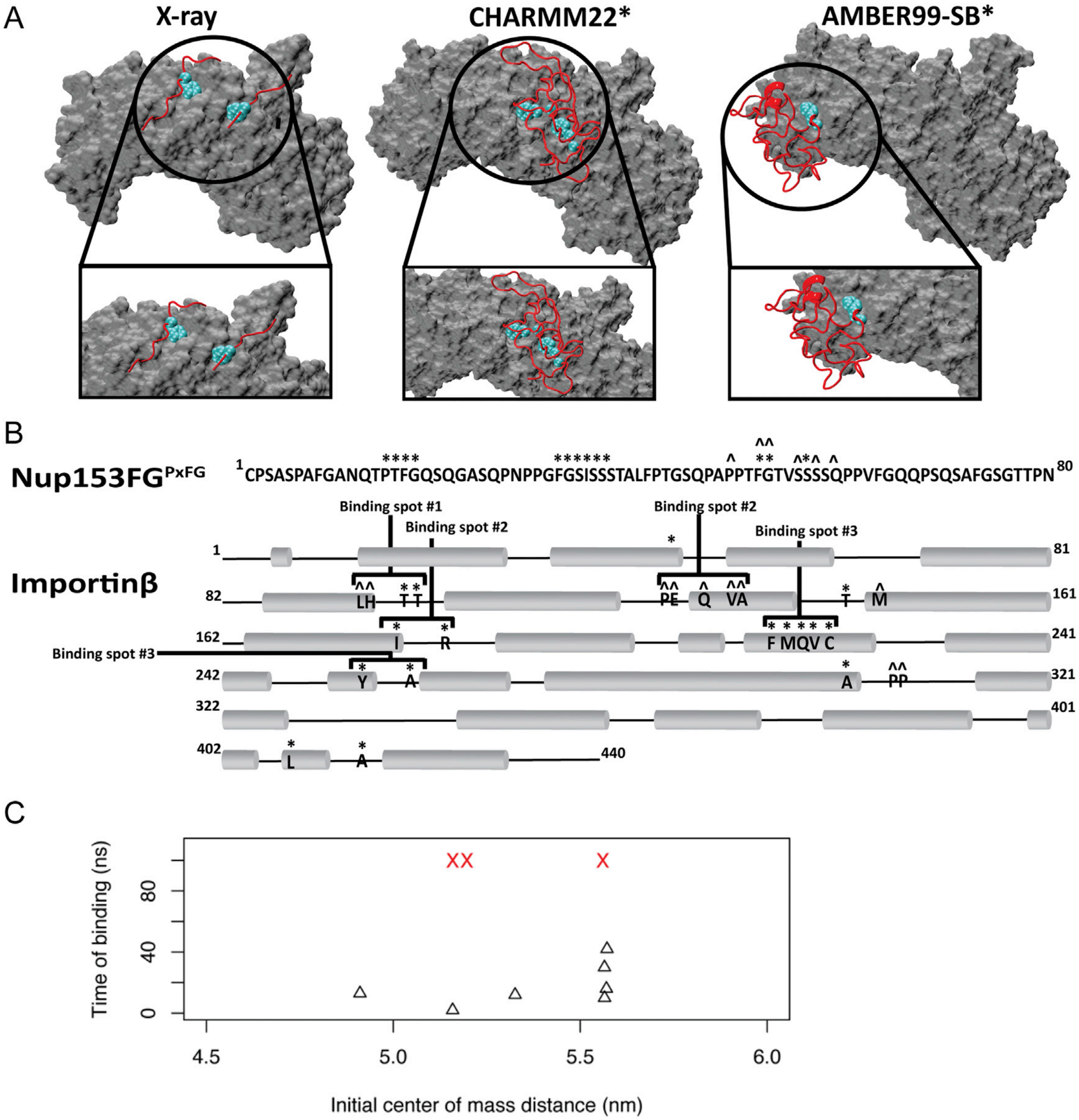

Figure S6. MD Analysis of the Nup153FG^{PxFG}·Importinβ^{N} Complex, Related to Figure 4

(A) Comparison of the Nup153FG^{PxFG}-Importinβ^{N} complexes observed in X-ray crystallographic studies (PDB: 1F59) (left), MD simulations performed using the CHARMM22* (center panel) and the AMBER99-sb*-ILDN (right) force fields. The surface of Importinβ^{N} is shown in gray whereas Nup153FG^{PxFG} is shown in red. FG-repeats are shown in cyan.

(B) Interaction sites of Nup153FG^{PXFG} and Importinβ^{N}. "*" and "^" symbols account for the contacts observed in the CHARMM22* and AMBER99-sb*-ILDN simulations respectively. Grey cylinders represent the α helices along the structure of Importinβ^{N}.

(C) Distance between the center of mass (COM) of Nup153FG^{PxFG} and Importinβ^{N} as a function of the observed time of binding. The upper triangles show simulations reporting successful binding events whereas red crosses show simulations in which binding between the partners has not been observed during the simulated time.

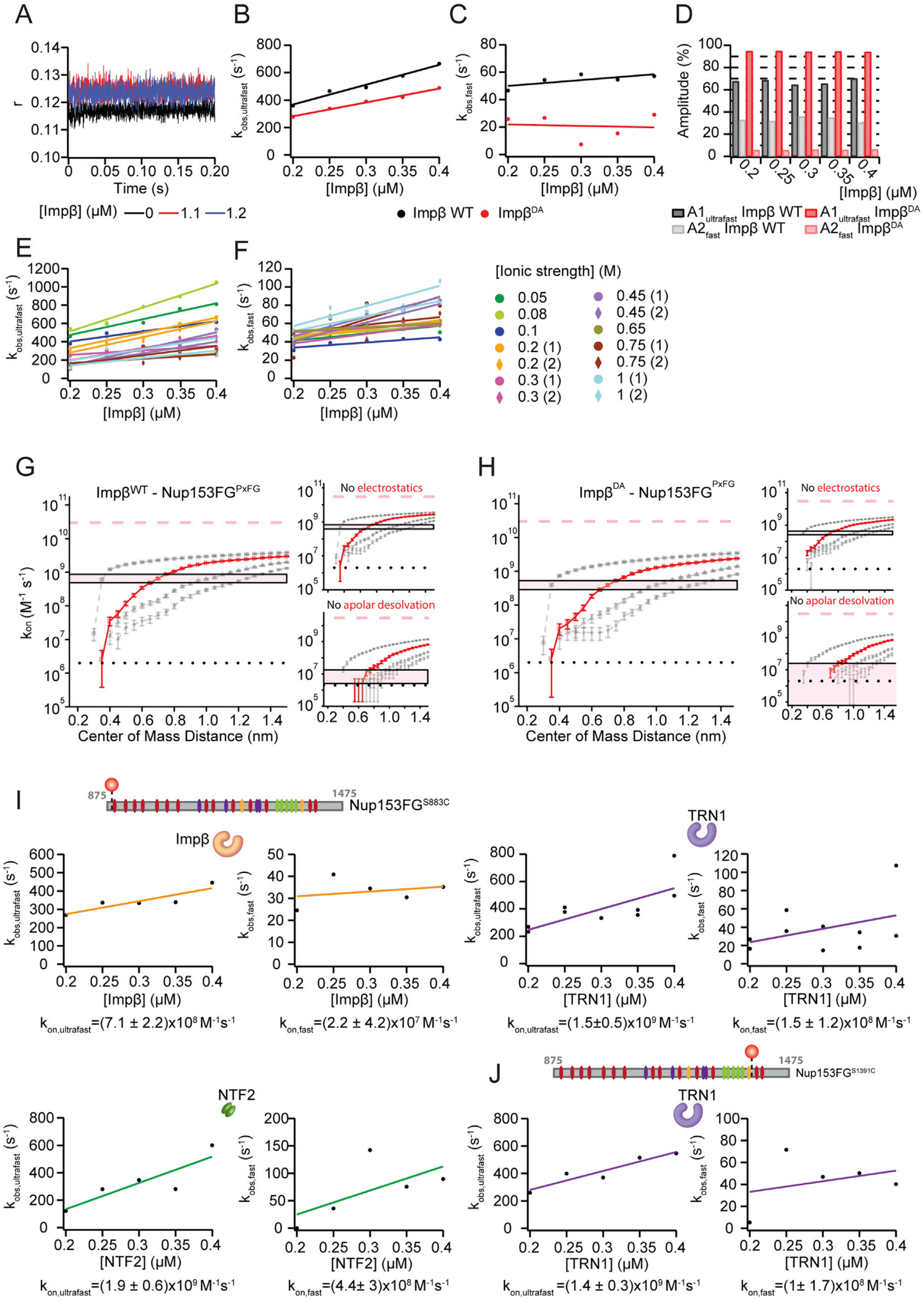

(legend on next page)

Figure S7. Association Stopped-Flow Experiments of Nup153FG and Nup153FG^{PxFG} to NTRs, Related to Figure 5

(A) Association experiment of Nup153FG^{PxFG} with Importinβ WT, a selection of anisotropy traces is shown. At the tested conditions no kinetic changes were detected upon binding.

(B–F) The observed rates from association experiments of Nup153FG to Importinβ WT (black) and double mutant (red) from the major phase ($k_{obs,ultrafast}$) (B) and the minor phase ($k_{obs,fast}$) (C) are plotted against the different Importinβ concentrations. The amplitudes $A1_{ultrafast}$ and $A2_{fast}$ correspond to the amplitudes from the fit associated to $k_{obs,ultrafast}$ and $k_{obs,fast}$ respectively (D). Bars indicating $A1_{ultrafast}$ and $A2_{fast}$ are shown for each measurement at the different concentrations of Importinβ WT and Importinβ^{DA} mutant. Nup153FG and Importinβ WT were also tested at different ionic strength conditions, the observed rates of the ultrafast component ($k_{obs,ultrafast}$) (E) and of the fast component ($k_{obs,fast}$) (F) obtained from different ionic strengths: 0.05 M (dark green), 0.08 M (light green) 0.1 M (blue), 0.2 M (orange),0.3 M (pink), 0.45 M (purple), 0.65 (green khaki), 0.75 M (brown) and 1 M (cyan) are plotted against Importinβ concentration, numbers in brackets correspond to independent replicate measurements. As discussed in detail in (Milles and Lemke, 2014) anisotropy changes in such a disordered system depend on a variety of parameters, including degree of segmental motion and multivalency. As we have several degrees of multivalency (several FG repeats in the Nup, several binding sites in the NTR, and the ability to form higher order complexes than 1:1) the exact origin of the slower component cannot be assessed. In line with related studies a likely origin could be additional binding of NTRs (Wagner et al., 2015). As such FSF measurements report on overall formation of Nup153FG•Importinβ complex i.e., one or more F binding. The fastest k_{on} measured defines even a lower limit for the first F binding. However, we note that both observed reaction rates (major and minor phase) are remarkably fast, and all our conclusions are also valid if one considers only the minor phase.

(G and H) Association rates obtained from Brownian Dynamics simulations. The curves report the estimated k_{on} as a function of the contact distance between the binding partners when a different number of independent contacts (between 1 to 4) are formed between Importinβ^{N} or Importinβ^{N,DA} and Nup153^{PxFG}. The red curves report the cases in which two reaction criteria are satisfied. The condition in which two reaction criteria are satisfied at a distance of 0.7 nm has been used to extrapolate the value of estimated association rate. Pink shaded rectangles show the range of k_{on} when two contacts between 0.65 and 0.75 nm are formed. The simulations were performed considering Nup153FG^{PxFG} interacting with Importinβ^{N} (G) or Importinβ^{N,DA} (H) and considering all interactions contributing to the binding or in the absence of electrostatic interactions or apolar desolvation.

(I and J) Association kinetics for Nup153FG with different NTRs probed by stopped-flow. (I) Here we show the observed rates ($k_{obs,ultrafast}$ and $k_{obs,fast}$ from association experiments with Importinβ (orange), TRN1 (purple) and NTF2 (dark green) to Nup153FG Cy3B labeled at the positions 883C, probing an FxFG enriched region. The obtained association constants ($k_{on,ultrafast}$, $k_{on,fast}$) from the slope of the linear fit are indicated on the plots. (J) Analog to Figure 5, we shown here in addition the observed rates ($k_{obs,ultrafast}$ and $k_{obs,fast}$ from stopped-flow association experiments with TRN1 (purple) to Nup153FG Cy3B labeled at the positions 1391C (probing a PxFG enriched region). The obtained association constants (k_{on}) from the linear fit are indicated on the plots and summarized in Table S2**C**.

We note that the observed rates are at the limit of what is detectable with advanced stopped-flow instrumentation. The signal to noise in the observed anisotropy *r* depends on a variety of parameters, the labeling site, the multivalency, the binding strength, the degree of segmental motion etc. as detailed in (Milles and Lemke, 2014). In line with the observed lower affinity of the used transport receptors for yNup49 (data not shown), signal to noise was not sufficient to perform high resolution stopped-flow measurements of this Nup. Perfectly in line with our results reported in Figure 5, for all those different NTRs and different FG regions we observed remarkably fast rates consistently with a $k_{on,ultrafast} > 5 \cdot 10^{8}\ M^{-1}s^{-1}$. All data are summarized in Table S2C.

Cell

Supplemental Information

Plasticity of an Ultrafast Interaction between Nucleoporins and Nuclear Transport Receptors

Sigrid Milles, Davide Mercadante, Iker Valle Aramburu, Malene Ringkjøbing Jensen, Niccolò Banterle, Christine Koehler, Swati Tyagi, Jane Clarke, Sarah L. Shammas, Martin Blackledge, Frauke Gräter, and Edward A. Lemke

Supplemental Information

Supplemental Experimental Procedures

Sequences of used constructs

Nup153FG (875-1475)

```
        10         20         30         40         50         60
SAKPGTKCGF KGFDTSSSSS NSAASSSFKF GVSSSSSGPS QTLTSTGNFK FGDQGGFKIG
        70         80         90        100        110        120
VSSDSGSINP MSEGFKFSKP IGDFKFGVSS ESKPEEVKKD SKNDNFKFGL SSGLSNPVSL
       130        140        150        160        170        180
TPFQFGVSNL GQEEKKEELP KSSSAGFSFG TGVINSTPAP ANTIVTSENK SSFNLGTIET
       190        200        210        220        230        240
KSASVAPFTA KTSEAKKEEM PATKGGFSFG NVEPASLPSA SVFVLGRTEE KQQEPVTSTS
       250        260        270        280        290        300
LVFGKKADNE EPKAQPVFSF GNSEQTKDEN SSKSTFSFSM TKPSEKESEQ PAKATFAFGA
       310        320        330        340        350        360
QTSTTADQGA AKPVFSFLNN SSSSSSTPAT SAGGGIFGSS TSSSNPPVAT FVFGQSSNPV
       370        380        390        400        410        420
SSSAFGNTAE SSTSQSLLFS QDSKLATTSS TGTAVTPFVF GPGASSNNTT TSGFGFGATT
       430        440        450        460        470        480
TSSSAGSSFV FGTGPSAPSA SPAFGANQTP TFGQSQGASQ PNPPGFGSIS SSTALFPTGS
       490        500        510        520        530        540
QPAPPTFGTV SSSSQPPVFG QQPSQSAFGS GTTPNCSSAF QFGSSTTNFN FTNNSPSGVF
       550        560        570        580        590        600
TFGANSSTPA ASAQPSGSGG FPFNQSPAAF TVGSNGKNVF SSSGTSFSGR KIKTAVRRRK
```

F (total) = 60 # FxFG = 14 # PxFG= 5 # FG= 4 # SAFG= 2

Thick underlined sequence indicates Nup153FG^{PxFG}

Nup153FG^{PxFG} (1313-1390) fragment

(G/G/A)(C/A/C)PSASPAFG ANQTPTFGQS QGASQPNPPG FGSISSSTAL FPTGSQPAPP TFGTVSSSSQ PPVFGQQPSQ SAFGSGTTPN (C/C/AcF)A

F (total) = 7 # FxFG = 0 # PxFG= 5 # FG= 0 # SAFG= 1

The constructs used for the various experiments (NMR/FSF/smFRET) differ as indicated in brackets.

Thin underlined sequence indicates Nup153FG^{FxFG}

Nup153FG^{FxFG} (883-993) fragment

GCGF KGFDTSSSSS NSAASSSFKF GVSSSSSGPS QTLTSTGNFK FGDQGGFKIG VSSDSGSINP MSEGFKFSKP IGDFKFGVSS ESKPEEVKKD SKNDNFKFGL SSGLSNPVA

F (total) = 13 # FxFG = 4 # PxFG= 0 # FG= 0 # SAFG= 0

C labeled Cys

Expression and purification of Nup153FG constructs for smFRET and stopped flow experiments. Unless specified differently, all reagents were purchased from Sigma Aldrich at molecular biology or cell culture grade.

The codon-optimized Nup153FG^{PxFG} (amino acids 1312 to 1391 of the full length Nup153; numbering with respect to the full length protein as in "UniProt: P49790") was cloned into a pTXB3 vector as a 6His Nup153FG^{PxFG} intein chitin binding domain (CBD) construct with a TEV (Tobacco Etch Virus) cleavage site after the His-tag. A TAG stop-codon for Amber suppression and a cysteine were introduced at positions 1391 and 1312, respectively. The protein was recombinantly expressed in *Escherichia coli* (*E. coli*) BL21(AI) (Invitrogen, Carlsbad, CA) harboring the pEvol AcF plasmid for Amber suppression and genetic code expansion in Terrific Broth (TB) medium at 37°C and in the presence of 1 mM p-acetylphenylalanine (AcF) (Milles and Lemke, 2011). Expression was induced at OD_{600} = 1 with 0.02 % arabinose and 1 mM IPTG, and cells were harvested after 4 hours. The protein was purified using standard Ni and Chitin affinity purifications under mild denaturing conditions (phosphate buffered saline (PBS), pH 8, 150 mM NaCl, 2 M urea), intein and 6His-tags were simultaneously cleaved in 20 mM Tris, pH 8 with 100 mM β-mercaptoethanol (BME) and 1 mg of TEV protease per 2 liter expression. Nup153FG^{PxFG} was separated from the His-tagged TEV protease by Ni affinity purification. Fluorescence labeling (maleimide and oxime ligation with Alexa594-maleimide and Alexa488-hydroxylamine (Invitrogen, Carlsbad, CA)) was done as previously described (Milles and Lemke, 2011; Milles et al., 2012), and the protein was separated from free dye by size exclusion chromatography. Nup153FG^{PxFG} was transferred into PBS pH 7.4, 150 mM NaCl, 4 M guanidinium hydrochloride for long-term storage at -80°C. For smFRET measurements, Nup153FG was transferred into smFRET buffer (PBS pH 7.4, 150 mM NaCl, 2 mM DTT, 1 mM $Mg(CH_3COO)_2$).

For fluorescence stopped-flow (FSF) experiments, single cysteine mutant of the full length Nup153FG were labelled analogously with Cy3B maleimide (GE Lifesciences, Pennsylvania).

The full-length Nup153FG was essentially the same as in (Milles and Lemke, 2011). A single cysteine and TAG stop codon for Amber suppression were introduced by site-directed mutagenesis (position 1312 and 1391 respectively).

The mutants FxFG rich region I Nup153FG$^{FxFG(I)}$ (S994TAG,S883C) and FxFG rich region II Nup153FG$^{FxFG(II)}$ (S990C,S1049TAG) were purified and labeled as described above. The same purification was performed with the single cysteine labeled Nup153FG mutants (S1391C and S883C).

Nup153AG was expressed from a pBAD vector in a Nup153AG intein CBD 12His construct. Labeling sites were inserted as for Nup153FG. Expression and purification procedures followed were the same as in (Milles et al., 2013) and labeling of the purified protein was same as for Nup153FG.

Expression and purification of yNup49FG for smFRET. The yNup49FG (amino acids 1-260, numbering with respect to the full length protein "UniProt: Q02199") was expressed from a

pBAD-6His-Intein vector. This construct contains a C at residue position 250. The mutation A191TAG was introduced in addition to permit FRET labelling as described above for the Nup153FG proteins. Purification was performed using standard Ni affinity purification procedure with mild denaturing conditions (phosphate buffered saline (PBS), pH 8, 150 mM NaCl, 2 M urea). 6His-tag was cleaved by incubating the sample with 100 mM β-mercaptoethanol (BME) overnight at room temperature. After buffer exchange a second Ni affinity purification was performed and after labeling the cleaved yNup49FG was run over size exclusion chromatography.

Expression and purification of Nup153FG for NMR experiments. The same Nup153FG^{PxFG} construct as for fluorescence measurements, but with a Cys instead of AcF was transformed in *E.coli* BL21 (DE3) and grown in Lysogeny broth (LB) medium at 37°C at an OD_{600} of 0.8. The culture was harvested and transferred into M9-AC medium containing 1 g/l $^{15}NH_4Cl$ and 2 g/l glucose (^{13}C labeled if needed). Growth was continued for 1 h at 20°C until expression was induced by addition of 1 mM IPTG for 12 to 14 h. Purification of Nup153FG^{PxFG} was done essentially as for fluorescence experiments in 50 mM Tris pH 8, 150 mM NaCl. As a last step, Nup153FG^{PxFG} was transferred into NMR buffer (50 mM Na-phosphate pH 6, 150 mM NaCl, 5 mM $MgCl_2$, 2 mM dithiothreitol (DTT)) by size exclusion chromatography.
^{15}N labeled Nup153AG$^{PxAG,\ F1374}$ was cloned the same way as Nup153FG^{PxFG}, but all F except F1374 were replaced by A. Expression and purification were analogous to Nup153FG^{PxFG}.

Expression and purification of Importinβ. Importinβ was expressed and purified essentially as described (Milles and Lemke, 2014). Briefly, an intein-6His construct in a pTXB3 vector was transformed in *E.coli* BL21 DE3(AI). Cultures were grown in TB or LB medium at 37°C until OD_{600} = 0.6, induced with 0.02 % arabinose and 1 mM IPTG, and growth was continued at 30°C for 4 hours or 20°C for 12 to 14 hours. Purification was done following standard Ni affinity protocols in 50 mM Tris pH 7.0, 650 mM NaCl, 5 mM $MgCl_2$. 0.2 mM TCEP (tris(2-carboxyethyl)phosphine). Intein cleavage was done overnight at room temperature using 100 mM BME, and the tag was removed by an additional Ni-affinity step. The protein was concentrated and submitted to size-exclusion chromatography. Importinβ was then transferred into NMR or smFRET buffer.

Expression and purification of Transportin1. Transportin1 (TRN1) was expressed in *E.coli* XL10 Gold cells transformed with pQE60-Transportin1-6His. Protein expression was induced at OD_{600}=0.6 with 1mM IPTG and the cultures were grown for 6 hours at 30 ˚C. Harvested cells were lysed in 50 mM Tris pH 8, 500 mM NaCl, 5 mM $MgCl_2$, 1 mM PMSF and 0.2 mM TCEP. The lysate was incubated with Ni-beads after centrifugation and a 1.5 M NaCl wash was performed before elution. The eluted protein was then run over size exclusion chromatography.

Expression and purification of NTF2. NTF2 was expressed in *E.coli* BL21 (DE3) transformed

with a pTXB3-NTF2-Intein-6His plasmid. Expression was induced with 1 mM IPTG at OD_{600}=0.6 and cells were incubated overnight at 34 ˚C. Harvested cells were lysed with 50 mM Tris pH 8, 500 mM NaCl, 5 mM $MgCl_2$, 5 mM imidazol, 1 mM PMSF and 0.2 mM TCEP. Then the clear lysate was incubated with Ni-beads. The elution from the Ni-beads was incubated with 100 mM BME overnight at room temperature, in the presence of Complete EDTA (Roche) free protease inhibitors. After buffer exchange the sample was incubated again on Ni-beads and the untagged NTF2 was run over size exclusion chromatography.

Expression and purification of CRM1. CRM1 was expressed on *E.coli* TG1 cells transformed with a pQE60-CRM1-Intein-12His vector. Protein expression was performed in TB medium. Induction was done at OD_{600}=0.8 with 1 mM IPTG and the cultures were incubated at 18 ˚C overnight. The harvested cells were lysed with 50 mM Tris pH 8, 650 mM NaCl, 5 mM imidazol, 1 mM PMSF and 0.2 mM TCEP. The clear lysate after centrifugation was then incubated with Ni beads following standard protocols. The elution from the Ni-beads was incubated with 100 mM BME overnight at room temperature to cleave the intein-12His-tag in the presence of Complete EDTA free protease inhibitors. Then the sample was buffer exchanged to perform a second incubation with Ni-beads. The obtained cleaved protein was then run over size exclusion chromatography.

Spectral assignment of Nup153FG^{PxFG} and RDC measurements. Spectral assignments of ^{13}C, ^{15}N Nup153FG^{PxFG} were obtained from a set of BEST-TROSY-type triple resonance spectra: HNCO, intra-residue HN(CA)CO, HN(CO)CA, intra-residue HNCA, HN(COCA)CB, and intra-residue HN(CA)CB (Solyom et al., 2013). The spectra were processed with NMRPipe (Delaglio et al., 1995) and automatic assignment was done with the program MARS (Jung and Zweckstetter, 2004), followed by manual verification. Secondary chemical shifts were calculated using the random coil values from refDB (Zhang et al., 2003).
For the measurements of RDCs, ^{13}C, ^{15}N Nup153FG^{PxFG} was aligned in Pf1 phages to yield a D_2O splitting of 2.16 Hz. RDCs were measured using BEST-type HNCO and HN(CO)CA experiments allowing for spin-coupling measurements in the ^{13}C dimension (Rasia et al., 2011). All experiments were performed in NMR buffer, at 25°C and at a ^{1}H frequency of 600 MHz.

Ensemble description of Nup153FG^{PxFG}. A statistical coil ensemble of Nup153FG^{PxFG} comprising 10,000 structures was generated using Flexible-Meccano. The genetic algorithm ASTEROIDS was used to select five times 200 conformations that best described the experimentally obtained chemical shifts (Jensen et al., 2010). Based on the ϕ and ψ angles of the selected conformers, a new ensemble of 8,500 conformers was generated, supplemented with 1500 structures from the initial ensemble, and subjected to another round of ASTEROIDS selection. This iteration step was repeated four times as described previously (Ozenne et al., 2012) until convergence was achieved with respect to the experimental chemical shifts.
Based on the selected ensembles (5 times 200 structures) from the fourth iteration, 10,000

conformers were again generated using Flexible-Meccano, from which $^{1}D_{N\text{-}NH}$, $^{1}D_{C\alpha\text{-}H\alpha}$ RDCs were calculated and compared to the experimentally obtained values.
Ensemble averaged chemical shifts were obtained using SPARTA (Shen and Bax, 2007), RDCs were calculated using PALES (Zweckstetter et al., 2004) and SAXS curves were calculated using CRYSOL (Svergun et al., 1995).

^{15}N relaxation and relaxation dispersion of Nup153FG^{PxFG} and Nup153AG$^{PxAG,\ F1374}$. ^{15}N R_1 and $R_{1\rho}$ experiments and {^{1}H}-^{15}N heteronuclear Overhauser effetcs (nOe) were carried out at a ^{1}H frequency of 600 MHz and at a temperature of 25°C. The ^{15}N R_1 relaxation rates were obtained by sampling the decay of magnetisation at 0, 0.08, 0.19, 0.38, 0.57, 0.76, 0.99, 1.44, and 1.71 s. The time point for a delay time of 0.57 s was repeated for error estimation. $R_{1\rho}$ relaxation rates were determined using delay times of 0.001, 0.02, 0.05, 0.08, 0.1, 0.15, 0.2, and 0.25 s, with a repetition at a delay time of 0.25 s for the interaction with Importinβ and the nucleoporin alone. Interactions with TRN1 and NTF2 were measured with time delays 0, 0.076, 0.304, 0.57, 0.874, 1.52 s with a repetition at delay time 0.57 s for R_1 and 0.001, 0.02, 0.05, 0.1, 0.17, and 0.25 s with a repetition at delay time 0.05 s for $R_{1\rho}$. The spin-lock field was 1500 Hz. R2 was calculated based on R1 and R1ρ according to ref. (Akke and Palmer, 1996).

$$R_{1\rho} = R_1 \cos^2(\theta) + R_2 \sin^2(\theta)$$

with $\theta = \arctan(\frac{\gamma_N B_1}{\Delta\upsilon \cdot 2\pi})$.
^{15}N relaxation dispersion was carried out at a Nup153FG^{PxFG} concentration of 250 μM and an Importinβ concentration of 180 μM (Hansen et al., 2008). 22 points, including one duplicate, were recorded at CPMG frequencies between 25 and 1,000 Hz using constant-time relaxation delay of 40 ms.

Calculation of residue-specific apparent K_d values from ^{15}N relaxation. R_2 from every F in the Nup153FG^{PxFG} and Nup153AG$^{PxAG,\ F1374}$ sequence was extracted for all the experiments at different Importinβ concentrations. The observed R_2 can be described as the weighted average between R_2 in the bound (R_2^{NI}) and unbound (R_2^{N}) form of Nup153FG according to

$$R_2 = c \cdot R_2^{NI} + (1-c) \cdot R_2^{N}$$

c describes the concentration ratio of the complex with respect to the total nucleoporin concentration as follows:

$$c = \frac{[NI]}{N_{tot}}$$

This ratio contains the dissociation coefficient (K_d) according to $K_d = \frac{[N]\cdot[I]}{[NI]}$, and

$[I] = [I_{tot}] - [NI]$, resulting in $[NI] = \frac{[N]\cdot[I_{tot}]}{K_d + [N]}$.

With the rotation time of Importinβ as measured by fluorescence correlation spectroscopy (Milles and Lemke, 2014), we can calculate the expected R_2^I for Importinβ assuming isotropic rotation as a spherical molecule (48 s^{-1} at 600MHz if only dipolar relaxation is considered). We can then further assume that the local correlation time (τ_c) of the FG repeats of Nup153FG when bound to Importinβ is equal to the correlation time of isolated Importinβ ($R_2^{NI} \approx R_2^I$).
Under the assumption of [N] = [N_{tot}], i.e. that only a small fraction of the nucleoporin is bound, we obtain the following expression for the observed R_2:

$$R_2 = \frac{[I_{tot}]}{(K_d + [N_{tot}])}(R_2^I - R_2^N) + R_2^N .$$

When we calculate the ratio of bound nucleoporin compared to the total nucleoporin concentration according to

$$\frac{[NI]}{[N_{tot}]} = c = 1 - \frac{R_2 - R_2^N}{R_2^N - R_2^{NI}}$$

at the highest Importinβ concentration employed and for the largest R_2 measured within an FG repeat, we obtain a fraction of bound Nup153 of lower than 17%.
The observed R_2 rates were then plotted against Importinβ concentration (Nup concentration remained constant at 250 μM), and a linear fit was obtained from these plots for every F, and the K_d was extracted from the slope.
Note that this calculation implicitly assumes that all F of Nup153FG^{PxFG} are free to bind even if one (or more) F of the same molecule are already bound to Importinβ, and that binding of Importinβ does not reduce the effectively available Importinβ concentration. Several FG binding sites are known on Importinβ (Bayliss et al., 2000; Bednenko et al., 2003; Isgro and Schulten, 2005; Otsuka et al., 2008), and we have shown that multiple Importinβ molecules can likely bind the full Nup153FG at the same time (Milles and Lemke, 2014).
The same protocol was applied to extract residue specific K_d values for TRN1 binding. Since Importinβ and TRN1 have approximately the same size and similar structures, we assumed the same rotation time as for Importinβ. Nup153FG^{PxFG} concentration remained constant at 250 μM and the TRN1/Nup153FG^{PxFG} molar ratio was varied according to 0.61, 0.33, and 0.17. While the same relaxation experiments were performed with NTF2, no experimental rotational correlation time of the complex was available and since NTF2 is very small compared to Importinβ and TRN1, the assumption of equal rotation in the bound and unbound form likely does not hold. Site-specific K_d values were therefore extracted from chemical shift changes.

Calculation of residue-specific apparent K_d values from chemical shift changes. ^{1}H-^{15}N HSQC spectra were measured at a ^{1}H frequency of 850 MHz and 25°C with a Nup153FG^{PxFG} concentration of 80 μM and varying NTF2 concentrations (6-, 12-, 18-, and 24-fold excess of NTF2). 36- and 48-fold excess of NTF2 was measured at a Nup concentration of 60 and 40 μM respectively, as only lowering the Nup concentration allowed these NTF2/Nup ratios. Chemical shifts for all F (except F1327, which was excluded due to spectral overlap), were extracted and

absolute chemical shift changes were calculated according to $\Delta = \sqrt{(\delta^{15}N)^2 + (\delta^1 H \cdot 6.5)^2}$, with $\delta^{15}N$ and $\delta^1 H$ the ^{15}N and ^{1}H chemical shift changes with respect to the unbound Nup153FG^{PxFG}. Δ was then plotted against the molar ratio between NTF2 and Nup153FG^{PxFG} and fit with a simple binding model under the assumption of excess NTF2 ($\Delta = \frac{\Delta_{\max} \cdot B}{K_d + B}; B = \frac{NTF2}{Nup}$). Errors were extracted from the fit.

SmFRET experiments and protein diffusion. SmFRET measurements and the experimental setup for the measurements have previously been described (Milles and Lemke, 2011; Milles et al., 2012). The different FRET labelled Nup153FG constructs were provided at a concentration of ~ 50 pM and Importinβ, TRN1, NTF2 and CRM1 were added at 1 µM concentration. In the case of yeast Nup49 (aa 1-260) smFRET measurements were performed with 10 µM NTR concentration. Single molecule fluorescence lifetimes were calculated using a maximum likelihood estimator (Schaffer et al., 1999). E_{FRET} values were corrected for donor leakage into the acceptor channel, direct excitation of the acceptor by the 483 nm laser and intensity differences in the green (donor, I_{Do})/red (acceptor, I_{Ac}) channels due to differences in detection and fluorescence quantum yields (γ) (Ferreon et al., 2009):

$$E_{FRET} = \frac{I_{Ac}}{I_{Ac} + \gamma \cdot I_{Do}}$$

Measurements were acquired with interleaved donor and acceptor excitation at a pulse rate of 27 MHz per color. For analysis of ensemble averaged lifetime histograms, E_{FRET} versus stoichiometry (S) histograms with

$$S = \frac{I_{Do} + I_{Ac}}{I_{Do} + I_{Ac} + I_{Ac}^{PIE}} ,$$

With I_{Ac}^{PIE} being the acceptor fluorescence upon acceptor excitation, were generated. Fluorescence lifetime histograms were built from either all photons resulting from bursts that contained double labeled molecules, or all photons resulting from donor only containing bursts. Accumulated fluorescence lifetimes from single molecule events were fit with a simple mono-exponential decay convolved with the impulse response function.
Fluorescence correlation spectroscopy (FCS) experiments were performed on a custom built confocal setup. Linearly polarized laser light was used to excite the samples. Detection of fluorescence signal was performed in parallel and perpendicular polarization directions and then the correlation curve was obtained by cross correlation. The curves were normalized for comparison purposes. The titration experiments with different Importinβ concentrations were performed with Cy3B single labeled proteins. FCS of double labeled proteins was measured in order to test binding of different NTRs to the proteins used in smFRET experiments.

Fluorescence stopped-flow (FSF) experiments. The association kinetics were monitored by following the anisotropy change of Nup153FG^{S1391C} labeled with Cy3B at the position 1391C

(near the PxFG region) and of Nup153FG^{S883C} labeled with Cy3B at the position 883C (FxFG motif enriched region, see sequences) upon binding to different NTRs at different concentrations, under pseudo-first order conditions, using stopped-flow spectroscopy (SFM-3000, Bio-logic) with the uFC-08 micro-cuvette accessory. Excitation was performed with a custom polarized LASER excitation source 532nm and polarized emission was detected using emission filters with 538-642nm bandwidth. Fluorescence intensities were measured with polarizing filters in parallel (‖) and perpendicular (⊥) position. The anisotropy (r) was calculated following:

$$r = \frac{I_{\parallel} + G \cdot I_{\perp}}{I_{\parallel} + 2G \cdot I_{\perp}}$$

Measurements were performed using the automatic mixing function (concentration dependent study) provided by the software (Bio-Kine32 V.4.72). 20 nM Nup153FG-Cy3B were mixed with the different NTRs at 200-400 nM in 50 nM steps. All the measurements were performed at 20 ˚C. Each trace was obtained by averaging 30 traces and background fluorescence subtracted. The anisotropy traces where fit to:

$$f(t) = A1 \cdot (1 - e^{-k1_{obs} \cdot t}) + A2 \cdot (1 - e^{-k2_{obs} \cdot t}) + c$$

The different k_{obs} were plotted against the respective Importinβ concentrations and were linearly fit to obtain the corresponding association constant (k_{on}). The estimation of the equilibrium dissociation constant (K_d) was obtained from the final anisotropy value of measurements by fitting it to:

$$r = \frac{[\mathrm{Imp}\beta]}{[\mathrm{Imp}\beta] + K_d} \cdot (r_B - r_F) + r_F$$

where r_B and r_F correspond to the anisotropy of free and bound Nup153FG-Cy3B and [Impβ] is the Importinβ concentration.

Effect of electrostatics as assessed by FSF experiments. The stopped-flow measurements at different ionic strength were performed at 20 mM MOPS pH 7.4 with various NaCl concentrations at 20˚C. MOPS buffer was used because it buffers in the same range as PBS but enables to lower the ionic strength. In **Figure 5**, The $k_{on,ultrafast}$ obtained from the fit of the association experiment between Nup153FG and Importinβ WT were plotted against the corresponding ionic strength. Points which were out of a three standard deviation confidence band were treated as outliers. The basal association rate constant was obtained by fitting the data to a Debye-Huckel-like approximation as used in ref.(Shammas et al., 2014).

$$\ln(k_{on}) = \ln(k_{on,basal}) + \frac{AB}{BR} \cdot \frac{I^{-0.5}}{BR + I^{-0.5}}$$

where I is the ionic strength of the buffer and AB and BR are used as free fitting parameters.

Error in FSF experiments. The BioLogic stopped flow equipment permits automatic titration and repeated technical replicates, which typically yield a small standard deviation. However, for k_{on} measurements of such complex systems, several experimental parameters are important,

especially the exact concentration and purity of proteins. We thus performed over several months repeatedly the same measurements from different protein expression batches. From this, we derived an experimental error of ~20% in k_{on} measurements between different replicates. To be conservative, we thus do not show (the typically lower) standard deviations from technical replicates.

Estimation of the theoretical diffusion-limited rate constant. The diffusion-limited binding rate constant for bimolecular reactions can be calculated by substituting the Stokes-Einstein relations into the Smoluchowski diffusion-limited equation:

$$k_{on} = 4\pi DR = N_A \frac{2k_B T}{3\eta}\left(\frac{D_A}{D_B} + \frac{D_B}{D_A} + 2\right)$$

Where η is the viscosity of the solution, k_B the Boltzmann constant, T the temperature, D_A and D_B correspond with the diffusion coefficient of Importinβ and Nup153FG. FCS was performed to determine the diffusion coefficients (D) of Nup153FG 1391C, Nup153FG^{PxFG} and Importinβ labeled with Atto655. The D obtained were $3.83\cdot10^{-5}$, $1.42\cdot10^{-5}$ and $2.34\cdot10^{-5}$ cm^2 s^{-1} respectively. The estimated diffusion-limited association rate constant for Importinβ and Nup153FG 1391C was of $6.8\cdot10^9$ $M^{-1}s^{-1}$ and for Importinβ and Nup153FG^{PXFG} of $6.81\cdot10^9$ $M^{-1}s^{-1}$. These values are within the accepted range of 10^9-10^{10} $M^{-1}s^{-1}$ for diffusion-limited reactions. In BD simulations, a truncated Importinβ^N was used, which would have a higher diffusion coefficient and thus also a slightly higher theoretical k_{on}. Overall, the changes in k_{on} due to different MW of the proteins in BD and FSF experiments are low (~ 6 fold in molecular weight), and were thus not corrected for (see experimental error discussion for FSF experiments).

Limitations of SFS measurements. Stopped flow experiments of Nup153FG^{PxFG} with Importinβ did not show any appreciable signal change in anisotropy (**Figure S7)**, due to the much lower affinity of this short construct to the NTR. Thus, we measured the full-length Nup153FG, which has a much higher affinity and yielded appreciable signal changes. The anisotropy changes are for most conditions best fit by a biexponential decay. One rate lies typically in the order of $k_{on,ultrafast}$ ~ 10^9 $M^{-1}s^{-1}$, while the second one is around one order of magnitude lower $k_{on,\ fast}$ ~ 10^8 $M^{-1}s^{-1}$. The amplitude of the second component decreases with increasing Importinβ concentration, so that the first component is the dominating species.

It is important to note that we monitor the interaction of two large highly multivalent proteins; Nup153 has 25 FG repeats, and Importinβ has been suggested to have between 4- 16 FG binding sites (Isgro and Schulten, 2005; Otsuka et al., 2008). Previous studies also suggested that Importinβ binds preferentially to different regions in the Nup153FG, and that we can expect up to 8 Importinβ molecules to bind to a single Nup153FG (Milles and Lemke, 2014). The multiple components most likely indicate primary binding and then slower secondary binding events of Importinβ molecules, which would be perfectly in line with observations made for multiple Importinβ binding to immobilized layers of Nup153 by (Schoch et al., 2012; Wagner et al., 2015). While we cannot pinpoint the exact origin of the two components, both are very fast, and

support all conclusions drawn in the paper. Crucially we are interested in how fast the first F binds, and as we probe entire segments and not just single F's in the anisotropy measurements (Milles and Lemke, 2014) the highest observed rate still defines a lower limit for this.

***k_{off}* estimates.** Note that the error for the intercept of the SFS measurement shown in **Figure 5** is very high. In principle from this a $k_{off,SFS}$ can be determined, and we report those also in **Table S2A**. Another way to get an estimate of k_{off} is via $K_{d,app} = k_{off,global}/k_{on,SFS}$. Due to the nature of our anisotropy measurement, we only detect local binding near the dye, and amplitude and rise of the concentration dependent binding curve can be affected by different multivalent effects (see (Milles and Lemke, 2014) for a detailed discussion). However, an apparent $K_{d,app}$ can still be extracted, which is useful for relative comparisons, and can be used to calculate a $k_{off,global}$. $k_{off,global}$ and $k_{off,SFS}$ turned out to be on the same order of magnitude for Importinβ measurements. Since the error in $k_{off,global}$ is much lower it is the preferred value used in the text.

Toy models to estimate site specific $k_{off,single}$ in a multivalent system. The polyvalency/multivalency within the system we have described is extensive and therefore problematic to model. However to illustrate its importance in determining dissociation rates for Nup153 molecules we consider two separate toy models.

We first consider as a limiting case a simple bivalent system. The two molecules are only allowed to separate if the neighbouring motif is also unbound when the dissociation event occurs. The probability of this is related to the affinity of that site and the effective concentration (given that its neighbor is bound nearby). According to Kramer and Karpen this reduces the overall dissociation rate constant according to (Kramer and Karpen, 1998):

$$k_{off,\text{dimer}} = k_{off,\text{single}} \frac{2K_d}{K_d + C_{eff}}$$

where K_d is the equilibrium constant for an individual site and C_{eff} is the local concentration of binding sites. As the full length Nup153FG domain has 25 FG binding sites (and 60 F in total), one can expect that in such a system the $k_{off,single}$ for each individual site would be related to the overall (measured) dissociation rate as follows:

$$k_{off,\text{single}} >> k_{off,globalSFS} \left(\frac{K_d + C_{eff,SFS}}{2K_d}\right)$$

The average measured $K_{d,NMR}$ across all sites in Nup153FG^{PxFG} as measured by NMR (**Figure 3**) is ~ 2mM, and we use this figure to represent K_d. We can then combine this value with an estimate of the effective concentration of sites to provide a lower bound for $k_{off,single}$. Based upon previously reported dimensions of intrinsically disordered proteins (Bernado and Blackledge, 2009) the effective concentrations of FG motifs and F motifs within each Nup153 molecule are approximately 40 mM and 90 mM respectively. Using these figures we conclude that $k_{off,single}$ 10^3 s^{-1}.

Interestingly we note that since the stoichiometry and dimension of the NPC are known, the

concentration of FG repeats within the NPC has already been estimated to be 50 mM (Bui et al., 2013; Frey and Gorlich, 2007; Ori et al., 2013).

We then obtain an estimate of the dissociation rate by taking an entirely separate approach (toy model 2) and considering the association rates. For the simplest case, where only a single F motif is contained within the shorter Nup construct, the interaction is bimolecular, and we can apply k_{off}(F1374) = k_{on}(F1374)•K_d(F1374) to create an estimate of $k_{off,single}$. The equilibrium constant is known from our NMR measurements, $K_{d,NMR}$(F1374)= 8 mM (Figure 3). For practical reasons it is not possible for us to determine k_{on}(F1374) experimentally, however we form an approximation for informative purposes. For diffusion-limited reactions where the entire surface is considered equally reactive the protein size is expected to make very limited difference to the reaction rate as a result of counteracting effects of diffusion rates and target area so we approximate $k_{on,global\ PxFG} \sim k_{on,global\ SFS}$. However for Nup153AG$^{PxAG,\ F1374}$ we anticipate that the achieved association rate will be lower than $k_{on,global,PxFG}$ (and therefore $k_{on,global\ SFS}$) because mutating all but one of the 8 identified binding sites leads to a roughly 8-fold lower proportionate "reactive" surface of the protein and so fewer collisions will be correctly aligned. To a first approximation therefore $k_{on,NMR}$(F1374)= $k_{on(global,SFS)}$/8. This leads to an estimate of $k_{off,single}$ as k_{off}(F1374) = k_{on}(F1374)•K_d(F1374) ~ 10^6 s^{-1}, corresponding to an unbinding time (UT) of ~1 μs.
We stress that both models are based on assumptions as we currently lack a quantitative understanding of such complex mechanisms. Nevertheless, they point to a substantially higher $k_{off,single}$ for a single site compared to the global k_{off} recovered from SFS measurements.

Bulk transport experiment. *In vitro* reconstitution of the nucleocytoplasmic transport was achieved as previously described (Kose et al., 2015). In brief cells were washed 3x with transport buffer (20 mM Hepes, 110 mM KOAc, 5 mM NaOAc, 2 mM MgOAc, 1 mM EGTA, pH 7.3) and incubated with Hoechst33342 (1μg/mL in PBS) for 15 min. Cells were then permeabilized via incubation on ice for 10 min with digitonin (40 μg/mL), washed 3 times with transport buffer supplied with 1.5% polyvinylpyrrolidone (PVP, 360 kDa) to remove the soluble factors and digitonin before the addition of the transport mix. The transport mix consists of the purified NTRs, recycling factors and a fluorescent cargo (NLS-MBP-eGFP) with the addition of a source of energy (1 μM Importinβ, 1 μM Importinα, 4 μM Ran, 2 μM NTF2, 0.5 μM NLS-MBP-eGFP, 2 mM GTP, 1 mM DTT in transport buffer). For negative controls cells were incubated for 15 min at RT with the lectin Wheat Germ Agglutinin (WGA)(500 μg/mL) which inhibits nuclear transport before addition of the transport mix (Finlay et al., 1987). Fluorescent eGFP signal was then imaged on a commercial confocal microscope (Leica SP8, Leica Microsystems) directly after the transport mix addition for up to 1 hour.

Single molecule tracking and experimental setup. Single molecule (sm) measurements were performed on a custom made experimental setup built around a 100x UAPON Olympus TIRFM

high N.A. objective. A 488 (150 mW LuxX, Omicron Laser, Germany) and a 568 (100 mW Sapphire, Coherent, US) were combined (using LASER dichroics from AHF, Tubingen, Germany) and focused at the back plane of the objective. Collected fluorescent light was then separated with a custom dichroic mirror and split on two high sensitivity EMCCD cameras for the green and red fluorescent channel (iXon3 and iXonUltra, Andor, Ireland).

Time lapse image series of fluorescently labeled Importinβ-Alexa488 were acquired with an acquisition time of 2 ms in presence of transport mix. The transport mix is the same described above except that the cargo was, is this case, a NLS-MBP-mCherry and that instead of unlabeled Importinβ, 15 nM Importinβ-Alexa647 and sm concentration (~pM) of Importinβ-Alexa488 were added. Fluorescently labeled Importinβ-Alexa488 positions were then detected (based on a Maximum Likelihood Ratio) in each frame and localized (fitting with a symmetrical 2D Gaussian function) with the Localizer Package (Dedecker et al., 2012) for IgorPro (Wavemetrics). Tracks were then reconstructed with the nearest neighbor algorithm implemented in the Localizer Package for IgorPro: if two particles appeared within a distance of less than 400 nm and 3 frames (6 ms) the particles were connected into a trajectory. To select the trajectories corresponding to particles interacting with the NPC, the position of the latter was determined starting from an ensemble image of Importinβ labeled with Alexa594. Since NPCs appear as diffraction limited spots, their position was detected with the same methodology adopted for single particles. The nuclear envelope shape was then reconstructed by fitting a 2D spline to NPCs position. For the analysis tracks were then selected for crossing events, i.e. requiring the initial and the final detected position in the trajectory to be on opposite side of the fitted NE (respectively outside and inside of the nucleus) at a minimum distance of 70 nm from the NE itself.

MD simulations, Nup153FG^{PxFG} unbound. Nup153FG^{PxFG} fragment was modeled on the base of its sequence that also included the exogenously inserted residues used for the labeling of the fragment with fluorophores. After energy minimization of the structure, the fragment was still partially stretched. The fragment was simulated using CHARMM22*(Lindorff-Larsen et al., 2010) and AMBER99-SB*(Piana et al., 2011) forcefields after being placed in a box of TIP3P water molecules (Jorgensen et al., 1983; Pronk et al., 2013) and 100 mM of Na^+ and Cl^- ions having dimensions 7.113 x 6.79 x 4.98 nm^3. After solvation, the solvent was equilibrated for 100 ps during which the atoms of the protein were kept at the energy minimized Cartesian coordinates by a spring constant of 1000 kJ mol^{-1} nm^{-2}. Equilibration of the system was achieved through two phases, first a canonical ensemble (NVT) phase (isochoric environment) in which the velocities for each particle were generated and a V-rescale thermostat was used to couple the temperature of the system, then a second isobaric-isothermal ensemble (NPT) phase, in which pressure was coupled using the Parrinello-Rahaman barostat (Teraoka, 2002). After equilibration, molecular dynamics trajectories were recorded for 150 ns in 15 independent simulations, for which the initial sets of particle velocities were different. Based on a root mean squared deviation (RMSD) cutoff, we performed a clustering analysis of the conformational ensemble

explored by the Nup153FG^{PxFG} and, for each force field, two conformations representing central structures of the most numerously populated clusters were picked randomly to perform the MD simulations in presence of Importinβ^{N}. The clustering analysis was carried out after concatenating the trajectories collected for all the simulations and choosing a RMSD cutoff of 0.1 nm for the backbone+Cβ atoms of the protein. All simulations were performed using the GROMACS 4.5.5 package (Pronk et al., 2013).
We note that the Nup153FG^{PxFG} conformers exhibit an average R_g below the experimental values (Mercadante et al., 2015), owing to the well-known tendency of canonical force fields including AMBER99-sb*-ILDN and CHARMM22* to favor globe-like structures (Lindorff-Larsen et al., 2010; Mercadante et al., 2015; Piana et al., 2015) and to the lack of net charged amino acids along the sequence of the simulated Nup153^{PxFG}.

MD simulations, Nup153FG^{FxFG} unbound. The N-terminal fragment of Nup153FG comprising residues 883 to 993 and named as Nup153FG^{FxFG} (FxFG rich region I, see sequences) was simulated similarly to the Nup153FG^{PxFG} fragment described above. With respect to the simulations sampling the dynamics of the Nup153FG^{PxFG} the simulation box used to sample the dynamics of the Nup153FG^{FxFG} was, however, bigger (14.256 x 12.798 x 6.926 nm^3) in order to contain such a longer fragment, which is composed, in this case, of 114 residues. Water model and all the other parameters used to run MD simulations were adapted from the previous simulations ran on Nup153FG^{PxFG}.

MD simulations Nup153FG^{PxFG} in the presence of Importinβ^{N}. Simulations were set up choosing two conformations of Nup153FG^{PxFG} as described above and were performed using the same setup described for unbound Nup153FG^{PxFG}. Nup153FG^{PxFG} was randomly placed in a box of dimensions 15 x 15 x 15 nm^3 together with the N-terminal segment of Importinβ ("PDB: 1F59" (Bayliss et al., 2000)). The choice of selecting only the N-terminal half of Importinβ for the MD simulations is justified by the observation that Importinβ is modularly built and the C-terminal end is highly similar to the N-terminal end (Bayliss et al., 2000). Indeed, several FG-Nups binding pockets at the C-terminal end of Importinβ have been predicted (and confirmed by computational and experimental studies) through a simple sequence alignment and structural modeling of the C-terminal using the binding pockets identified on the N-terminal part of the protein as a template (Isgro and Schulten, 2005). The initial positions of Nup153FG^{PxFG} with respect to Importinβ were chosen randomly, so that the binding would not be biased by the initial orientation or distance to the binding partners. Five independent 100 ns-long simulations were performed on each system for a total of 20 simulations (5 simulations for each of the two most numerous clusters using both force fields, AMBER99-SB* and CHARMM22*).
Binding was detected by measuring an increase in the contact area between the two partners. Such an area has been calculated by rolling a spherical probe with a diameter of 1.4 Angstroms on the surface of the partners alone and in complex. The resulting contact area is therefore given by half of the difference between the solvent accessible areas calculated for the complex and the

sum of the solvent accessible areas calculated for each binding partner unbound ((SAS[complex] - (SAS[Importinβ] + SAS[Nup153]))/2) (**Figure 4**). Importantly, the ability to bind, as well as the time of binding, did not appear to be correlated to the initial distance at which the binding partners were placed in the simulation system.

MD simulations Nup153FG^{FxFG} in the presence of Importinβ^{N}. The binding of Nup153FG^{FxFG} to Importinβ^{N} was sampled as previously described for the simulations involving Importinβ^{N} and Nup153FG^{PxFG}. Nevertheless, in this case the size of the system was different for different simulations in order to enhance computational efficiency. Indeed the size of the solvent box in which the binding partners were incubated varied accordingly to their distance, yielding systems with a different number of atoms ranging between 166,162 and 520, 540 particles.

BD simulations Nup153FG^{PxFG} in the presence of Importinβ^{N}. To perform BD simulations, the binding partners in the Nup153FG^{PxFG}•Importinβ^{N} complex, obtained from MD, were separated and placed at an initial distance of 40 nm. The analysis of the complex obtained from MD allowed the identification of a set of contacts used to define the formation of fruitful complexes between the partners. A fruitful complex was recorded when 1 or more contacts (up to 4) were independently satisfied according to the distances shown in **Table S2E** and set after a structural analysis of the obtained complex. Rotational and translational diffusion coefficients of the two binding partners were retrieved experimentally and used for the simulation of diffusional association. 25,000 trajectories were generated in each run and simulations were performed to sample, independently, the association of Importinβ^{N} and Importinβ$^{I178D/Y255A}$ to Nup153^{PxFG}. In order to test the contribution of apolar desolvation and electrostatics to the estimated reaction rates hydrophobic and electrostatic interactions were ignored. The determination of the rate describing the association of Importinβ^{N} and Importinβ$^{I178D/Y255A}$ and Nup153FG^{PxFG} was followed by a bootstrap analysis of the results to determine the standard deviations of the computed rates.

Supplemental Tables

Table S1, Related to Figure 4: Nup153FG^{PxFG}-Importinβ^{N} binding - details from MD simulations. Summary of the different simulations of Nup153FG^{PxFG} in presence of Importinβ^{N} both with AMBER99-sb* and CHARMM22* force fields. Time of binding is defined as in **Supplemental Experimental Procedures** and binding positions are numbered as in (Isgro and Schulten, 2005). 'New' indicates binding spots not previously identified, and 'not known binding spot' indicates that this spot does not directly involve HEAT repeats

**Force field**	**Cluster ID - Simulation ID**	**Binding**	**Time of binding**	**Binding position on Importinβ**
AMBER	7811 - 1	Yes	13 ns	#1 (HEAT repeats 3/4)
AMBER	7811 - 2	Yes	2 ns	#1 (Heat repeats 3/4)
AMBER	7811 - 3	No	-	-
AMBER	7811 - 4	No	-	-
AMBER	7811 - 5	Yes	12 ns	#5 (HEAT repeats 8/9)
CHARMM	3450 - 1	Yes	67 ns	#1 (HEAT repeats 3/4)
CHARMM	3450 - 2	Yes	50 ns	#2, #3<br>(HEAT repeats 5/6, 6/7)
CHARMM	3450 - 3	Yes	37 ns	Not known binding spot
CHARMM	3450 - 4	No	-	-
CHARMM	3450 - 5	No	-	-
AMBER	2635 - 1	Yes	16 ns	#1, new<br>(HEAT repeats 3/4, 4/5)
AMBER	2635 - 2	No	-	-
AMBER	2635 - 3	Yes	10 ns	#1, new<br>(HEAT repeats 3/4, 4/5)
AMBER	2635 - 4	Yes	30 ns	#2<br>(HEAT repeats 5/6)
AMBER	2635 - 5	Yes	42 ns	Not known binding spot
CHARMM	3591 - 1	Yes	12 ns	new (HEAT repeats 10/11)
CHARMM	3591 - 2	No	-	-
CHARMM	3591 - 3	No	-	-
CHARMM	3591 - 4	No	-	-
CHARMM	3591 - 5	No	-	-

Supplemental References:

Akke, M., and Palmer, A.G. (1996). Monitoring Macromolecular Motions on Microsecond to Millisecond Time Scales by R1ρ−R1 Constant Relaxation Time NMR Spectroscopy. Journal of the American Chemical Society *118*, 911-912.

Bayliss, R., Littlewood, T., and Stewart, M. (2000). Structural basis for the interaction between FxFG nucleoporin repeats and importin-beta in nuclear trafficking. Cell *102*, 99-108.

Bednenko, J., Cingolani, G., and Gerace, L. (2003). Importin beta contains a COOH-terminal nucleoporin binding region important for nuclear transport. The Journal of cell biology *162*, 391-401.

Bernado, P., and Blackledge, M. (2009). A self-consistent description of the conformational behavior of chemically denatured proteins from NMR and small angle scattering. Biophys J *97*, 2839-2845.

Bui, K.H., von Appen, A., DiGuilio, A.L., Ori, A., Sparks, L., Mackmull, M.T., Bock, T., Hagen, W., Andres-Pons, A., Glavy, J.S.*, et al.* (2013). Integrated structural analysis of the human nuclear pore complex scaffold. Cell *155*, 1233-1243.

Dedecker, P., Duwe, S., Neely, R.K., and Zhang, J. (2012). Localizer: fast, accurate, open-source, and modular software package for superresolution microscopy. Journal of biomedical optics *17*, 126008.

Delaglio, F., Grzesiek, S., Vuister, G.W., Zhu, G., Pfeifer, J., and Bax, A. (1995). NMRPipe: a multidimensional spectral processing system based on UNIX pipes. Journal of biomolecular NMR *6*, 277-293.

Ferreon, A.C., Gambin, Y., Lemke, E.A., and Deniz, A.A. (2009). Interplay of alpha-synuclein binding and conformational switching probed by single-molecule fluorescence. Proc Natl Acad Sci U S A *106*, 5645-5650.

Finlay, D.R., Newmeyer, D.D., Price, T.M., and Forbes, D.J. (1987). Inhibition of in vitro nuclear transport by a lectin that binds to nuclear pores. The Journal of cell biology *104*, 189-200.

Frey, S., and Gorlich, D. (2007). A saturated FG-repeat hydrogel can reproduce the permeability properties of nuclear pore complexes. Cell *130*, 512-523.

Hansen, D.F., Vallurupalli, P., and Kay, L.E. (2008). An improved 15N relaxation dispersion experiment for the measurement of millisecond time-scale dynamics in proteins. The journal of physical chemistry B *112*, 5898-5904.

Hillger, F., Hanni, D., Nettels, D., Geister, S., Grandin, M., Textor, M., and Schuler, B. (2008). Probing protein-chaperone interactions with single-molecule fluorescence spectroscopy. Angewandte Chemie *47*, 6184-6188.

Isgro, T.A., and Schulten, K. (2005). Binding dynamics of isolated nucleoporin repeat regions to importin-beta. Structure *13*, 1869-1879.

Jensen, M.R., Salmon, L., Nodet, G., and Blackledge, M. (2010). Defining conformational ensembles of intrinsically disordered and partially folded proteins directly from chemical shifts. Journal of the American Chemical Society *132*, 1270-1272.

Jorgensen, W.L., Chandrasekhar, J., Madura, J.D., Impey, R.W., and Klein, M.L. (1983). Comparison of simple potential functions for simulating liquid water. The Journal of Chemical Physics *79*, 926-935.

Jung, Y.S., and Zweckstetter, M. (2004). Mars -- robust automatic backbone assignment of proteins. Journal of biomolecular NMR *30*, 11-23.

Kose, S., Funakoshi, T., and Imamoto, N. (2015). Reconstitution of nucleocytoplasmic transport using

digitonin-permeabilized cells. Methods in molecular biology *1262*, 291-303.

Kramer, R.H., and Karpen, J.W. (1998). Spanning binding sites on allosteric proteins with polymer-linked ligand dimers. Nature *395*, 710-713.

Lindorff-Larsen, K., Piana, S., Palmo, K., Maragakis, P., Klepeis, J.L., Dror, R.O., and Shaw, D.E. (2010). Improved side-chain torsion potentials for the Amber ff99SB protein force field. Proteins *78*, 1950-1958.

Mercadante, D., Milles, S., Fuertes, G., Svergun, D.I., Lemke, E.A., and Grater, F. (2015). Kirkwood-Buff Approach Rescues Overcollapse of a Disordered Protein in Canonical Protein Force Fields. The journal of physical chemistry B *119*, 7975-7984.

Milles, S., Huy Bui, K., Koehler, C., Eltsov, M., Beck, M., and Lemke, E.A. (2013). Facilitated aggregation of FG nucleoporins under molecular crowding conditions. EMBO reports *14*, 178-183.

Milles, S., and Lemke, E.A. (2011). Single molecule study of the intrinsically disordered FG-repeat nucleoporin 153. Biophys J *101*, 1710-1719.

Milles, S., and Lemke, E.A. (2014). Mapping multivalency and differential affinities within large intrinsically disordered protein complexes with segmental motion analysis. Angewandte Chemie *53*, 7364-7367.

Milles, S., Tyagi, S., Banterle, N., Koehler, C., VanDelinder, V., Plass, T., Neal, A.P., and Lemke, E.A. (2012). Click strategies for single-molecule protein fluorescence. Journal of the American Chemical Society *134*, 5187-5195.

Ori, A., Banterle, N., Iskar, M., Andres-Pons, A., Escher, C., Khanh Bui, H., Sparks, L., Solis-Mezarino, V., Rinner, O., Bork, P., *et al.* (2013). Cell type-specific nuclear pores: a case in point for context-dependent stoichiometry of molecular machines. Molecular systems biology *9*, 648.

Otsuka, S., Iwasaka, S., Yoneda, Y., Takeyasu, K., and Yoshimura, S.H. (2008). Individual binding pockets of importin-beta for FG-nucleoporins have different binding properties and different sensitivities to RanGTP. Proc Natl Acad Sci U S A *105*, 16101-16106.

Ozenne, V., Bauer, F., Salmon, L., Huang, J.R., Jensen, M.R., Segard, S., Bernado, P., Charavay, C., and Blackledge, M. (2012). Flexible-meccano: a tool for the generation of explicit ensemble descriptions of intrinsically disordered proteins and their associated experimental observables. Bioinformatics *28*, 1463-1470.

Piana, S., Donchev, A.G., Robustelli, P., and Shaw, D.E. (2015). Water dispersion interactions strongly influence simulated structural properties of disordered protein States. The journal of physical chemistry B *119*, 5113-5123.

Piana, S., Lindorff-Larsen, K., and Shaw, D.E. (2011). How robust are protein folding simulations with respect to force field parameterization? Biophys J *100*, L47-49.

Pronk, S., Páll, S., Schulz, R., Larsson, P., Bjelkmar, P., Apostolov, R., Shirts, M.R., Smith, J.C., Kasson, P.M., van der Spoel, D., *et al.* (2013). GROMACS 4.5: a high-throughput and highly parallel open source molecular simulation toolkit. Bioinformatics *29*, 845-854.

Rasia, R.M., Lescop, E., Palatnik, J.F., Boisbouvier, J., and Brutscher, B. (2011). Rapid measurement of residual dipolar couplings for fast fold elucidation of proteins. Journal of biomolecular NMR *51*, 369-378.

Schaffer, J., Volkmer, A., Eggeling, C., Subramaniam, V., Striker, G., and Seidel, C.A.M. (1999). Identification of single molecules in aqueous solution by time-resolved fluorescence anisotropy. J Phys Chem A *103*, 331-336.

Schoch, R.L., Kapinos, L.E., and Lim, R.Y. (2012). Nuclear transport receptor binding avidity triggers a self-healing collapse transition in FG-nucleoporin molecular brushes. Proc Natl Acad Sci U S A *109*,

16911-16916.

Shammas, S.L., Travis, A.J., and Clarke, J. (2014). Allostery within a transcription coactivator is predominantly mediated through dissociation rate constants. Proc Natl Acad Sci U S A *111*, 12055-12060.

Shen, Y., and Bax, A. (2007). Protein backbone chemical shifts predicted from searching a database for torsion angle and sequence homology. Journal of biomolecular NMR *38*, 289-302.

Solyom, Z., Schwarten, M., Geist, L., Konrat, R., Willbold, D., and Brutscher, B. (2013). BEST-TROSY experiments for time-efficient sequential resonance assignment of large disordered proteins. Journal of biomolecular NMR *55*, 311-321.

Soranno, A., Buchli, B., Nettels, D., Cheng, R.R., Muller-Spath, S., Pfeil, S.H., Hoffmann, A., Lipman, E.A., Makarov, D.E., and Schuler, B. (2012). Quantifying internal friction in unfolded and intrinsically disordered proteins with single-molecule spectroscopy. Proc Natl Acad Sci U S A *109*, 17800-17806.

Svergun, D., Barberato, C., and Koch, M.H.J. (1995). CRYSOL - A program to evaluate x-ray solution scattering of biological macromolecules from atomic coordinates. J Appl Crystallogr *28*, 768-773.

Teraoka, I. (2002). Polymer solutions : an introduction to physical properties (New York: Wiley).

Wagner, R.S., Kapinos, L.E., Marshall, N.J., Stewart, M., and Lim, R.Y. (2015). Promiscuous binding of Karyopherinbeta1 modulates FG nucleoporin barrier function and expedites NTF2 transport kinetics. Biophys J *108*, 918-927.

Zhang, H., Neal, S., and Wishart, D.S. (2003). RefDB: a database of uniformly referenced protein chemical shifts. Journal of biomolecular NMR *25*, 173-195.

Zweckstetter, M., Hummer, G., and Bax, A. (2004). Prediction of charge-induced molecular alignment of biomolecules dissolved in dilute liquid-crystalline phases. Biophys J *86*, 3444-3460.
